# Human iPSC‐Derived Mononuclear Phagocytes Improve Cognition and Neural Health across Multiple Mouse Models of Aging and Alzheimer's Disease

**DOI:** 10.1002/advs.202417848

**Published:** 2025-08-24

**Authors:** V. Alexandra Moser, Luz Jovita Dimas‐Harms, Rachel M. Lipman, Jake Inzalaco, Shaughn Bell, Michelle Alcantara, Erikha Valenzuela, George Lawless, Simion Kreimer, Sarah J. Parker, Helen S. Goodridge, Clive N. Svendsen

**Affiliations:** ^1^ Board of Governors Regenerative Medicine Institute Cedars‐Sinai Medical Center 127 S San Vicente Blvd Los Angeles CA 900048 USA; ^2^ Smidt Heart Institute Department of Cardiology Cedars‐Sinai Medical Center 8700 Beverly Blvd Los Angeles CA 90048 USA

**Keywords:** aging, Alzheimer's disease, mononuclear phagocytes, neural health, serum amyloids

## Abstract

Young blood or plasma improves cognitive function in aged animals but has limited availability. The current study generates a subtype of young blood cells from easily expandable induced pluripotent stem cells and evaluates their effects on age‐ and Alzheimer's disease (AD)‐associated cognitive and neural decline. In aging mice, intravenous delivery of induced mononuclear phagocytes (iMPs) improves performance in hippocampus‐dependent cognitive tasks, increases neural health, and reduces neuroinflammation. Hippocampal single nucleus RNA‐sequencing shows that iMPs improve the health of a subpopulation of mossy cells that are critically involved in the type of cognitive task in which iMPs improve performance, and shows that iMPs decrease the transcriptional age of several hippocampal cell types. Plasma proteomic analyses reveal that iMPs can also reverse age‐associated increases in serum amyloid levels. This is verified in vitro, where iMP‐conditioned media is shown to protect human microglia against cell death induced by serum amyloids. Finally, iMPs improve cognition in both young and aging 5×FAD mice, highlighting their potential as a prevention as well as an intervention strategy. Together, these findings suggest that iMPs provide a novel therapeutic strategy to target both age‐ and AD‐related cognitive decline.

## Graphical abstract

Short‐term intravenous treatments with iPSC‐derived mononuclear phagocytes (iMPs) improve cognitive decline and neural health in two mouse models of aging and in the 5xFAD mouse model of Alzheimer's disease. iMPs reduce levels of serum amyloid proteins that increase in aging and improve the health of hippocampal microglia and mossy cells.

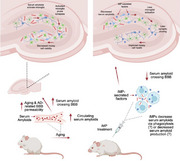

## 1 Introduction

As the elderly population increases, people are experiencing significant cognitive decline, both in healthy aging and in neurodegenerative diseases like Alzheimer's disease. AD is a progressive disorder that is characterized by the buildup of β‐amyloid (Aβ) plaques and neuronal loss,^[^ ^1^ ^]^ but also by neuroinflammation, which is increasingly seen as having a causative and disease‐accelerating role.^[^ ^2^ ^]^ Indeed, microglia, the main immune cells of the brain, are critically involved not only in disease progression, given their ability to phagocytose and clear Aβ,^[^ ^3^ ^]^ but are also implicated in disease initiation.^[^ ^4^ ^]^ Moreover, even in healthy aging, cognitive decline is linked with microglial activation^[^ ^5^ ^]^ and inflammation.^[^ ^6^ ^]^ Thus, there is a great need for therapeutics targeted at modulating neuroinflammation with the ultimate goal of enhancing cognition in aging and in AD.

Plasma from young mice^[^ ^7^ ^]^ or from human umbilical cord blood^[^ ^8^ ^]^ improves cognition and neural health in aged mice, and our lab found that transplanting bone marrow from young mice into aged, irradiated mice reversed age‐associated cognitive decline.^[^ ^9^ ^]^ Though the cognition‐enhancing effects of young plasma and bone marrow are promising, there are important drawbacks that limit their therapeutic potential. This includes the limited availability of these products, as well as the invasive nature of bone marrow transplantation. An alternative is using induced pluripotent stem cells (iPSCs) that can be generated from an individual's blood or tissue, rejuvenating aged cells in the process, and then differentiated into any cell type before re‐administration to that individual, permitting a true personalized therapeutic strategy. We hypothesized that mononuclear phagocytes, which include monocytes in blood and macrophages in tissues, may be involved in the effects of young plasma and bone marrow, either by producing rejuvenating factors or regulating production of pro‐aging or rejuvenating factors by other cells. As part of the immune system, these white blood cells locate tissue damage and infectious particles, and in response release inflammatory signals, phagocytose foreign particles, and promote tissue repair.^[^ ^10^ ^]^ However, during aging, mononuclear phagocytes show decreased clearance of debris and repair of damage, and an increased inflammatory response.^[^ ^11^ ^]^

The current study tested whether rejuvenated mononuclear phagocytes and/or the substances they produce are sufficient to preserve cognitive function in both aging and AD. Human iPSCs were differentiated into mononuclear phagocytes (iMPs) and administered intravenously to mouse models of aging and to the well‐characterized 5xFAD mouse model of AD,^[^ ^12^ ^]^ at both early and later stages of AD‐like pathology. iMPs appeared to be rapidly filtered from the blood, with accumulation in peripheral tissues but not in the brain. iMP treatment improved performance in hippocampus‐dependent behavioral tasks and had significant benefits on several key indices of microglial and neural health. Single nucleus RNA sequencing (snRNA‐seq) of the hippocampus revealed that iMP treatment restored hippocampal mossy cells, which are critical for the type of cognition that is improved in iMP‐treated animals. A machine learning model demonstrated that iMPs significantly reduced the predicted transcriptional age of 9 out of 15 hippocampal cell types. Finally, using a plasma proteomic analysis, we found that iMP treatment reversed aging‐associated increases in serum amyloid levels, and we confirmed the protective effects of iMPs against serum amyloids in vitro. These findings suggest that iMPs provide a novel individualized therapeutic strategy to target age‐ and AD‐related cognitive and neural decline.

## 2 Results

### 2.1 iMP Treatment Improves Aging‐Associated Declines in Cognition

**Figure 1:**
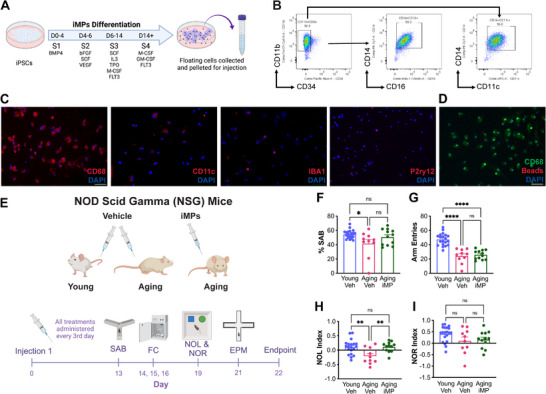
iMP treatment improves aging‐associated declines in cognition. A) Schematic showing the differentiation of human iPSCs to induced mononuclear phagocytes (iMPs) through 4 stages (S1‐S4). B) Flow cytometry analysis showing that the majority of iMPs (90.9%) are CD11b+/CD34‐ and have high expression of CD14, CD16, and CD11c. C) Images of immunocytochemical labeling of iMPs with CD68, CD11c, IBA1, and P2ry12 (red) and counterstained with DAPI (blue). Scale bar = 50 µm. D) To assess phagocytosis, iMPs were treated with fluorescently labeled microbeads (red) and labeled with CD68 (green). Scale bar = 50 µm. E) Schematic showing NSG mice were treated with either vehicle (saline) or live iMPs (500,000 cells/injection) every 3rd day for a total of 8 injections. Behavior was evaluated at the indicated timepoints. F) Aging vehicle‐treated mice had significantly fewer complete alternations than young mice on spontaneous alternation behavior (SAB), which was partially reversed in iMP‐treated aging male mice. G) Both aging vehicle‐ and iMP‐treated mice made significantly fewer arm entries than young mice. H) Aging vehicle‐treated mice spent significantly less time in the novel object location (NOL) than did young, and iMP treatment reversed this age‐associated decline. I) There were no significant differences among groups in novel object recognition (NOR) performance. Data are presented as mean (±SEM) values; *n* = 10‐20/group. * *p* < 0.05, ** *p* < 0.01, **** *p* < 0.0001, ns = not significant; one‐way ANOVA with Tukey's multiple comparisons test correction.

The well‐characterized 83i iPSC line^[^ ^13^ ^]^ was used to generate mononuclear phagocytes using a modified version of an established 14‐day protocol, with exposure to cytokines through four media stages (**Figure** **1**A), during which time adherent cysts formed.^[^ ^14^, ^15^ ^]^ After 14 days, non‐adherent iMPs, which budded off the cysts, were harvested weekly. Cell counts were fairly stable until about 10 weeks, when they began to decline, and while viability was lower during the first two weeks, this increased and remained stable for up to 15 weeks (Figure S1A,B, Supporting Information). Flow cytometry showed that most iMPs expressed the mature myeloid marker, CD11b, and were largely negative for the hematopoietic stem cell marker, CD34 (Figure 1B). This CD11b+ population had high expression of the mononuclear phagocyte markers CD14, CD16, and CD11c. Expression of stem cell marker TRA‐1‐60 and hematopoietic stem cell marker CD34 was low at all time points, whereas levels of CD11b and CD14 were consistently high across time, and levels of CD16, CD45, and CD163 increased from 4 to 8 weeks and then remained stable at 20 weeks (Figure S1C, Supporting Information). Immunocytochemistry confirmed expression of monocyte/macrophage markers CD68 and CD11c, but low levels of microglial markers IBA1 and P2ry12 (Figure 1C). Cells engulfed fluorescently labelled bioparticles, demonstrating their phagocytic ability (Figure 1D).

NOD scid gamma (NSG) mice are genetically immunocompromised to permit survival of human xenografts,^[^ ^16^ ^]^ and show an accelerated aging phenotype, with deficits in long‐term potentiation and cognitive decline by 11–12 months.^[^ ^8^ ^]^ Male and female NSG mice aged to 3‐ (young) or 11–12 months (aging) were injected with 100 µL saline or 5 × 10^5^ iMPs in saline into the tail vein every 3^rd^ day for 21 days, for a total of 8 injections with behavioral testing beginning after the 5^th^ injection (Figure 1E). This treatment paradigm follows previous work that has demonstrated beneficial effects of young plasma in aging NSG mice.^[^ ^8^ ^]^ Body weights remained constant in all groups (Figure S2A, Supporting Information). All young mice were vehicle‐treated (referred to as young), and aging mice were treated with either vehicle (referred to as aging) or iMPs (referred to as iMP‐treated).

To establish any cognitive effects of iMP treatment, an extensive behavioral analysis was carried out on these animals. First, hippocampus‐dependent spatial working memory was assessed by spontaneous alternation behavior in the Y‐maze.^[^ ^17^ ^]^ Aging male mice performed significantly worse than young mice (Figure 1F), but this significant decline was not seen following just 5 iMP treatments (*p* = 0.61 relative to young mice). However, iMP‐treated animals were not significantly different compared to aging animals *(p* = 0.17). To ensure that effects of iMPs on behavioral outcomes did not depend on motor‐ or anxiety‐like‐behavior, mice were evaluated on total arm entries in the Y‐maze and on the elevated plus maze.^[^ ^18^ ^]^ In the Y‐maze, aging mice made significantly fewer total arm entries than young mice, regardless of treatment (Figure 1G), and in the elevated plus maze, the percent time spent in the open arm and percent of open arm entries were similar across ages and treatments (Figure S2B,C, Supporting Information).

Hippocampus‐dependent spatial short‐term memory declines with age, while recognition memory, largely dependent on the perirhinal cortex, is preserved.^[^ ^19^, ^20^ ^]^ Thus, the novel object location task was used to assess spatial short‐term memory^[^ ^21^ ^]^ after the 7^th^ treatment. In the novel object location task, aging mice performed significantly worse than young mice (Figure 1H). In contrast, iMP‐treated mice no longer performed worse than young mice (*p* = 1.00), and notably, performed significantly better than aging mice (*p* = 0.009) showing that only 7 iMP treatments administered over 19 days can fully restore short‐term spatial memory function in this aging model. Using the novel object recognition task to assess recognition memory showed that aging mice had a trend toward decreased time spent with a novel object, but this did not reach statistical significance (Figure 1I). Freezing behavior in the fear conditioning test examined contextual and cued recall memory, which were not affected by aging (Figure S2D–G, Supporting Information). Thus, we find that in male NSG mice, iMP treatment protects against age‐associated cognitive decline in hippocampus‐dependent tasks relying on spatial working‐ and short‐term‐memory, and these effects are due to cognitive improvements rather than to changes in motor activity or anxiety‐like behavior. In contrast to males, female mice did not display aging‐associated changes in spontaneous alternation behavior or in arm entries in the Y maze (Figure S2H,I, Supporting Information). While aging female mice showed deficits in novel object location (mean = −0.02) versus young mice (mean = 0.25), and iMP‐treated females performed better (mean = 0.13), these differences did not reach statistical significance (Figure S2J, Supporting Information).

### 2.2 iMP Treatment Improves Microglial and Neural Health

**Figure 2:**
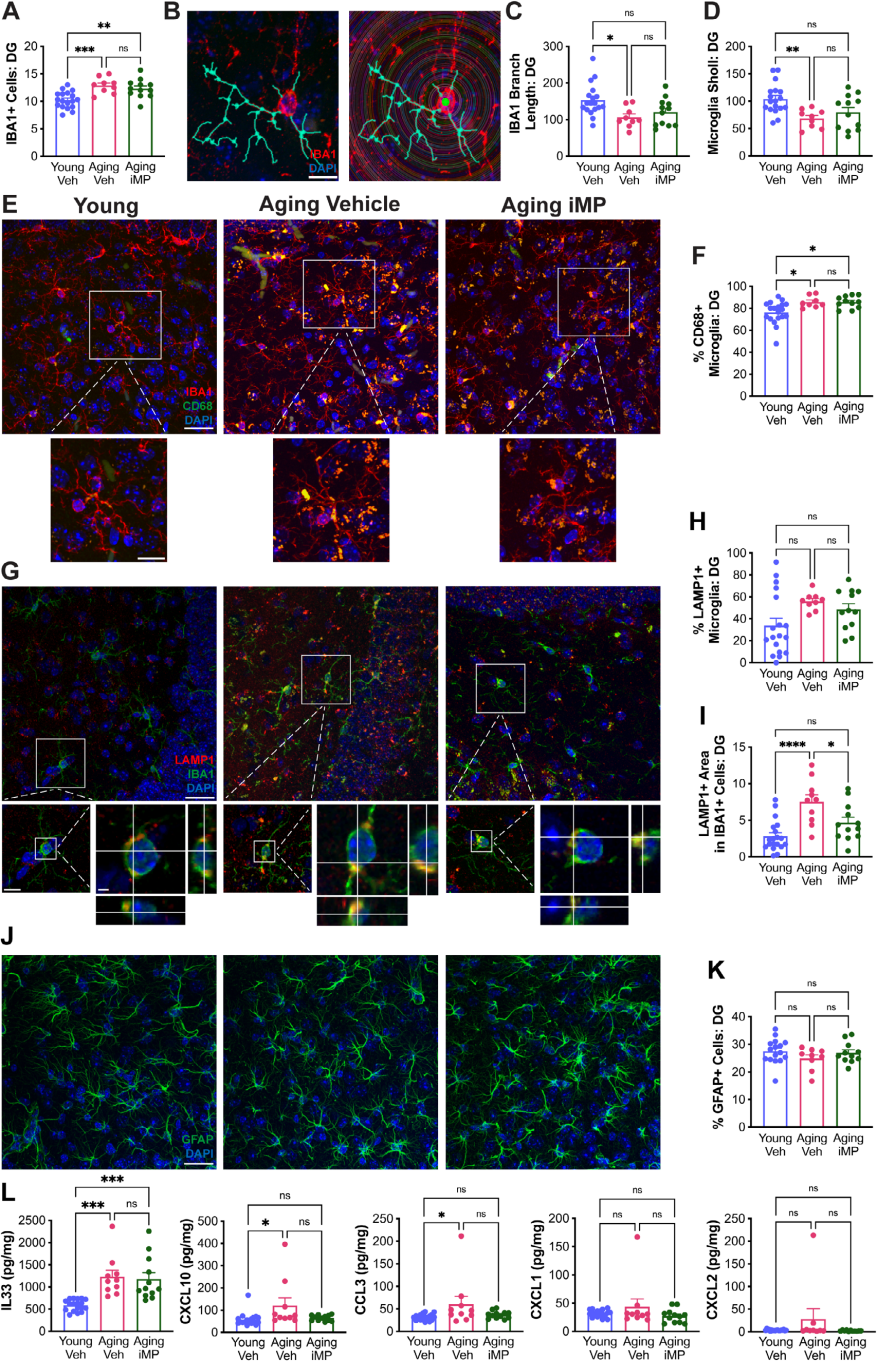
iMP treatment improves microglial and neural health. A) Total IBA1+ cells were increased in hippocampal dentate gyrus (DG) of aging mice. B) Representative images of a traced cell (left), and of Sholl analysis (right). C) Compared to young mice, microglial branch length was significantly reduced in vehicle‐treated, but not iMP‐treated, aging mice. An average of 10 IBA+ cells per mouse was analyzed for branch length; each dot shows average data from one mouse. D) Sholl analysis of traced microglia shows significantly decreased complexity in aging vehicle‐ but not aging iMP‐treated versus young mice. E) Images showing staining for the microglial marker IBA1 (red) and lysosomal marker CD68 (green); scale bar = 25 µm on top image and 5 µm on bottom image. F) Aging increased the percent of CD68+ microglia, regardless of treatment. G) Images showing staining for the microglial marker IBA1 (green) and LAMP1 (red); scale bar = 25 µm on top image, 10 µm on bottom image, and 2 µm on orthogonal view. H,I) While the percent of IBA+ microglia expressing LAMP1 was not significantly different across groups, aging mice had a significant increase in the area of LAMP1 immunoreactivity within microglia, which was significantly decreased in iMP‐treated aging mice. J) Images of staining for the astrocyte marker GFAP in hippocampal DG. Scale bar = 25 µm. K) The percent of GFAP+ cells does not vary with aging or treatment. L) Levels of inflammatory cytokines and chemokines were evaluated by multi‐plex immunoassays in cortical lysates. Levels of IL33 were significantly increased across both aging groups, while CXCL10 and CCL3 were only significantly increased in vehicle‐ but not in iMP‐treated aging mice. Levels of CXCL1 and CXCL2 did not differ across groups. Data are presented as mean (±SEM) values; *n* = 10‐20/group. * *p* < 0.05, ** *p* < 0.01, *** *p* < 0.001, **** *p* < 0.0001, ns = not significant; one‐way ANOVA with Tukey's multiple comparisons test correction.

Mice were sacrificed after 8 injections, and several key indices of microglial and neural health were assessed in the hippocampus to test whether iMPs could affect pathological changes associated with aging. IBA1 immunohistochemistry was used to assess the number and morphology of hippocampal microglia, as these measures are both affected by aging.^[^ ^22^, ^23^ ^]^ Aging mice had increased numbers of microglia in the dentate gyrus (DG), regardless of treatment (**Figure** **2**A). Microglial processes were traced and Scholl analysis was performed (Figure 2B), as branches contract when microglia become activated.^[^ ^24^ ^]^ Both branch length and complexity were decreased with aging, and iMP treatment reversed these microglial activation phenotypes, such that iMP‐treated mice were not different from young mice (Figure 2C,D). Microglia expressing CD68, a lysosomal marker that is increased upon microglial activation,^[^ ^25^ ^]^ were significantly higher in aging mice, regardless of treatment (Figure 2E,F). LAMP1 is upregulated in both aging^[^ ^26^ ^]^ and AD.^[^ ^27^ ^]^ While the percent of IBA1+ cells expressing LAMP1 did not differ across groups, the area of LAMP1+ signal inside microglia was significantly increased with aging, which, notably, significantly decreased following iMP treatment (Figure 2G–I).

Several of these measures were also evaluated in hippocampal CA1 and CA3. There was an overall increase in microglial number with aging, with CA1 showing increases specifically in aging vehicle‐treated mice, and CA3 showing increases in both aging groups (Figure S3A,B, Supporting Information). Microglial branch length was significantly decreased in CA1 of both aging groups, but in CA3, it was specifically decreased in aging, but not in iMP‐treated, mice (Figure S3C,D, Supporting Information). Compared to young mice, aging mice but not iMP‐treated mice, showed significantly decreased microglial complexity in both CA1 and CA3 (Figure S3E,F, Supporting Information). CD68 expression was similar across groups in CA1 microglia, while in CA3, there was a significant aging‐associated increase, and a trend toward a decrease in iMP‐treated mice (Figure S3G,H, Supporting Information). LAMP1 expression was significantly increased in CA1 of both aging and iMP‐treated mice, and while there was a significant main effect on the percent of microglia expressing LAMP1 in CA3, this was not significant in post‐hoc comparisons (Figure S3I,J, Supporting Information).

Cyclooxygenase 1 and 2 (COX‐1/2) were assessed in hippocampal CA3. COX‐1, expressed in microglia, is upregulated in AD and leads to increased synthesis of prostaglandin E_2_, thereby leading to initiation and propagation of inflammation.^[^ ^28^ ^]^ COX‐2, largely expressed in neurons, is central to excitotoxic death.^[^ ^29^, ^30^ ^]^ Aging mice had significantly increased microglial COX‐1 expression compared to young mice, which was no longer significant with iMP treatment (Figure S3K,L, Supporting Information). The percent area positive for COX‐2 was similar across ages and treatments (Figure S3M,N, Supporting Information), suggesting aging and iMP treatment alter microglial COX‐1 signaling, but not neuronal COX‐2 signaling.

Quantifying levels of inflammatory cytokines and chemokines in cortical tissue showed that while CXCL10 and CCL3 are significantly increased in aging mice, they are not significantly different between young and aging iMP‐treated mice (Figure 2L). Aging was also associated with a significant increase in levels of IL33, which remained elevated in iMP‐treated animals. CXCL1, CXCL2, IFNγ, IL10, TNFα, IL1β, and IL6 did not differ with either aging or iMP treatment (Figure 2L; Figure S3O, Supporting Information).

Markers of neuronal health were also stained for and quantified by immunohistochemistry. VGLUT1, a marker of postsynaptic glutamatergic vesicles, decreases in the hippocampus with age^[^ ^31^ ^]^ and correlates with performance in learning and memory tasks.^[^ ^32^ ^]^ Aging mice showed a significant decrease in VGLUT1 expression in hippocampal CA3 that was improved with iMP treatment, to levels not significantly different from young mice (Figure S3P,Q, Supporting Information). Astrogliosis, based on the number and soma size of astrocytes, increases with aging.^[^ ^33^ ^]^ Astrocyte numbers did not show changes with either treatment or aging in DG (Figure 2J,K). Likewise, astrocyte numbers and soma size did not differ in hippocampal CA1 (Figure S3S,T, Supporting Information). In hippocampal CA3, however, aging mice had significantly increased numbers of GFAP‐positive astrocytes compared to young mice, which was no longer significant in iMP‐treated mice (Figure S3R,U, Supporting Information). Astrocyte soma size in CA3 did not differ with age or treatment (Figure S3V, Supporting Information).

Meanwhile, female NSG mice generally did not show age‐associated decreases in neural health markers, apart from a significant decrease in microglial branch length that, notably, was restored after iMP treatment (Figure S3W–Z, Supporting Information).

We also tested effects of iMP treatment in the context of early age‐associated changes by using immune‐competent 11–13‐month‐old male BALB/c mice, which are considered to be middle‐aged and initiating the aging process.^[^ ^34^ ^]^ Cyclosporin A‐immune suppressed mice were injected with either 100 µL saline (vehicle) or 5 × 10^5^ iMPs in saline into the tail vein twice weekly for either 4 or 10 weeks; all mice were tested on novel object location at the same timepoint as used for NSG mice, and a subset of animals was treated for an additional 6 weeks and then further tested on the novel object location and recognition tasks at 10 weeks of treatment. Control groups included young mice (3‐months‐old) treated with saline, and a group of aging mice treated with heat inactivated iMPs (referred to as “dead iMPs”) to test for possible inflammatory effects of injecting human cell debris (Figure S4A, Supporting Information). While body weights in young mice fluctuated and overall increased over time, treatment did not significantly affect weight in aging mice (Figure S4B, Supporting Information). Compared to young mice, there were trends toward decreased novel object task performance in vehicle‐treated aging and dead‐iMP‐treated aging mice, but not in iMP‐treated aging mice; however, as reported previously^[^ ^35^, ^36^ ^]^ behavior was highly variable in this strain and the differences did not reach statistical significance (Figure S4C–E, Supporting Information).

IBA1 staining in hippocampal DG showed that branch length and complexity were significantly decreased in aging vehicle‐treated mice, with a trend toward a decrease in aging dead iMP‐treated mice (Figure S4F–I, Supporting Information). Notably, aging BALB/c mice treated with live iMPs had significantly greater microglial branch length and complexity than aging vehicle‐treated mice and did not differ from young mice. The total number of microglia was increased specifically in aging dead‐iMP‐treated mice, while levels of CD68 were increased in all aging BALB/c mice (Figure S4J,K, Supporting Information). These results show that iMP treatment protected against aging‐associated changes in microglial health that occur before significant behavioral deficits, suggesting that the treatment is effective in reducing signals that activate microglia at very early stages of aging. However, conducting studies at a later time point in this strain of mice was not practical due to challenges associated with immune suppression in aging mouse colonies.

Collectively, we find that iMP treatment rescues aging‐associated impairments in hippocampus‐dependent cognition and neural health in male NSG mice and significantly improves measures of microglial health in both male BALB/c and NSG mice, as well as in female NSG mice.

### 2.3 iMP Treatment Increases a Subpopulation of Mossy Cells Lost in Aging Male NSG Mice

**Figure 3:**
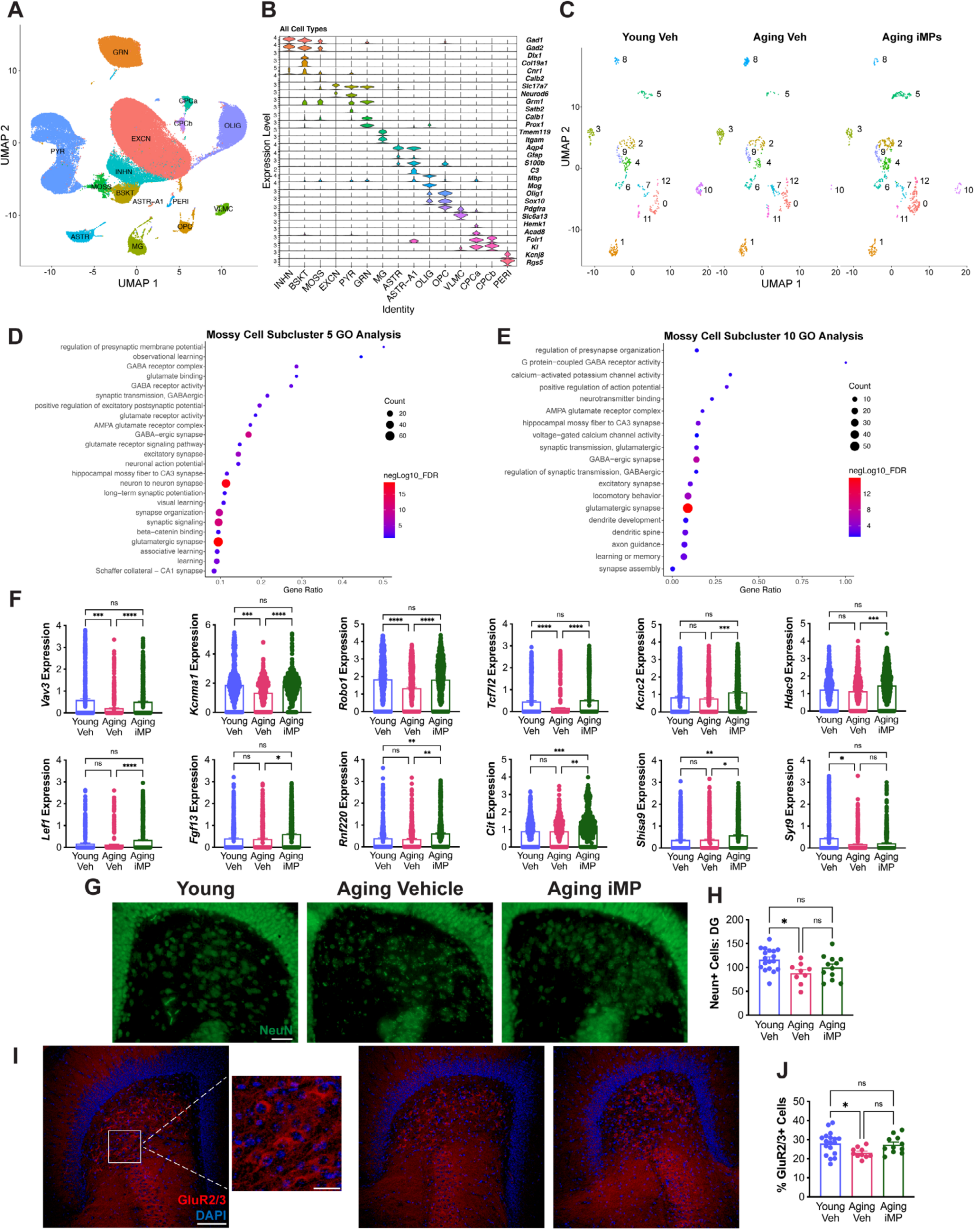
iMP treatment increases a subpopulation of mossy cells lost in aging male NSG mice. A) Clustering of samples by principal component analysis (PCA) and UMAP for dimensionality reduction and visualization. Clustering analysis revealed 15 distinct clusters representing 8 cell types including 6 clusters expressing neuronal markers, 2 clusters expressing astrocytic markers, 2 clusters expressing choroid plexus cell markers, and 1 cluster each expressing markers of microglia (MG), oligodendrocytes (OLIG), oligodendrocyte precursor cells (OPC), vascular leptomeningeal cells (VLMCs), and pericytes (PERI). B) Violin plot showing expression levels of representative cell‐type‐enriched marker genes across 15 cell types. C) Subclustering of mossy cells by PCA and UMAP, and split by group, reveals that subclusters 5 and 10 are reduced in vehicle‐treated aging mice, but restored with iMP treatment. D‐E) Gene ontology (GO) analyses on the genes enriched in (D) mossy cell subcluster 5 and (E) subcluster 10 (at *p* < 0.05 and positive log2FC, relative to all other mossy cell subclusters); select GO terms are shown. The size of dots reflects the number of genes from mossy cell subclusters 5 and 10 that are part of each GO term, while the color reflects the ‐10log FDR. F) Expression of select genes from the top 25 enriched genes in mossy cell subclusters 5 and 10 was compared across ages and treatments in all mossy cells. * *p* < 0.05, ** *p* < 0.01, *** *p* < 0.001, **** *p* < 0.0001, ns = not significant; pair‐wise DEG analysis between each group using the Wilcox Rank sum test. G) Images showing immunohistochemical staining for the neuronal nuclei marker NeuN in the dentate gyrus (DG). Scale bar = 25 µm. H) Quantification shows a significant decrease in NeuN+ cells in aging vehicle‐ but not iMP‐treated mice. I) Images showing staining of the mossy cell marker GluR2/3 in DG. Scale bar = 100 µm. Inset shows 25 µm scale bar. J) GluR2/3+ cells are significantly reduced in aging vehicle‐ but not iMP‐treated mice, as compared to young mice. Data are presented as mean (±SEM) values; *n* = 10‐19/group. * *p* < 0.05, ns = not significant; one‐way ANOVA with Tukey's multiple comparisons test correction.

To establish which hippocampal cells were affected by aging and iMP treatment, unbiased, high‐throughput snRNA‐seq was performed in a subset of mice (*n* = 5/group). Principal component analysis (PCA) was performed and UMAP was used for dimensionality reduction and visualization. Clustering analysis revealed 15 distinct clusters representing 8 cell types, including neurons, astrocytes, and microglia (MG), as well as oligodendrocytes (OLIG), oligodendrocyte precursor cells (OPC), vascular leptomeningeal cells (VLMCs), choroid plexus cells (CPCs), and pericytes (PERI) (**Figure** **3**A,B). Neuronal populations could further be divided into inhibitory neurons (INHN), excitatory neurons (EXCN), basket cells (BSKT), dentate gyrus mossy cells (MOSS), pyramidal neurons (PYR), and granule cells (GRN). Astrocytes (ASTR) were found to have two clusters; one expressing general astrocyte genes and one expressing genes associated with activated or A1 astrocytes (ASTR‐A1). Similar clustering of the identified cell populations was observed across treatment groups (Figure S5A, Supporting Information).

Differential gene expression (DEG) analyses showed that of the 15 hippocampal cell types, DG mossy cells showed the greatest DEGs between groups (Figure S5B, Supporting Information). These cells were recently shown to respond to novel environments and be critical for forming memories of novel contexts;^[^ ^37^, ^38^ ^]^ thus, we focused further on this population of mossy cells. The identity of these cells was first confirmed by validating that this population of neurons expressed marker genes previously identified for mossy cells. As shown in Figure S5C (Supporting Information), mossy cells had high expression of the canonical marker Calb2, as well as of Gria2 and Gria3,^[^ ^39^ ^]^ and were enriched for Pcp4.^[^ ^40^ ^]^ Calb2 can be expressed in rare populations of ectopic granule cells and inhibitory neurons;^[^ ^41^ ^]^ thus, we confirmed the absence of granule cell marker, Gabra6, and of inhibitory neuron markers Pvalb and Nos1. Sub‐clustering analysis on the 1760 cells that are part of the mossy cell cluster revealed that several subclusters differed across groups (Figure 3C). For instance, subcluster 5 was more densely populated in iMP‐treated aging mice while strikingly, subcluster 10, which was densely populated in young mice, was nearly absent in aging but not iMP‐treated mice. However, due to small numbers of cells within each subcluster and to high variability between animals, the number of cells in each subcluster did not reach statistically significant differences between groups. GO analyses performed on the genes enriched in subclusters 5 and 10 (adjusted *p*<0.05 and positive log2FC) revealed several terms relating to learning and memory, long‐term synaptic potentiation, and synaptic signaling (Figure 3D,E). A cellular component GO analysis revealed enrichment for several synapse‐related terms, including “hippocampal mossy fiber to CA3 synapse”, as well as for both GABAergic and glutamatergic synapses, which corresponds with the fact that mossy cells innervate both kinds of neurons.

Several of the most highly enriched genes in subclusters 5 and 10 were involved in learning and memory, neuronal excitability, and synaptic plasticity. Because these subclusters contained fewer cells from aging mice, gene expression of the top enriched genes in subclusters 5 and 10 was compared across ages and treatments in all mossy cells (Figure 3F). Several genes were significantly decreased in aging, but rescued with iMP treatment (*Vav3*, *Kcnma1*, *Robo1*, *Tcf7l2*), while others were increased specifically in iMP‐treated mice (*Kcnc2*, *Hdac9*, *Lef1*, *Fgf13*, *Rnf220*, *Cit*, *Shisa9*).

Based on results of snRNA‐seq showing changes in DG mossy cells, we next sought to validate these findings at the protein level by staining for and quantifying NeuN‐positive cells. Compared to young mice, the total neuron number was decreased in aging but not iMP‐treated mice, specifically in the hilus of the DG where mossy cells are found (Figure 3G,H), but not in hippocampal CA1 or CA3 (Figure S5D, Supporting Information). Two markers of mossy cells were assessed; while calretinin did not differ across ages or treatment (Figure S5E,F, Supporting Information), GluR2/3 was significantly decreased with aging, but was not different between young and iMP‐treated aging mice (Figure 3I,J).

Previous studies have found increased neurogenesis in response to heterochronic parabiosis.^[^ ^42^ ^]^ To determine whether iMP treatment was increasing neurogenesis in the current study, we examined the newborn neuron marker, doublecortin (DCX). *Dcx* gene expression in mossy cells was similar across groups, and DCX+ cell numbers in DG were significantly decreased in both vehicle‐ and iMP‐treated aging mice, suggesting that the behavioral effects of iMPs were not mediated by increased neurogenesis (Figure S5G Supporting Information).

In summary, snRNA‐seq reveals changes in aging hippocampus, including a decrease in specific mossy cell subpopulations characterized by genes involved in synaptic plasticity and cognition. Remarkably, these aging‐associated changes are partially or entirely reversed after only 21 days of iMP treatment.

### 2.4 iMP Treatment Reverses the Transcriptomic Age of Several Hippocampal Cell Types

**Figure 4:**
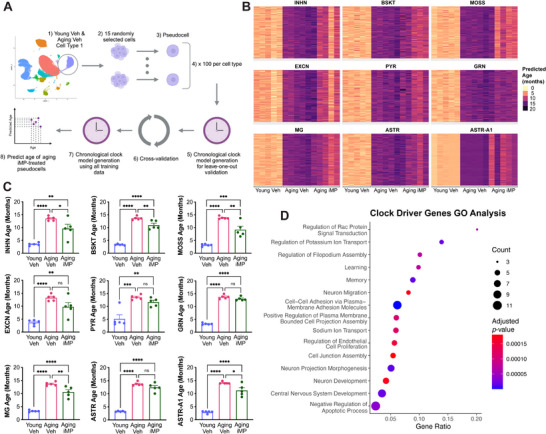
iMP treatment reverses the transcriptomic age of several hippocampal cell types. A) Schematic showing the generation of cell‐type specific clocks. 15 cells were randomly selected from each hippocampal cell type and combined to generate one pseudocell, which was repeated 100 times for each cell type and sample. The model was then trained on young versus old cells, and cross‐validation was performed before using the model to predict the age of cells from iMP‐treated samples. B) Heatmaps showing the predicted ages in months of 9 hippocampal cell types from each sample. All neuronal cell types as well as microglia (MG), astrocytes (ASTR), and A1‐astrocytes (ASTR‐A1) are shown. C) A number of hippocampal cell types, including the neuronal subtypes of inhibitory neurons (INHN), basket cells (BSKT), and mossy cells (MOSS), as well as MG and ASTR‐A1, were predicted to be significantly younger in aging iMP‐ than in aging vehicle‐treated samples. Data are presented as mean (±SEM) values; *n* = 5/group. * *p* < 0.05, ** *p* < 0.01, *** *p* < 0.001, **** *p* < 0.0001, ns = not significant; one‐way ANOVA with Tukey's multiple comparisons test correction. D) Gene ontology (GO) analysis on the 160 genes driving cell‐type specific clocks in at least 2 hippocampal cell types.

As an additional un‐biased assay, we used a machine learning model to determine whether iMP treatment affected the cellular “age” of any hippocampal subtypes. Specifically, cell type‐specific clocks were generated using a recently developed single‐cell regression model, which showed that both exercise and treatment with young plasma reduced the predicted age of select cell types.^[^ ^43^ ^]^ Briefly, 15 cells were randomly selected from each sample and cell type and combined to generate one pseudobulked “pseudocell”; this was repeated 100 times for each sample and cell type (**Figure** **4**A). We first ensured the validity of applying this model to our dataset by cross validation. Specifically, clocks were generated on 4 young and 4 aging vehicle‐treated animals and were then used to predict the age of the remaining sample from each group. Performing this five times, and omitting different animals each time, showed that the model reliably predicted young versus aging animals (Figure S6A, Supporting Information).

All 5 young and all 5 aging‐vehicle samples were then used to generate cell‐type specific clocks, which were applied to predict the age of iMP‐treated samples. We found a significant effect of iMPs in 9 out of 15 hippocampal cell types (Figure 4B,C; Figure S6B,C, Supporting Information). Specifically, aging iMP samples were predicted to be significantly younger than aging vehicle samples in the case of the neuronal subtypes of inhibitory neurons, basket cells, and, strikingly, mossy cells. Moreover, there were significant effects of iMPs in several non‐neuronal cells as well, including the ASTR‐A1, MG, OLIG, OPC, PERI, and VLMC clusters.

We then examined the genes that were driving cell‐specific clocks. Within the 9 cell types showing significant rejuvenating effects of iMPs, 160 clock‐driver genes were found in at least 2 cell types, with GO analysis showing enrichment for terms related to learning and memory as well as neuron development and migration (Figure 4D). 28 genes were shared by at least 3 cell types, including *Apoe*, the main genetic risk factor for AD. With subsequent analysis of these 28 genes using Enrichr,^[^ ^44^ ^]^ OMIM Disease showed enrichment in “Alzheimer's disease” (adj *p*‐value = 0.014; odds ratio = 89.13), while the Mouse Genome Informatics Mammalian Phenotype analysis revealed “abnormal spatial learning” as the most enriched term (adj *p*‐value = 0.003; odds ratio = 30.31). In summary, cell type‐specific clocks are driven by genes related to learning and memory, and AD, and iMPs reverse the transcriptomic age of more than half of hippocampal cell types such that the predicted age of cells from aging iMP‐treated samples was significantly younger than those from aging vehicle‐treated mice.

### 2.5 iMPs Remain in the Periphery and Alter Plasma Protein Expression

While there were significant cognitive and neural health improvements after iMP treatment, there was not a separate population of cells in snRNA‐seq data from iMP‐treated animals, as would be expected if human cells were to enter the brain parenchyma. To establish where iMPs accumulate after injection, green fluorescent protein (GFP)‐expressing iPSCs were differentiated into iMPs and administered at a high dose of 12 million cells via tail vein injection. At 30 minutes post‐infusion, blood and tissues were collected from uninjected control and injected mice. Flow cytometry revealed no GFP+ cells in the blood of the uninjected control or injected animals (Figure S7A, top, Supporting Information). In contrast, GFP+ iMPs spiked into the blood immediately after collection were detectable in both animals (Figure S7A, bottom, Supporting Information). Thus, iMPs do not remain in circulation 30 min post‐administration.

To rule out a lack of sensitivity of this assay to detect rare GFP+ cells, we next assessed the presence of human DNA in tissues by testing for human‐specific Alu elements. As a positive control, human DNA diluted as low as 1 pg in mouse DNA was detectable (Figure S7B, Supporting Information). Human DNA was not detected in uninjected mouse tissue but was found in several tissues following iMP administration (Figure S7C, Supporting Information), suggesting that iMPs do not remain in the circulatory system, but quickly enter various organs. This pattern was also observed in tissues from a subset of experimental iMP‐treated mice, where Alu repeats were most consistently observed in the lung (Figure S7D, Supporting Information). Accumulation of iMPs in lung tissue from experimental animals was further detected by immunohistochemical staining for the human‐specific marker, SC121 (Figure S7E, Supporting Information).

Additionally, iMPs were administered at a dose of 5 million cells and tissues were harvested either 24, 48, or 72 h later. iMPs were consistently detected only in lung at all time points (Figure S7F, Supporting Information), suggesting that a single dose of 5 million cells may be cleared rather quickly. In contrast, the finding of Alu repeats in multiple tissues of mice injected either a single time with 12 million cells or repeatedly with 500,000 cells indicates that a single very high dose or repeated low doses lead to accumulation in various organs. Pure genomic DNA isolated from 12 million iMPs was infused, and tissues or blood were harvested 30 min later. Human Alu elements were detected in the tail and blood spiked with human DNA immediately after collection, but not in other tissues or in plasma, serum, or unspiked blood (Figure S7G, Supporting Information). Detection of human Alu repeats in various organs after iMP infusion, but not after human DNA infusion, suggests that intact iMPs are collecting in these tissues rather than simply dying and releasing DNA into the animal.

To confirm the accumulation of iMPs in tissue, SC121 staining was performed on organs harvested from mice injected with 3 × 10^6^ iMPs. Figure S7H (Supporting Information) shows that cultured in vitro iMPs express SC121. After injection, SC121+ cells were found in the lung, kidney, heart, and spleen, but were not detected in the liver or brain (Figure S7H, Supporting Information). Thus, human iMPs appear to accumulate in various peripheral organs, but do not enter the brain.

**Figure 5:**
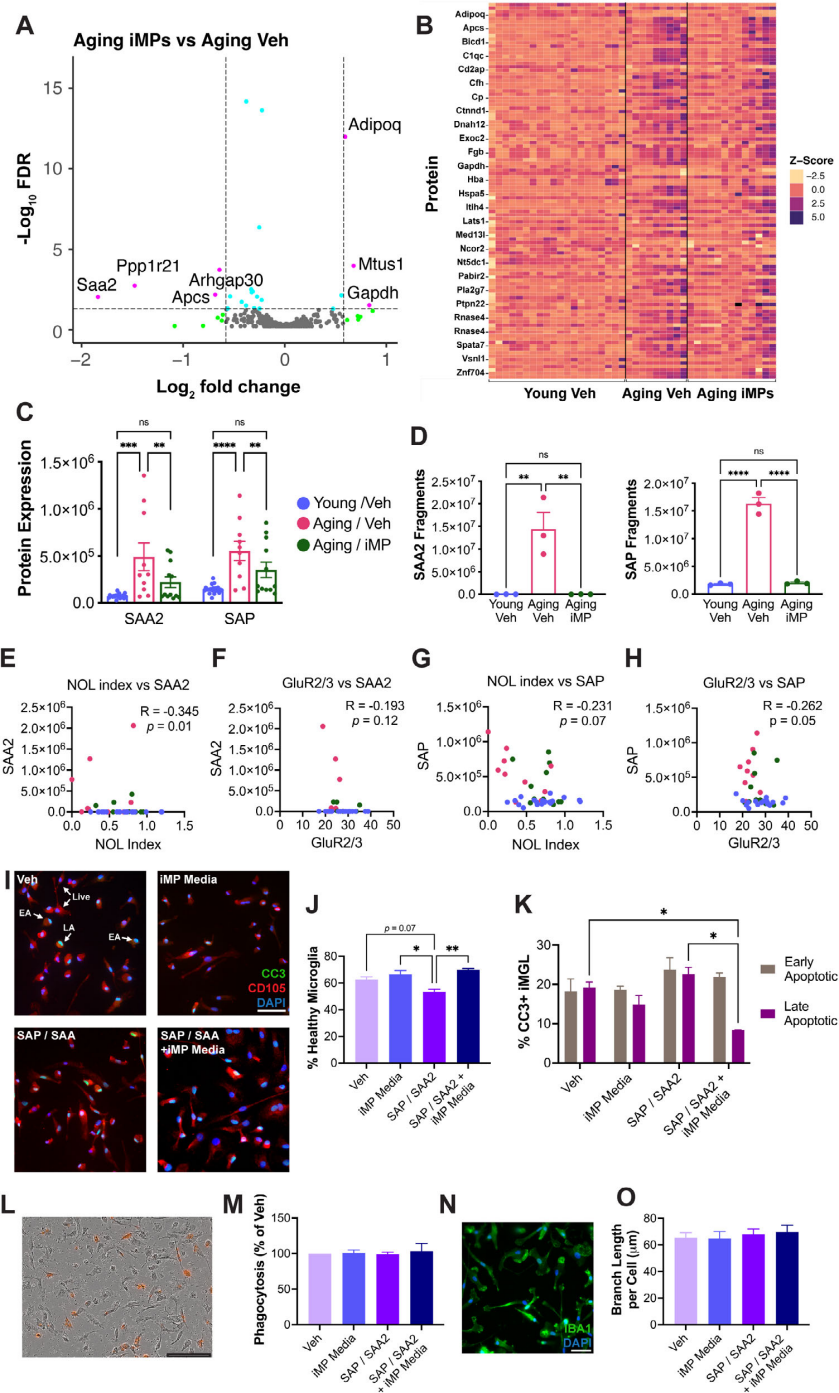
iMP treatment reverses age‐associated increases in plasma levels of serum amyloid proteins. A) Plasma samples were analyzed using mass spectrometry, and proteins differentially expressed between aging iMP‐treated and aging vehicle‐treated mice at FDR < 0.05 and at a fold change of 1.5 or greater are shown in a volcano plot. B) Heatmap of the 109 proteins that were differentially expressed between vehicle‐treated aging and young mice, with 75 being upregulated and 34 being downregulated with aging. C) Levels of serum amyloid A2 (SAA2) and serum amyloid P component (SAP or *Apcs*) are increased in aging but decreased after iMP treatment. D) PRM analysis of SAA2 and SAP confirms findings of discovery proteomics analysis. E‐H) Spearman r correlations between normalized novel object location index or mossy cell marker GluR2/3 and serum amyloid proteins SAA2 and SAP. For discovery proteomics, n = 10‐20/group; pair‐wise DEG analysis between each group using the Wilcox Rank sum test. For PRM, n = 3/group; one‐way ANOVA with Tukey's multiple comparisons test correction. I–O) iPSC‐derived microglia (iMGL) were treated with SAA2 and APCS, in either RPMI (Veh) or in iMP‐conditioned media. I) iMGL stained for the macrophage marker CD105 (red) and the apoptotic cell marker CC3 (green). Healthy cells have DAPI+ nuclei (blue), early apoptotic cells have teal nuclei with some expression of CC3, and late apoptotic cells have a green CC3+ nucleus. Scale bar = 50 µm. J) Treatment with serum amyloids decreased the number of DAPI+/CC3‐ healthy iMGL, while iMP‐conditioned media protected against the effects of serum amyloids. K) iMGL treated with SAA2 and APCS in iMP‐conditioned media had significantly less late apoptotic cells than those treated with serum amyloids in Veh media. L) iMGL treated with pHrodo zymosan beads. Orange spots are beads that have fluoresced upon being phagocytosed by a cell. Scale bar = 200 µm. M) Neither serum amyloids nor iMP‐conditioned media altered phagocytosis. N) iMGL stained for the microglial marker IBA1 (green); scale bar = 50 µm. O) iMGL branch length did not vary by treatment with serum amyloids or iMP‐conditioned media. Data are presented as mean (±SEM) values; *n* = 3‐5 independent wells/group for J, K, and M. *n* = 118–219 cells/group for O. * *p* < 0.05, ** *p* < 0.01, *** *p* < 0.001, **** *p* < 0.0001, ns = not significant; one‐way ANOVA with Tukey's multiple comparisons test correction.

Because iMPs were not found in the brain, we hypothesized that their cognitive benefits could be due to alterations in the plasma proteome and thus, performed a discovery proteomic analysis of plasma. Peptide fragments were analyzed, and a total of 485 proteins were identified. Using MAP DIA software, relative amounts of peptides and proteins within samples were determined and log2 fold changes (log2FC) between groups were calculated. Several proteins were differentially expressed in aging versus young mice (Figure S8A, Supporting Information), with 75 upregulated and 34 downregulated at imputed FDR<0.05 and FC≥1.5. Notably, almost half of the differentially expressed proteins were linked to AD, while 30% were implicated in inflammation, and several other proteins were known to be altered in aging or play a role in cognition (Table S1, Supporting Information). Comparing iMP‐treated mice to young mice revealed 63 upregulated and 32 downregulated proteins (Figure S8B, Supporting Information). Haptoglobin (Hp), the top upregulated protein (increased 5.4‐fold in aging versus young mice), is known to increase dramatically in aging mouse plasma,^[^ ^42^, ^45^ ^]^ and showed a less pronounced 4.2‐fold increase in iMP‐treated versus young mice. Finally, comparing aging mice treated with iMPs versus vehicle revealed 3 upregulated (adiponectin, MTUS1, and GAPDH) and 4 downregulated (SAA2, Ppp1r21, SAP, and Arhgap30) proteins (**Figure** **5**A). Adiponectin and GAPDH were also significantly decreased in aging mice as compared to young mice, while SAP and Arhgap30 were significantly increased in aging mice (Figure S8A, Supporting Information), demonstrating that iMP treatment reversed these age‐associated changes. Interestingly, the majority of iMP‐treated mice had protein expression that was more similar to young than to aging mice (Figure 5B).

We also examined plasma levels of two well‐established pro‐aging factors; B2M^[^ ^46^ ^]^ and VCAM1.^[^ ^47^ ^]^ B2M, detected in discovery proteomics, did not differ across groups, whereas VCAM1, assayed via parallel reaction monitoring (PRM), was significantly increased in aging, with a trend toward decreased expression in iMP‐treated mice (Figure S8F,G, Supporting Information).

To identify the biological processes differentially regulated by aging, gene ontology (GO) analyses were performed for proteomic data (Table S2, Supporting Information), with significant biological process GO terms associated with the 75 upregulated genes in aging versus young mice shown in Figure S8C (Supporting Information). Among the top overrepresented GO terms were several related to opsonization, synapse pruning, fibrinolysis, and complement activation, which were all enriched by 70‐fold or greater. A reactome pathway analysis also showed enrichment for several complement system‐related terms (Figure S8D, Supporting Information). When examining the 34 proteins downregulated in aging mice, two cellular compartment GO terms associated with synaptic function were significantly enriched (Table S2, Supporting Information). Interestingly, complement activation leads to synaptic pruning,^[^ ^48^ ^]^ and synaptic proteins in the blood have been identified as early biomarkers for AD diagnosis 5–7 years later.^[^ ^49^ ^]^ Collectively, these analyses point to a significant activation of the complement system in aging plasma as well as downstream synaptic pruning.

GO analysis on the 63 proteins upregulated in iMP‐treated versus young mice showed enrichment for several similar terms as found in the comparison between aging and young mice; however, no GO terms related to the complement system were found within the top 20 enriched terms (Figure S8E, Table S2; Supporting Information). In fact, the only enriched complement‐related term was “complement activation (GO:0006956)”, which showed only an 8.93‐fold enrichment in iMP‐treated versus young mice, as opposed to the top complement‐related GO term (“complement activation, alternative pathway (GO:0006957)”) enriched 77.9‐fold in aging versus young mice.

Two proteins of particular interest were serum amyloid A2 (SAA2) and serum amyloid P component (*Apcs* or SAP), as these have been implicated in AD^[^ ^50^, ^51^, ^52^ ^]^ and aging, and the aging‐associated increase in their levels was significantly decreased in the plasma of iMP‐treated mice (Figure 5C). To confirm changes in these proteins, an assay using targeted liquid chromatography mass spectrometry via PRM was developed, targeting SAA2 and SAP. Results of this assay confirmed those of discovery proteomics (Figure 5D).

To examine the potential functional importance of these two serum amyloid proteins, we assessed the extent to which they correlated with other key outcomes altered by iMP treatment. Levels of SAP showed trends toward significant correlations with novel object location performance (R = −0.231; *p* = 0.07) and the mossy cell marker, GluR2/3 (R = −0.262; *p* = 0.05), while SAA2 levels significantly correlated with novel object location performance (R = −0.345; *p* = 0.01), but not with GluR2/3 (R = −0.193; *p* = 0.12; Figure 5E–H). Thus, there appears to be an overall trend, where animals with higher levels of serum amyloid proteins perform worse at the novel object location task and have lower levels of mossy cells.

SAA2 can readily cross the intact blood–brain barrier (BBB),^[^ ^53^, ^54^ ^]^ and SAP has been shown to enter the brain parenchyma after peripheral LPS administration.^[^ ^55^ ^]^ Thus, elevated levels of these proteins in plasma could act directly on cells in the brain, especially in conditions of increased BBB permeability, which has been observed even in healthy aging,^[^ ^56^ ^]^ where it is associated with increased neuroinflammation.^[^ ^57^ ^]^ To test the effects of serum amyloids directly on microglia, we treated iPSC‐derived microglia (iMGL) with SAP and SAA2. The number of healthy, DAPI+ iMGL was significantly decreased after 24 h exposure to SAP and SAA2 (Figure 5I‐J). Because the beneficial effects of iMPs occur in the absence of their entering the brain, conditioned media from iMP cultures was used to test whether iMP‐secreted products could protect against the adverse effects of serum amyloid treatments. Notably, iMP‐conditioned media significantly increased cell viability (Figure 5J) and protected against late‐stage apoptosis induced by SAP and SAA2, as measured by CC3 staining (Figure 5K). Serum amyloids did not alter iMGL phagocytosis or branch length (Figure 5L–O). iMP‐conditioned media also protected against camptothecin (CAMP), a potent apoptotic insult, by reducing the number of cells in late‐stage apoptosis (Figure S8I, Supporting Information). While CAMP treatment significantly reduced iMGL branch length, iMGL treated with CAMP in iMP‐conditioned media were not significantly different from vehicle‐treated iMGL (Figure S8K, Supporting Information). Finally, iMGL were treated with bovine serum albumin (BSA) as a negative control, and results demonstrated that BSA did not have significant effects on any measure of microglial health (Figure S8H–K, Supporting Information).

In order to begin assessing potential beneficial factors secreted by iMPs, discovery proteomic analysis was conducted on conditioned media from iMP cultures. Basal RPMI media was also analyzed, and any proteins found in RPMI were subtracted from iMP‐conditioned media samples. The top 50 most abundant proteins are shown in Figure S8L (Supporting Information). GO analyses on these proteins reveal terms related to amyloid, the immune system, and the complement system (Figure S8M,N, Supporting Information), which overlap with some of the terms found in analyses on plasma from iMP‐treated animals. Of particular interest is the finding that, among the top 50 proteins, there are several that were differentially expressed in plasma from experimental animals. This includes GAPDH, which is significantly decreased in plasma of aging mice, but significantly increased after iMP treatment. GSN is decreased in aging, while C1QC and C1QB are both increased; notably, when compared to young mice, these proteins are altered to a lesser extent in iMP‐ than in vehicle‐treated aging mice. Thus, these particular proteins may have a mechanistic role in the beneficial effects of iMPs. Additionally, a literature search revealed that the vast majority (42 out of the top 50 proteins) secreted by iMPs have been implicated in aging and/or AD, while others have been shown to have a role either in inflammation or in cognition (Table S3, Supporting Information).

In summary, we find numerous aging‐associated plasma proteome changes, several of which are attenuated by iMP treatment, including increased levels of SAP and SAA2. Importantly, serum amyloids directly decrease microglial viability in vitro, while iMP‐conditioned media reverses this effect. Because numerous studies have shown a role for serum amyloids in AD, we next tested the therapeutic potential of iMPs in a mouse model of AD.

### 2.6 iMPs Improve Cognition and Microglial Health in 5xFAD Mice

**Figure 6:**
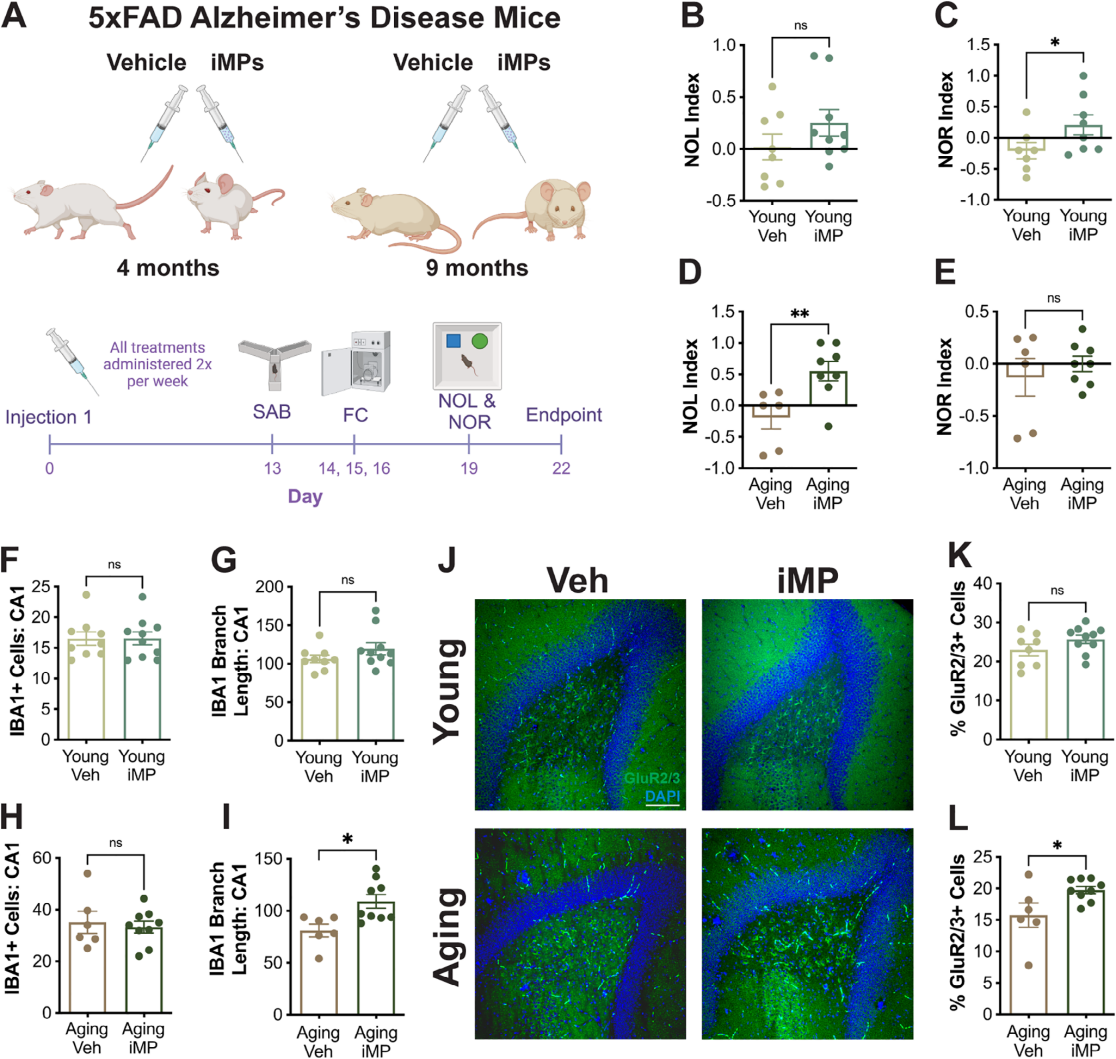
iMPs improve cognition and microglial health in 5xFAD mice. A) Schematic showing 5xFAD mice were treated with either vehicle (saline) or iMPs (500,000 cells/injection) beginning at either 4‐ or 9‐months‐of‐age. Treatments were administered twice weekly, and behavior was assessed at the indicated timepoints. B‐E) iMP treatment improved performance in the novel object recognition (NOR) task in young 5xFAD mice (C), and in the novel object location (NOL) task in aging 5xFAD mice (D). F‐G) Microglial numbers and branch length were not different between vehicle‐ and iMP‐treated young 5xFAD mice. H‐I) iMP treatment had no effect on microglial number but significantly increased microglial branch length in aging 5xFAD mice. J) Representative images of immunohistochemical staining for the mossy cell marker GluR2/3 in hippocampal dentate gyrus. Scale bar = 100 µm. K‐L) Compared to vehicle‐treated mice, the percent of GluR2/3+ cells was significantly increased in iMP‐treated aging, but not young, 5xFAD mice. Data are presented as mean (±SEM) values; *n* = 6‐10/group. * *p* < 0.05, ** *p* < 0.01, ns = not significant; unpaired *t*‐test.

Based on our findings that iMPs significantly improve cognition and neural health in aging mice and reverse age‐associated increases in serum amyloid proteins, we hypothesized that iMPs might be beneficial in a mouse model of AD. iMPs were administered to male 5xFAD mice beginning either at 4‐months‐of‐age, when amyloid‐β (Aβ) accumulation is just beginning, or at 9‐months‐of‐age, when this mouse model already has significant AD‐like pathology (**Figure** **6**A). This enabled the evaluation of iMPs as both a preventative and an intervention treatment in the context of AD.

Both vehicle‐ and iMP‐treated young 5xFAD mice gained weight over the course of injections, and there was no significant weight difference between treatment groups in either young or aging 5xFAD mice (Figure S9A,B, Supporting Information). iMP treatment did not alter arm entries or spontaneous alternation behavior in either young or aging 5xFAD mice (Figure S9C,D, Supporting Information). While there were no significant differences in contextual fear conditioning at either age, young 5xFAD mice specifically showed an increase in freezing in the cued fear conditioning task in response to iMP treatment (Figure S9E,F, Supporting Information). It has previously been reported that 5xFAD mice show early deficits in non‐hippocampus‐dependent tasks like novel object recognition,^[^ ^58^, ^59^, ^60^ ^]^ while deficits in hippocampus‐dependent tasks like novel object location emerge later.^[^ ^61^, ^62^, ^63^ ^]^ Remarkably, iMP treatment specifically increased performance in the recognition task in young 5xFAD mice (Figure 6B‐C), while in aging 5xFAD mice, iMPs specifically improved novel object location performance (Figure 6D,E).

While total microglial numbers were not changed after iMP treatment in either hippocampal CA1 or CA3 at either age, iMP‐treated aging 5xFAD mice had increased microglial branch length relative to their vehicle‐treated counterparts (Figure 6F–I; Figure S9G,H, Supporting Information). AD‐like pathology was assessed by staining for and quantifying the percent area occupied by Aβ plaques across hippocampal subregions. iMP treatment did not alter Aβ pathology in CA1, CA3, or subiculum at either age (Figure S9I,J, Supporting Information). While young 5xFAD mice did not yet have Aβ plaques in DG, the increased level with aging was not altered by iMP treatment (Figure S9J, Supporting Information). Soluble forms of Aβ40 and Aβ42 were measured by extracting and analyzing both TBS‐soluble and Triton X detergent‐soluble fractions from the cortex and hippocampus of young and aging 5xFAD mice. There was a trend toward a significant decrease in the ratio of Triton X‐soluble Aβ42 to Aβ40 (*p* = 0.06) in young iMP‐treated mice (Figure S9K, Supporting Information), suggesting a shift toward the less toxic form of Aβ. However, overall, there were no significant differences between vehicle‐ and iMP‐treated mice at either age for any fraction of Aβ40 or Aβ42 (Figure S9K,L, Supporting Information).

Because iMP treatment was associated with changes in DG mossy cells in aging NSG mice, we also stained for and quantified GluR2/3+ cells in 5xFAD mice. While the percent of GluR2/3+ cells did not differ in young mice, aging 5xFAD mice treated with iMPs showed a significant increase in this population compared to vehicle‐treated aging 5xFAD mice (Figure 6J–L). As in aging NSG mice, this increase in GluR2/3+ cells was not due to increased neurogenesis, as neither young nor aging mice showed treatment effects on DCX+ cells (Figure S9M, Supporting Information). Furthermore, iMP treatment did not appear to alter general cell proliferation, as demonstrated by quantifying Ki‐67+ cells in the DG of young 5xFAD mice (Figure S9N, Supporting Information).

Collectively, these results demonstrate that iMP treatment improves recognition and location memory in young and aging 5xFAD mice, respectively, and increases microglial branch length and mossy cells in aging 5xFAD mice, in the absence of significant changes in Aβ pathology.

## 3 Discussion and Conclusion

Previous work demonstrates that young plasma or bone marrow has regenerative effects in aged animals, improving cognition and neural health.^[^ ^8^, ^9^ ^]^ However, as these strategies have drawbacks, the current study determined whether iPSC‐derived mononuclear phagocytes can provide regenerative effects. Aging mononuclear phagocytes show altered cytokine production,^[^ ^64^, ^65^ ^]^ decreased phagocytosis,^[^ ^66^ ^]^ and an inability to restore homeostatic function after an inflammatory response.^[^ ^11^ ^]^ However, iPSCs from elderly patients can be used to generate rejuvenated mononuclear phagocytes from an autologous tissue source.

A relatively easy and short differentiation protocol generated iMPs, which can be harvested over months. iMP delivery was based on a regimen using young plasma that protected against aging‐induced impairments in contextual fear conditioning and spatial working memory.^[^ ^7^, ^8^ ^]^ We did not observe aging‐associated deficits in fear conditioning in our model, but did recapitulate reports showing both hippocampus‐dependent spatial working and short‐term memory decline with age^[^ ^17^, ^19^, ^20^ ^]^ in the NSG model. In both instances, iMP‐treated mice were not significantly different from young mice. Supporting the central role of the hippocampus in our aging model, cortical‐dependent recognition memory was preserved.^[^ ^19^, ^20^ ^]^

In some assays, like the novel object location task, microglial LAMP1 volume, and mossy cell gene expression, 3 weeks of iMP treatment completely reversed effects of aging, leading to a significant difference between iMP‐ and vehicle‐treated aging groups. In other assays, like spontaneous alternation and several other histological outcomes, there was no longer a significant decline in iMP‐treated animals when compared to young animals, but there was no significant difference between iMP‐ and vehicle‐treated aging mice. This suggests that iMPs had a partial regenerative effect but did not reverse all aging‐associated changes in NSG mice. Current studies are determining if longer or higher dose treatments may have wider effects, and future studies are needed to determine whether the beneficial effects of iMPs extend to memory retention longer than 30 min, as tested in novel object location, as well as to other aspects of memory and cognition. While the spontaneous alternation task requires medial prefrontal cortex function,^[^ ^67^, ^68^ ^]^ additional behavioral tests that focus on executive function, like the attentional set shifting task or operant conditioning paradigms, should be included in future studies. However, the findings of treated aging mice being similar to young mice across a range of outcome measures are very promising.

While behavior in the wildtype BALB/c model was more variable and did not reach statistical significance, the aging vehicle‐treated mice showed a greater drop in mean performance on novel object location, when compared to young mice, than did aging mice treated with iMPs. Further, microglial branch length and complexity were significantly reduced in aging BALB/c mice, as they were in aging NSG mice. In both models, iMP‐treated mice were not statistically different from young mice, and in fact, in the wildtype BALB/c model, aging iMP‐treated mice had significantly greater branch length and complexity than did aging vehicle‐treated mice. These effects on microglial outcomes were maintained after 10 weeks of treatment in the BALB/c mice, suggesting that extended iMP treatments retain efficacy, at least up to this timepoint.

Remarkably, hippocampal snRNA‐seq analysis identified subclusters of mossy cells that were absent in aging mice but restored with iMP treatment. Based on the genes highly enriched in these subpopulations, these cells may be responsible for cognitive benefits of iMP treatment. For example, *Kcnma1* and *Kcnc2* encode for potassium channel subunits that regulate neuronal excitability and mutations are associated with epilepsy and intellectual disability.^[^ ^69^, ^70^ ^]^ Genes including *Vav3*,^[^ ^71^, ^72^ ^]^ *Rnf220*,^[^ ^73^ ^]^ *Hdac9*,^[^ ^74^ ^]^ and *Shisa9* ^[^ ^75^, ^76^ ^]^ are involved in synapse formation and plasticity, while others are critical in cognition and long‐term potentiation. For example, *Syt9* correlates with verbal short‐term memory in cognitively normal older adults;^[^ ^77^ ^]^ *Lef1* and *Tcf7l2* regulate WNT signaling and are required for granule cell development^[^ ^78^ ^]^ and ventricular zone neurogenesis,^[^ ^79^ ^]^ respectively, while *Cit* regulates mitosis in the ventricular zone.^[^ ^80^ ^]^ Polymorphisms in *Tcf7l2* have been associated with cognitive deficits in cardiovascular disease patients.^[^ ^81^ ^]^ Meanwhile, *Robo1*, an essential axon guidance receptor,^[^ ^82^ ^]^ is involved in neurogenesis,^[^ ^83^ ^]^ and interestingly, *Robo1* knock‐out was recently shown to cause an accelerated aging phenotype and shortened lifespan.^[^ ^84^ ^]^ Finally, *Fgf13* has been shown to mediate the age‐associated decrease in DG neurogenesis.^[^ ^85^ ^]^ Notably, these subcluster‐enriched genes showed increased expression throughout all mossy cells in iMP‐treated versus aging mice, such that iMPs not only restored specific subclusters but were beneficial to the entire mossy cell population. Mossy cells are critical for sensing and forming memories of novel environments,^[^ ^37^, ^38^ ^]^ which is essential in tasks like novel object location. Thus, our findings that mossy cells have the greatest number of differentially expressed genes between aging and iMP‐treated mice, have significantly increased expression of genes involved in synaptic plasticity and long‐term potentiation after iMP treatment, and have a significantly younger transcriptional age in iMP‐treated mice, collectively aligns with the significant improvement in the behavioral task that relies heavily on this neuronal subtype, and the protective effects of iMPs on mossy cells may be directly related to improvements observed in this task.

The number of DCX‐positive cells is unaltered by iMP treatment, in both NSG and 5xFAD mice, suggesting that iMPs’ effects are not due to global neurogenesis. While the number of cells expressing calretinin is not changed, this may be due to the previously reported regional specificity of mossy cells, with not all cells expressing calretinin,^[^ ^86^, ^87^ ^]^ and with calretinin also marking a population of inhibitory interneurons.^[^ ^41^ ^]^ In contrast, we did find a significant aging‐associated decrease in cells expressing the mossy cell‐specific marker, GluR2/3, which was not significant in iMP‐treated animals. In addition to preventing decreases in global mossy cell numbers, gene expression within mossy cell subclusters suggests that iMP treatment leads to changes in synaptic plasticity. Moreover, numerous changes in microglial immunoreactivity and morphology were observed, and a number of cell types including microglia and mossy cells showed a reversal of transcriptomic aging after iMP treatment. Hence, iMPs may have dual actions: improving microglial health while enhancing synaptic plasticity in mossy cells. As with iMP treatment, we previously found that the cognition‐enhancing effects of young bone marrow transplants also occur in the absence of increased neurogenesis.^[^ ^9^ ^]^ Additionally, our data show that iMPs significantly improve the health of mossy cells, which are critical for the formation of memories about novel environments. Thus, the effects of iMPs on novel object location performance are likely due to their effects on mossy cells, rather than due to increasing neurogenesis.

Proteomic analysis of plasma identified several pathways that may mediate the beneficial effects of iMPs. Of note, serum amyloid and complement pathways were increased in aging and decreased with iMP treatment. iMP treatment altered SAP and SAA2, both serum amyloid proteins implicated in AD^[^ ^50^, ^51^, ^52^ ^]^ and aging.^[^ ^88^, ^89^ ^]^ SAP is a drug target in anti‐amyloid therapies,^[^ ^90^, ^91^ ^]^ as it accelerates and stabilizes the toxic amyloid‐β plaques that accumulate in AD,^[^ ^52^, ^92^ ^]^ and there is a strong association between cortical levels of SAP and dementia, independent of amyloid pathology.^[^ ^93^ ^]^ In mice, SAP deletion results in delayed amyloid deposition.^[^ ^94^ ^]^ Likewise, SAA2 is secreted during acute inflammation and implicated in several chronic inflammatory diseases,^[^ ^95^ ^]^ as well as in neurodegenerative disorders. For example, SAA levels correlate with lesion size and cortical volume in patients with multiple sclerosis,^[^ ^96^ ^]^ and SAA2 has been proposed as a biomarker for traumatic brain injury outcomes as it correlates with markers of neuroinflammation and neurodegeneration.^[^ ^97^ ^]^ In particular, hepatic production of SAA increases following a traumatic brain injury, resulting in SAA expression in microglia and astrocytes.^[^ ^98^ ^]^

SAA2 is increased in post‐mortem AD‐affected brain regions,^[^ ^50^ ^]^ as well as in serum of AD patients,^[^ ^99^ ^]^ and is correlated with cognitive decline in vascular dementia.^[^ ^51^ ^]^ Moreover, there is local SAA production in brains of AD patients, but not in those with other neurodegenerative diseases.^[^ ^100^ ^]^ In mice, the combination of SAA expression and an inflammatory insult is sufficient to trigger Aβ plaque deposition,^[^ ^101^ ^]^ while SAA‐deficient mice have better outcomes following middle cerebral artery occlusion.^[^ ^102^ ^]^ Notably, SAA2 readily crosses the BBB,^[^ ^53^ ^]^ while SAP has been shown to cross under inflammatory conditions.^[^ ^55^ ^]^ Hence, not only do serum amyloid levels increase with aging, but the combination of increased inflammation and increased BBB permeability may enable accumulation of these proteins in the brain, where they can then increase microglial activation. We find a significant decrease in serum amyloid levels following iMP treatment, and this may be responsible for improvements in microglial and neuronal health observed in iMP‐treated animals.

Serum amyloids have also been shown to be toxic,^[^ ^102^, ^103^, ^104^ ^]^ with intrahippocampal injections of SAP inducing neuronal apoptosis.^[^ ^105^ ^]^ Our in vitro findings recapitulate this, as iMGL treated with SAP and SAA2 show increases in CC3 immunoreactivity. Because iMPs do not cross the BBB, their effects are likely mediated by their secretion of regenerative factors. To model this, we used conditioned media from iMP cultures, which significantly reduced iMGL apoptosis induced by either serum amyloids or CAMP. While serum amyloids did not affect iMGL branch length, CAMP‐treated cells did show significantly reduced branch length; however, this was not significant in cells treated with CAMP in the presence of iMP‐conditioned media. The fact that iMP treatment increases microglial branch length in vivo but not in vitro suggests that cell culture models only partially recapitulate the effects of iMPs or that iMPs are acting through multiple mechanisms to achieve their beneficial effects on cognition and neural health. That is, while iMP‐conditioned media contains factors secreted by iMPs, it does not recapitulate what iMPs may be taking up or what effects they may have on peripheral organs that are then translated to the brain. Recent studies have shown that simply diluting aged plasma has similar rejuvenating effects to young plasma^[^ ^106^ ^]^ and is beneficial in AD patients,^[^ ^107^ ^]^ and while iMP treatment may also have this effect, our in vitro results demonstrate that this is not the only mechanism through which the cells are acting.

While studies to elucidate the mechanism of action of iMPs are needed, our data highlight several potential pathways. Although some monocytes can cross the blood–brain barrier and survive for at least 21 days when administered via carotid artery infusion, this is reduced with tail vein administration.^[^ ^108^ ^]^ Moreover, our previous mouse GFP+ bone marrow transplants showed no evidence of entering the brain.^[^ ^9^ ^]^ It is very unlikely that iMPs enter the brain to act directly on cells, as these cells were not detected by either PCR or immunohistochemical staining for a human‐specific antibody, and hippocampal snRNA‐seq did not show a separate human cell population in iMP‐treated mice. This lack of cell penetrance through an intact blood–brain barrier is predicted by prior studies.^[^ ^109^, ^110^, ^111^ ^]^ However, iMP treatment significantly altered expression of several proteins in aging mouse plasma, indicating that systemic effects of iMPs may be mediated through signaling in the periphery that is then translated to the brain. Interestingly, not only are the majority of the proteins secreted by iMPs implicated in aging and/or AD, but several have been identified as potential therapeutic targets.^[^ ^112^, ^113^, ^114^ ^]^ Future studies will test the extent to which these factors, or combinations of them, could be used to obtain similar benefits as with iMPs. However, it may be the case that the full milieu of iMP products is required for their effects.

Microglia are a likely target of iMP action, as they respond to peripheral cues, including sepsis,^[^ ^115^ ^]^ bacterial infection,^[^ ^116^ ^]^ sex steroid hormones,^[^ ^117^ ^]^ and even high fat diet.^[^ ^118^ ^]^ In fact, higher levels of peripheral cytokines associate with increased risk of AD in cognitively normal older adults^[^ ^119^ ^]^ and correlate with rates of cognitive decline in AD patients.^[^ ^120^ ^]^ Peripherally administered LPS decreases microglial clearance of Aβ in an AD mouse model,^[^ ^121^ ^]^ even under conditions of low‐grade peripheral inflammation.^[^ ^122^ ^]^ Moreover, it was recently shown that genetically modulating peripheral macrophages decreased microglial activation, which increased survival in a mouse model of amyotrophic lateral sclerosis,^[^ ^123^ ^]^ demonstrating the feasibility of altering peripheral mononuclear phagocytes to modify microglial function and neurodegenerative disease outcomes. We find that iMPs reverse several aging‐associated changes in microglia; thus, irrespective of whether iMPs act directly in the brain or have peripheral effects that are then translated to the brain, results indicate that microglia are a key target of iMPs.

In previous studies testing young blood products, female NSG were excluded from behavioral analyses due to increased variability and “persistent hyperactivity”.^[^ ^8^ ^]^ Despite this precedent in the literature, we felt it would be informative to also assess female mice. Interestingly, we observed several measures on which males and females are similar as well as some measures with sex differences. While the effects of aging and iMP treatment on behavioral outcomes did not reach statistical significance in female NSG mice, the general trend for novel object location performance was similar to males. Further, while VGLUT1 levels did not differ across groups in females, microglial branch length was significantly decreased in aging, but increased after iMP treatment. Sex differences in aging and neural health and cognition are well documented,^[^ ^124^ ^]^ and, while the possible reasons are not fully elucidated, these may be due to sex steroid hormones.^[^ ^125^ ^]^ Future studies will further evaluate effects of iMPs in aging female mouse models, and especially in transgenic female AD mice, as there are important sex differences in this disease,^[^ ^126^ ^]^ which are recapitulated in the 5xFAD mouse.^[^ ^127^, ^128^ ^]^ Female and male monocytes are characteristically different^[^ ^66^ ^]^ and sex differences exist in response to cell or organ transplants.^[^ ^129^ ^]^ Specifically, the sex of donor cells does not appear to affect outcomes in male recipients, but adverse results have occurred in females receiving transplants of male kidney,^[^ ^130^ ^]^ lung,^[^ ^131^ ^]^ and heart.^[^ ^132^ ^]^ iMPs can be generated as an autologous cell therapy, thus preventing adverse effects of sex mismatch.

Our findings in 5xFAD mice demonstrate that iMPs are beneficial not only in the context of aging but also in AD. iMPs improved performance on the novel object recognition task in young 5xFAD mice and in the novel object location task in aging 5xFAD mice; changes that align with previous reports of early deficits in recognition memory and later deficits in spatial memory.^[^ ^58^, ^59^, ^60^, ^61^, ^62^, ^63^ ^]^ Similar to effects observed in aging models, iMPs increased microglial branch length and GluR2/3+ mossy cells. This was found specifically in aging 5xFAD mice, as young mice may not yet have microglial changes and loss of mossy cells. iMPs did not decrease hippocampal Aβ plaque load or levels of soluble Aβ; however, amyloid levels and cognitive performance show poor correlations across a number of studies.^[^ ^133^ ^]^ Like iMPs, antibody therapies against Aβ have been shown in mouse models to improve cognition, without altering brain levels of amyloid.^[^ ^134^, ^135^ ^]^ Conversely, while the currently approved anti‐amyloid therapies lead to significant reductions in patients’ amyloid levels, they show only small effects on cognition and relatively short‐term benefits in terms of delaying disease progression.^[^ ^136^ ^]^ Because of this, there is increasing recognition of the need for non‐amyloid‐targeting therapeutics,^[^ ^137^ ^]^ especially ones targeting neuroinflammation.^[^ ^138^ ^]^ Moreover, the finding that iMPs improved various outcomes both at an early and later stage of AD pathology highlights their potential as a prevention as well as an intervention. Ultimately, iMPs could be administered alongside other therapies that act on different mechanisms, and this combined therapeutic approach may augment meaningful clinical impact.

While many cell therapies for neurodegeneration aim to replace populations of lost cells, iMPs would provide a more general neuroprotective strategy, aimed at maintaining the health of host cells. Other circulatory immune cells may also be beneficial and could be tested in a future study. Indeed, a number of studies have demonstrated beneficial effects of mesenchymal stem cells (MSCs) on cognitive decline and AD.^[^ ^139^, ^140^, ^141^, ^142^ ^]^ While some clinical trials using MSCs are ongoing, several have not shown efficacy in humans.^[^ ^143^ ^]^ Not only may iMPs be acting via a different mechanism, as we did not observe the increased neurogenesis that has been reported after MSC delivery,^[^ ^139^, ^140^ ^]^ but also, iMPs are derived from iPSCs, unlike the adult MSCs used previously. Thus, it will be of interest to determine whether effects of iMPs are due to their generation from reprogrammed and thus, “youthful” cells, or whether the same effects could be obtained using “older” cells. Moreover, the fact that iMPs are derived from iPSCs allows for a scalable and personalized cell therapy.

Improvements occurred after only a 21‐day intervention, suggesting that iMPs reversed rather than prevented cognitive decline, as such drastic aging and cognitive decline are unlikely to occur over this short time period. However, additional studies need to determine whether iMPs reverse or prevent age‐related decline, as this has implications for optimal delivery time. One limitation of the current study is that we tested the effects of iMPs in mice aged 11–12 months. While NSG mice have been reported to have an accelerated aging phenotype,^[^ ^8^ ^]^ future studies will need to determine whether iMPs are beneficial at more advanced ages. Future dose ranging and time course studies will need to evaluate the optimal dose of cells, as well as how rapidly behavioral changes occur and how long they are sustained after treatment. Full necropsies were performed on all animals enrolled in these studies, including 48 mice that received iMP treatment. We did not observe tumors or gross changes in any organ of treated mice, or any other observable differences between vehicle‐ and iMP‐treated animals, suggesting that iMPs are safe and well‐tolerated, though future studies will test the long‐term safety of these cells using Nude rats. Unlike other treatments, iPSC‐derived mononuclear phagocytes could be used as an individualized cell therapy with unlimited availability and without risks of graft versus host disease or immune rejection. Alternatively, iMPs could be derived from hypo‐immune iPSCs that are genetically engineered to evade the host immune system,^[^ ^144^ ^]^ thereby providing a widely applicable therapeutic that could avoid immune suppression drugs or the time and expense of autologous cell line production. In summary, short‐term iMP treatment has significant beneficial effects on cognition and on many facets of neural health in multiple rodent models of aging and in both young and aging 5xFAD mice, and thus, iMPs are a promising therapeutic candidate to address age‐ and AD‐related cognitive decline.

## 4 Experimental Section

### Experimental Animals

Male and female NOD scid gamma (NSG) mice were purchased from Jackson Laboratories (strain # 0 05557) at 2.5 months of age and either aged to 11–12 months or enrolled in studies after a 2‐week acclimation period to the vivarium facilities at Cedars‐Sinai Medical Center. These mice are on the NOD strain background, along with a severe combined immunodeficiency (*scid*) mutation in *Prkdc* resulting in a B and T cell deficiency that essentially eliminates adaptive immunity, as well as knockout of the interleukin 2 receptor gamma chain (*IL2rg^null^*) which inhibits cytokine signaling thereby leading to a deficiency in natural killer cells. This extreme immunodeficiency permits the survival of transplanted human cells.^[^ ^16^ ^]^ BALB/c mice were purchased from Taconic Biosciences and after at least 2 weeks of acclimation, were enrolled in studies at 3‐ or at 11‐13‐months‐of‐age. 5xFAD mice were purchased from the MMRRC (stock # 034840‐JAX) and treated with vehicle or iMPs. All 5xFAD mice were purchased at weaning in one order and were then aged to either 4‐ or 9‐months‐of‐age before beginning treatments. BALB/c and 5xFAD mice received IP injections of 15 mg/kg cyclosporin A every 24 h beginning 3d prior to their first iMP treatment, after which they received 200 mg/L cyclosporin A in their drinking water for the remainder of the experiment. Mice were mechanically restrained and administered either sterile saline (vehicle) or iPSC‐derived mononuclear phagocytes (iMPs) at a dose of 500 000 cells in 100 µL via tail vein injection. This dose was determined based on the total number of monocytes typically present in the circulation of mice, which ranges from about 88 000–440 000. Thus, a dose slightly above this upper threshold is used in order to ensure that iMPs would reliably outnumber host monocytes. Littermates of the same age were randomly assigned to experimental conditions, and treatments were administered every third day for a total of 8 injections, based on an established protocol.^[^ ^8^ ^]^ Except for BALB/c mice and as noted in the text, all post‐mortem analyses were performed on tissues collected after 21 days of treatment.

At the conclusion of the experimental period, 24 h after receiving their last treatment, mice were anesthetized with inhalant isoflurane and transcardially perfused with ice‐cold 0.1 M phosphate buffered saline (PBS). Brains were rapidly removed, and one hemi‐brain was post‐fixed by immersion in 4% paraformaldehyde (PFA)/ 0.1 M PBS for 48 h at 4 °C, before storage in 0.1 M PBS/0.03% NaN_3_. The remaining hemi‐brain's hippocampus was dissected and snap frozen for subsequent use in single‐nucleus RNA sequencing (snRNA‐seq). Blood was collected via cardiac puncture into EDTA‐coated tubes and centrifuged to separate plasma, which was stored in aliquots at −80 °C, before being processed for proteomic analyses. All animals were group‐housed (up to 5 animals per cage) under a 12 h light/dark cycle with ad libitum access to food and water. All animal procedures were conducted under protocols approved by the Cedars‐Sinai Institutional Animal Use and Care Committee (IACUC #008174) and in accordance with National Institute of Health standards.

### Cell Lines

Induced pluripotent stem cell (iPSC) lines were generated at the iPSC Core at Cedars‐Sinai Medical Center. Parent cells were transfected with non‐integrating oriP/EBNA1 plasmids that rely on episomal expression of reprogramming factors. All cell lines and protocols were approved for use under the Institutional Review Board (IRB) and Stem Cell Research Oversight Committee (SCRO) protocols #21 505 and #32 834.

### iMP Differentiation

Reprogramming was conducted by non‐integrating methods. All iPSC lines were fully reprogrammed, as demonstrated by staining for alkaline phosphatase and other pluripotency markers, and passed the “PluriTest”, as shown by low gene expression of “novelty” markers and high expression of “pluripotency” markers. Southern blotting and genomic PCR analyses confirmed the absence of plasmid gene expression after several passages, demonstrating that reprogramming plasmids were not integrated.^[^ ^13^ ^]^

iPSCs were differentiated into iMPs following a previously described protocol.^[^ ^14^, ^15^ ^]^ iPSCs were grown in 1 mg/6‐well plate Matrigel (#354 230, Corning) in mTeSR (#5850, StemCell Technologies Inc.) at 37 °C in 5% O_2_ until they reached 80–90% confluency. Lines were passaged at least 3 times using Versene solution (#15 040 066, Thermo Fisher Scientific) before being plated for microglial differentiation. iPSCs were then passaged using a StemPro EZPassage Disposable Stem Cell Passaging Tool (#23 181 010, Life Technologies), and plated in a 100 mm cell culture dish on Matrigel in mTeSR. Cells were maintained over the next 2–4 days, with removal of any cells that were differentiating, too close to other colonies, or oddly shaped. Once each dish had roughly 60–80 colonies measuring 1.0 mm in diameter on average, differentiation was initiated using stage 1 media, consisting of 80 ng/ml bone morphogenetic protein 4 (BMP4; #314‐BP‐010, R&D Systems) in mTeSR. Media was changed daily for 4 days, at which point cells were switched to stage 2 media, consisting of 25 ng/ml basal fibroblast growth factor (bFGF; #01‐106, Millipore Sigma), 100 ng/mL stem cell factor (SCF; #255‐SC‐010, R&D Systems) and 80 ng/mL vascular endothelial growth factor (VEGF; #293‐VE‐010, R&D Systems) in StemPro‐34 serum free medium (#10 639 011, Gibco). Two days later, cells were switched to stage 3 media, consisting of StemPro‐34 supplemented with 50 ng/ml SCF, 50 ng/ml interleukin‐3 (IL‐3; #203‐IL‐010, R&D Systems), 5 ng/ml thrombopoietin (TPO; #288‐TP‐005, R&D Systems), 50 ng/mL macrophage colony stimulating factor (M‐CSF; #216‐MC‐010, R&D Systems), and 50 ng/mL fms‐related tyrosine kinase 3 ligand (Flt3; #308‐FK‐005, R&D Systems). Media was changed 4 days later and replaced with fresh stage 3 media until day 12–14, when cells were switched to stage 4 media, consisting of 50 ng/mL M‐CSF, 50 ng/mL Flt3 ligand, and 25 ng/mL granulocyte‐macrophage colony stimulating factor (GM‐CSF; #215‐GM‐010, R&D Systems) in StemPro‐34 medium. Four days later, the floating cells in the supernatant were collected, pelleted, resuspended in fresh stage 4 media, and returned to their respective plate, with even distribution among the wells. These collection feeds were performed every 4 days, with floating cells collected for treatments rather than being resuspended, during every other feed. When collected for treatments, cells were pelleted, counted, and then resuspended in sterile saline at a concentration of 500 000 cells per 100 µL. An additional control group receiving heat‐inactivated (referred to as “dead‐iMPs”) was included in experiments using the wildtype BALB/c mice. For this, iMPs were collected as described above and placed in a dry bath at 95 °C for 15 min before being resuspended in saline at the same concentration as live iMPs.

### iMP Characterization

iMPs were characterized by flow cytometry analysis (FACS), immunocytochemistry (ICC), and a phagocytosis assay. For FACS, cells were pelleted and washed 3x in tris buffered saline (TBS), before being resuspended in FC receptor blocking buffer (#BDB564219, Thermo Fisher Scientific) for 1 h. Antibodies against human CD11b (#101 228), CD34 (#343 512), CD14 (#325 618), CD16 (#302 044), and CD11c (#301 614; all from BioLegend) were then added to the blocking buffer, and samples were stored at 4 °C overnight. Cells were then pelleted and washed 3 times in TBS, fixed in 4% PFA for 10 min, and then resuspended in 1 mL of FACS buffer, comprised of 1X DPBS with 2% bovine serum albumin (BSA, #A7906, Sigma Aldrich) and 0.5 mM ethylenediamine tetraacetic acid (EDTA, #AM9260G, Thermo Fisher Scientific). Cells found to be positive for CD11b and negative for CD34 were gated and further analyzed for CD11c, CD14, and CD16. For ICC, iMPs were plated on poly‐lysine‐coated coverslips overnight before fixation in 4% PFA for 10 min. Cells were then rinsed in PBS and permeabilized using 0.1% Triton‐X 100 in PBS for 5 min. After blocking in 2% BSA for 1 h, cells were incubated overnight at 4 °C in primary antibodies directed against CD68 (#ab955, 1:200, abcam), CD11c (#NB110‐97871, 1:500, Novus Biologicals), IBA1 (NB100‐1028, 1:500, Novus Biologicals), and P2ry12 (#HPA014518, 1:2000, Sigma Aldrich). On the following day, coverslips were rinsed with PBS and incubated with Alexa‐Fluor conjugated antibodies diluted 1:500 in blocking solution for 2 h at room temperature followed by 4′,6‐diamidino‐2‐phenylindole (DAPI) counterstain. Phagocytosis was assayed by plating iMPs on poly‐lysine‐coated coverslips overnight and then adding pHrodo Red Zymosan Bioparticles (#P35364, Invitrogen) for 3 h at 37 °C, followed by fixation in 4% PFA for 10 min and ICC for CD68 as well as a DAPI counterstain.

### iMicroglia Differentiation and Treatments

iMicroglia (iMGL) were differentiated from iPSCs following the same protocol as for iMP differentiation (described above). After collecting free‐floating cells, these were plated at 50 000 cells/well in a 24‐well plate in RPMI‐1640 (#11 875 093, Gibco), supplemented with 2 mM GlutaMAX (#35 050 061, Gibco), 10 ng/mL GM‐CSF, and 100 ng/mL interleukin‐34 (IL34; #5265‐IL‐010, R&D Systems). After 1 week in culture, iMGL were treated with either 10 µg/mL human serum amyloid P component (APCS; #555 145, Millipore Sigma) and 50 ng/mL human serum amyloid A2 (SAA2; abx166518, abbexa), 20 µM camptothecin (CAMP; J62523.MD, ThermoFisher Scientific), or 40 ng/mL BSA. iMGL were treated either in RPMI as a vehicle or in iMP‐conditioned media, which was generated by plating 5 × 10^6^ iMPs in 10 mL RPMI for 48 h. After 24 h of treatment, cells were fixed for ICC with antibodies directed against IBA1 (#019‐19741, 1:250, Wako Chemicals), CD105 (#MAB1097, 1:250, R&D Systems), and CC3 (#9664, 1:1000, Cell Signaling Technology). Quantification of ICC staining was performed by capturing images of non‐overlapping fields across at least 3 coverslips for each condition, at 20x magnification for CC3/CD105 staining, and at 40x in oil for IBA1 branch analysis. CC3 expression was analyzed in DAPI+/CD105+ iMGL. The simple neurite tracer plugin in NIH ImageJ was used to trace IBA+ cell branches, and the average branch length was calculated. Separate plates of iMGL were assayed for phagocytosis using pHRodo zymosan beads (#P35364, ThermoFisher Scientific) and imaging on an Incucyte Live Cell Imaging platform 1 h after the addition of beads. The percent of cells expressing red fluorescent beads was quantified.

### Human Cell Tracking

To determine the fate of injected human cells, iMPs were differentiated from an iPSC line stably expressing a green fluorescent protein (GFP). GFP+ iMPs were administered at a dose of 12 million cells via tail vein injection, and tissues were collected 30 min later, for subsequent analysis via FACS or polymerase chain reaction (PCR).

Red blood cell lysis: Blood samples were collected from the left ventricle and stored on ice in Vacuette K2EDTA tubes (#454 052, Greiner Bio‐One). As a positive control, one sample from each animal was immediately spiked by adding 1 million GFP+ iMPs directly into the blood sample. Red blood cell lysis was achieved using the eBioscience 1X RBC Lysis Buffer (#00‐4333‐57, Thermo Fisher Scientific), with a ratio of 1 mL of blood to 10 mL of buffer. Following incubation at room temperature for 5 min, samples were transferred to a 15 mL conical tube with 10 mL of 1X DPBS to neutralize the reaction. Samples were spun at 800 rcf for 5 min and the supernatant was aspirated. Samples were washed with 1X DPBS and re‐spun using the same parameters. The resulting pellets were resuspended in 1 mL of FACS buffer. Samples were strained through a 35 µm nylon mesh strainer (#352 235, Corning) and vortexed immediately prior to loading onto a BD LSRFortessa Cell Analyzer.

DNA isolation and PCR: Tissues including liver, lungs, kidney, heart, spleen, brain, and tail, as well as whole blood and serum and plasma, were isolated and immediately snap frozen. Primers were designed to target primate specific Alu elements in order to detect human DNA in mouse tissue, a technique shown to detect small amounts of human cells transplanted into rodent tissues.^[^ ^145^ ^]^ Genomic DNA was isolated using the DNeasy Blood & Tissue Kit (# 69 504, Qiagen) according to manufacturer's instructions. Applied Biosystems TaqMan Fast Advanced Master Mix (#4 444 556, Thermo Fisher Scientific) was used to perform the amplification, following manufacturer's instructions. Thermal amplification was performed on a BioRad CFX384 Real‐Time System and conditions were as follows: 95 °C for 3 min of heat denaturation, followed by 40 cycles of annealing at 95 °C for 15 s and extension at 60 °C for 20s.

SC121 immunohistochemistry: Tissues were fixed in 4% PFA for 48 h, cryoprotected in 30% sucrose for 48 h, and then stored in 0.1 M PBS/0.03% NaN_3_. Tissues were separated into labeled cassettes and placed in TBS prior to the dehydration process. Tissues were then dehydrated using the ASP300 tissue processor (Leica CM3050S). After processing, tissues were embedded in paraffin wax and sectioned at  5 µm thickness using a Leica RM2235 manual rotary microtome and placed on slides using a Premiere XH‐1003 Tissue Floating Bath. Sectioned slides were dried at room temperature and warmed on a Premiere XH‐2003 Step‐Up Slide Warmer at 60 °C for 1 h. Slides were deparaffinized in 2 changes of xylene for 10 min each while on a shaker, then 2 changes in 100% ethanol for 5 min each, followed by 90%, 80%, and 70% ethanol washes for 3 min each. Antigen retrieval was performed using sodium citrate buffer (0.1 M sodium citrate, 0.1 M Citric Acid in TBS, pH 6.0) and microwave heated at 80% power for 20 min until sub‐boiling, then allowed to cool in buffer for 60 min. Slides were blocked in 5% NDS in 0.2% TX‐TBS for 1 h and then incubated in SC121 (#Y40410, 1:50, Takara Bio) overnight at 4 °C. Slides were then washed 3X before being incubated in secondary antibody Alexa Fluor 594 (#A21203, 1:500, Invitrogen) for 2 h, followed by counterstaining with DAPI (#D3571, 1:10 000, Invitrogen) and coverslipped using Fluoromount‐G (#00‐4958‐02, Invitrogen).

### Behavioral Analyses

Behavioral testing was performed as shown on the schematic depicting the treatment paradigm for each mouse model. All analyses were performed in a blinded manner, by 2–3 independent observers, and animals were habituated to the room for at least 30 min prior to beginning each day of testing.

Spontaneous alternation behavior (SAB): Mice were first evaluated in SAB as a test of spatial working memory. Animals were placed into an opaque Y‐maze and allowed to explore freely for 5 min. During this time, two independent observers recorded which arms the animals entered and behavior was scored as the number of complete alternations divided by the total number of arm entries minus 2, as the first two entries cannot be a complete alternation.

Contextual and cued fear conditioning: On day 1 of fear conditioning, mice were placed into and allowed to freely explore the conditioning chamber (San Diego Instruments), which had an electrified grid floor and was placed inside a sound‐attenuated chamber. A cotton ball with vanilla extract was placed below the grid floor as a scent cue. Mice were exposed to two pairings of a tone and light with a foot shock (30 s light and tone at 1000 Hz followed by 2 s foot shock at 0.7 mA), with a 180 s interval between. On day 2, contextual fear conditioning was assessed by placing the animals back into the conditioning chamber, which had the same appearance and odor as on the previous day, and freezing behavior was measured over 8 min. On day 3, cued fear conditioning was assessed by placing the animals into a novel context (an octagonal box was placed inside the chamber to change the floor and walls, the vanilla scent was removed, and the chamber was cleaned with isopropyl alcohol). Mice were then exposed to the same tone and light cues as on day 1. Freezing behavior was measured using the Freeze Monitor tracking system and software that measures infrared beam breaks (San Diego Instruments) and is presented as the percent time of the evaluation period in which mice exhibited freezing, as well as the number of freezing bouts.

Novel object location (NOL) and recognition (NOR): Short‐term memory was tested in the NOL and NOR assay, in which animals were first habituated to an open field chamber with opaque walls for 5 min. 30 min later, they were placed into the chamber with two identical novel objects they had never encountered before and allowed to freely explore for 3 min. After being placed back in their home cage for 30 min, mice were tested in the NOL task, where one of the objects was placed on the other side of the chamber while the other remained in its previous location, and mice were again allowed to explore freely for 3 min. Finally, for the NOR test, 30 min later, one of the objects was replaced with another new object while one remained the same. The amount of time mice spent with each object was recorded and their performance was calculated as the time spent with the novel object minus time spent with the familiar object and divided by the total time spent with both objects.

Elevated plus maze: Mice were placed in the center of a plus‐shaped maze that was raised 40 cm above the ground. Two of the arms have opaque walls that are 15 cm in height, while the other two arms are open. Mice were allowed to freely explore for 3 min and the number of entries to each arm as well as the time spent in open versus closed arms was recorded. The percent of time spent in the open arms and the percent of arm entries in which animals chose the open arm were calculated.

### Immunohistochemistry and Quantification

After fixation, hemibrains were cryoprotected in 30% sucrose for 48 h before being flash frozen on dry ice and sectioned in the horizontal plane at 30 µm using a Leica SM2010R freezing sliding microtome (Leica Biosystems). Sections were stored in 0.1 M PBS/0.03% NaN_3_ at 4 °C. Immunohistochemistry was performed as previously described,^[^ ^15^, ^118^ ^]^ with minor modifications as follows.

For NeuN and IBA1/LAMP1 immunohistochemistry, antigen retrieval was performed by boiling sections in 10 mM EDTA pH 6.0 for 10 min, then rinsing in water 3 times for 5 min each. Antigen retrieval was also performed for IBA1/COX1, DCX, and Aβ immunohistochemistry by pretreating sections with 95% formic acid for 5 min, while for Ki‐67, sections were boiled in sodium citrate buffer at 80 °C for 20 min, then cooled in buffer at room temperature for 20 min. After antigen retrieval, all sections were rinsed in TBS 3 times for 5 min each and treated with 3% H_2_O_2_/3% methanol for 10 min, followed by 3 washes in 0.2% Triton‐X/TBS for 10 min each. Blocking solutions for all antibodies consisted of 2% BSA in 0.2% Triton‐X/TBS, except for GFAP (5% normal donkey serum (NDS) in 0.3% Triton X/TBS), DCX (3% NDS in 0.2% Triton X/TBS), and GluR2/3 (2% BSA, 3% NDS in 0.2% Triton X/TBS). Sections were incubated in primary antibody overnight at 4 °C as follows: VGLUT1 (#135 304, 1:500, Synaptic Systems), GFAP (#AB5804, 1:2000, Millipore), NeuN (#MAB377, 1:500, Millipore), IBA1 ((#019‐19741, 1:250, Wako Chemicals), LAMP1 (#SC‐19992, 1:200, Santa Cruz Biotechnology), CD68 (#MCA1957, 1:200, Bio‐Rad Laboratories), COX1 (#160 110, 1:500, Cayman Chemicals), COX2 (#160 126, 1:200, Cayman Chemicals), DCX (#4604, 1:50, Cell Signaling), calretinin (#ab702, 1:500, abcam), GluR2/3 (#ab1506, 1:250, Millipore), Aβ (#71‐5800, 1:300, Invitrogen), and Ki‐67 (#15 580, 1:50, abcam). On the following day, sections were rinsed in 0.1% Triton‐X 3 times for 10 min each and then incubated with Alexa Fluor‐conjugated secondary antibodies diluted 1:500 in blocking solution for 2 h at room temperature. Sections were then washed and counterstained with DAPI before being mounted and coverslipped using Vectashield hardset antifade mounting medium (#H‐1400, Vector Laboratories).

For COX2 and Aβ, sections were rinsed, incubated in biotinylated secondary antibody diluted in blocking solution, followed by a standard avidin/biotin peroxidase approach using a Vectastain ABC kit (#PK‐4000, Vector Laboratories), and then immunoreactivity was visualized using 3,3′‐diaminobenzidine (#SK‐4100, Vector Laboratories). Sections were mounted, dried overnight, and dehydrated before being coverslipped in Epredia mounting medium (#22‐110‐610, Thermo Fisher Scientific).

For quantification of NeuN and GFAP, images of non‐overlapping fields of the hippocampal subregions CA1, CA3, and DG (3 fields per section) across 4 sections per mouse were digitally captured at 20x magnification using a Leica DM600B microscope. For DCX, calretinin, GluR2/3, and Ki‐67 non‐overlapping fields of dentate gyrus were acquired at 20x magnification across 4 sections per animal. Total numbers of NeuN+ nuclei, and GFAP+, DCX+, calretinin+, GluR2/3+, and Ki‐67+ cells were quantified using ImageJ. Astrocyte hypertrophy was assessed by outlining GFAP+ cell bodies. An average of 20 and 18 astrocytes were outlined in CA1 and CA3, respectively. For quantification of the area occupied by COX2 immunoreactivity (COX2 load), images of nonoverlapping fields were captured at 20x magnification. Images were digitally captured of the hippocampal subregion CA3 across 4 sections per animal, using a Leica AF3500 microscope. Pictures were converted to grayscale and thresholded to yield binary images separating positive and negative staining using NIH ImageJ 2.0. Similarly for Aβ, nonoverlapping images were captured of hippocampal subregions subiculum (3 fields), CA1 (3 fields), CA2/3 (3 fields), and DG (1 field) across 4 sections per animal. COX2 and Aβ load was defined as the percent of the total area occupied by positive immunolabeling.

Images of all remaining immunohistochemical staining (VGLUT1, IBA1/LAMP1, IBA1/CD68, and IBA1/COX1) were acquired using an A1R confocal microscope (Nikon). For VGLUT1, images were captured using a 40x magnification oil immersion lens at a step size of 0.33 µm. A maximum intensity projection image was generated using ImageJ and the region of interest was outlined by tracing 10 µm above and below the pyramidal cell layer of CA3. Images were converted to grayscale and thresholded to yield binary images separating negative and positive staining, and the percent area occupied by VGLUT1 staining was calculated. Three sections per mouse were analyzed.

For IBA1‐stained sections, images were acquired at 63x magnification under oil immersion and at a step size of 0.5 µm. Three sections per mouse and three fields per section (CA1, CA3, and DG) were imaged and analyzed. Microglial branches were traced using the Simple Neurite Tracer (SNT) plugin on ImageJ and the average branch length per brain region for each animal was calculated. Sholl analysis was performed by randomly selecting three microglia, centering the traced branches into a shared root and running the Sholl analysis function using the SNT plugin. The resulting Sholl profiles provided intersection values which were then used to calculate the area under the curve using the trapezoid rule.^[^ ^146^ ^]^ Output values were averaged across the three traced microglia. Microglia tracing and Sholl analysis were also performed in DG of BALB/c mice, both from the 3‐week‐ and 10‐week‐treated animals. As there were no significant differences between 3‐ and 10‐week‐treated mice within each experimental group, mice from both timepoints were combined in all histological analyses. For IBA1/LAMP1, IBA1/CD68, and IBA1/COX1, images were acquired as described above for IBA1, and the percentage of IBA1+ cells that expressed either LAMP1, CD68, or COX1 was calculated.

Additionally, the area of LAMP1 immunoreactivity within IBA1+ cells was analyzed in hippocampal DG. Briefly, images were acquired at a step size of 0.3 µm using a Leica Stellaris 8‐STED Super‐resolution confocal microscope and analyzed using ImageJ. Z‐stack images were first preprocessed using the Despeckle filter and the Median filter set at 0.5. The ROI tool was used to outline cells on a maximum intensity composite image, while scanning through the z‐stack to ensure that all included cells were entirely within the z‐stack. Maximum intensity projection images were created for each cell to ensure that only particles within the cell were labeled and counted, and images were thresholded. The Analyze Particles function was used to measure the area of LAMP1+ particles inside the ROIs. A minimum of *n* = 5 cells/animal was analyzed.

### Protein Extraction and Multiplex ELISA

Cortical protein extraction & cytokine analysis: Cortical samples from NSG mice were weighed and homogenized in 1 mL of Tris lysis buffer (150 mM NaCl, 1 mM EDTA, 1 mM EGTA, and 1% Triton‐X in 20 mM Tris, pH 7.5) per 100 mg tissue. Each 10 mL Tris lysis buffer was supplemented with 2 tablets of Roche cOmplete EDTA‐free protease inhibitor cocktail (#11 836 170 001, Millipore Sigma), and 1 tablet of Roche PhosSTOP (#4906845001, Millipore Sigma). Tissue was sonicated on ice using a Model 50 Sonic Dismembrator with a 1/8‐inch diameter probe (#FB50110, Fisher Scientific) with 3 × 30 sec bursts, at 40% amplitude. Sonicated samples were then rotated at 4 °C for 30 min before being centrifuged at 20 000 x g for 10 min at 4 °C. The supernatant was aliquoted and stored at −80 °C. Protein concentration was measured in triplicate using the Pierce dilution‐free rapid gold BCA protein assay (#A55860, Thermo Fisher Scientific). Samples were assayed in duplicate for the following targets: IL33, CXCL10, CCL3, CXCL1, CXCL2, IFNγ, IL10, TNFα, IL1β, and IL6, using custom arrays of the V‐PLEX Mouse Cytokine 19‐Plex kit (#K15255D, Meso Scale Discovery), according to manufacturer's instructions.

Soluble Aβ: Hippocampal and cortical tissue samples were combined for each young and aging 5xFAD animal. Tissue was homogenized in cold TBS lysis buffer containing 1 mL of each inhibitor per 97 mL TBS lysis buffer: Protease Inhibitor Cocktail I (#539 131), Phosphatase Inhibitor Cocktail 2 (#P5726), and Phosphatase Inhibitor Cocktail 3 (#P0044); all from Millipore Sigma. Tissue were manually lysed with 30 strokes in a 2 mL glass dounce homogenizer and were then passed through Pasteur pipets 10 times while kept on ice. Samples were placed in 1.5 mL ultracentrifuge tubes (#357448, Beckman Coulter) and centrifuged at 100 000 x g for 1 h in an Optima MAX‐TL ultracentrifuge (Beckman Coulter). The supernatant (TBS‐soluble fraction) was removed, aliquoted, and stored at −80 °C. The pellet was then broken up in TBS with 1% Triton‐X, passed through a Pasteur pipette 20 times, and placed in new ultracentrifuge tubes. Samples were placed on a shaker for 30 min at 4 °C and then centrifuged at 100 000 x g for 1 h. The resulting supernatant (TX‐soluble fraction) was aliquoted and stored at −80 °C. Protein concentration was assayed by BCA, and samples were run in duplicate using the Aβ (4G8) V‐Plex (#K15199E, Meso Scale Discovery) plate, according to manufacturer's instructions.

### Proteomic Analyses

Discovery proteomics on plasma: Plasma samples were processed using Protifi S‐TRAP sample preparation and trypsin digestion workflow as follows: 10 µL of plasma was diluted into 40 µL 6 M Urea / 5% SDS lysis buffer, protein concentration was estimated using a Pierce BCA assay (#23225, Thermo Fisher Scientific), and 100 µg was aliquoted for digestion. Proteins were reduced with 100 mM dithiothreitol (DTT), alkylated with 200 mM iodoacetamide (IAA), and digested with 5 µg trypsin. Tryptic peptides were eluted from S‐trap micro columns (#C02‐micro‐10, Profiti), dried, and resuspended in 0.1% formic acid water at 1 µg/µL concentration prior to liquid chromatography‐mass spectrometry (LC‐MS) analysis.

Mass spectrometry data were acquired on a Fusion Lumos Orbitrap instrument. Desalted peptides were separated on an Ultimate 3000 liquid Chromatography system with a 60‐min gradient. Peptides were separated on a preformed gradient (ranging from 0 to 45% organic phase) on a µPAC HPLC column (#COL‐CAP050G1B, Thermo Fisher Scientific) at a flow rate of 9.5 µL/min. Spray voltage was set at 3.9 kV and an ion transfer temperature of 290 °C. MS1 resolution was set to 120 000 and automatic gain control (AGC) was set to 600 000 (150% normalized AGC target) with maximum injection time of 50 ms. RF lens % was set to 30. Mass range of 400–1000 and AGC target value for fragment spectra of 400% were used. Peptide ions were fragmented using higher energy C‐trap dissociation (HCD) at a normalized collision energy of 30%. Fragmented ions were detected across 40 data‐independent acquisition (DIA) windows of 15 m/z. MS2 resolution was set to 15 000 with a max injection time of 30 ms in centroid mode.

A sample specific library was generated using DIA‐Umpire‐based signal extraction, followed by matching of DIA‐Umpire pseudospectra (from Q1 files only) using the Trans Proteomic Pipeline (TPP, v5.2.0), spectral matching algorithms Comet, and X!Tandem. Peptide‐level target‐decoy probability scoring was performed by peptide prophet in the TPP,^[^ ^147^ ^]^ run individually on each search algorithm run, and then results of multiple searches were combined using the TPP InterProphetParser. Peptides with probability >0.95 were compiled into a preliminary library using TPP SpectraST and retention times were aligned to iRT using Biognosys iRT standard peptides (#Ki‐3002‐1, Biognosys). iRT aligned libraries were consolidated and converted to TraML format and randomized decoy sequences were appended. The sample specific library was then searched against each individual DIA file using openSWATH peak picking and scoring algorithm.^[^ ^148^ ^]^ Decoy‐target probability modeling was done using pyProphet algorithm^[^ ^149^ ^]^ and results from individual files were aligned across the experiment using the TRIC workflow.^[^ ^150^ ^]^ Following normalization to total MS2 signal, mapDIA^[^ ^151^ ^]^ was used to perform protein abundance inference and statistical comparisons. Proteins differentially expressed between groups were converted to genes and analyzed via Gene Ontology (GO) Enrichment Analysis using PANTHER v17.^[^ ^152^ ^]^ Four annotation data sets were used: GO biological process, GO molecular function, GO cellular component, and Reactome pathways. The web‐based tool ImageGP^[^ ^153^ ^]^ was used for GO term visualization.

Discovery proteomics on conditioned media: iMPs were plated in 1/3 volume of media (i.e., 1 × 10^6^ cells in 333 µL RPMI) for 48 h. Media was collected and centrifuged to remove any cells and debris. Samples were digested by SP3 protocol. Briefly, 20 µg of protein was brought up to 60 µL volume using 6 M urea, 1 M ammonium bicarbonate, and 5% SDS lysis buffer. The digest was reduced with the addition of 16.8 µL of 200 mM dithiothreitol and incubated 30 min at 37 °C with shaking at 300 rpm, then alkylated with 21.2 µL of 400 mM IAA at room temperature for 30 min in the dark. The volume was brought to 160 µL with Tris‐HCl pH 8, then 5 µL of bead suspension (10:1 mass ratio of beads to protein, 1:1 mixture of hydrophilic/hydrophobic beads (Cytiva)) was aliquoted into the samples and vortexed. Samples were brought to 70% CAN v/v and incubated for 18 min, and then the solvent was removed on‐magnet, and samples were rinsed with 2 × 80% EtOH then 2 × ACN with 200 µL volumes each. After the solvent was completely removed, the samples were resuspended in 50 mM Tris‐HCl pH 8, and 10 mM CaCl2 with trypsin at a 1:20 ratio. Samples were bath sonicated for 5 min, then incubated 18 h at 37 °C and 1200 rpm overnight. After digestion, the samples were then removed from beads and brought to 0.1% FA and 2% DMSO for injection into the instrument.

DIA analysis was performed on an Orbitrap Astral (Thermo Scientific) mass spectrometer interfaced with an EASY‐Spray nano‐electrospray ionization source (Thermo Scientific, ES081) coupled to Vanquish Neo ultra‐high‐pressure chromatography system with 0.1% formic acid in water as mobile phase A and 0.1% formic acid in acetonitrile as mobile phase B. Peptides were separated at an initial flow rate of 1.2 µL/minute and a linear gradient of 4–9% B for 0–2 min, 9–25% B for 2–18 min, 25–35% B for 18–27 min. The flow rate was then increased to 3 µL/min and the column was then flushed with 35–99% B for 0.4 minutes, then held at 99% B for 0.5 min, decreased to 5%, and the zebra wash flushing was repeated two more times. The column used was PepSep C18 15 cm x 150 µm, 1.5 µm (#1 893 474, Bruker). Source parameters were set to a voltage of 2300 V and a capillary temperature of 280 °C. MS1 scan range was set to 360–1200 m/z, and MS1 resolution was set to 240 000 with an AGC target set to “Custom” and a normalized AGC target set to 500%. RF Lens was set to 40% with maximum injection time set to 10 ms. Precursor mass range was set to 380–980 m/z with 199 non‐overlapping data independent acquisition precursor windows of size 3 m/z. MS2 scan range was set to 150–2000 m/z and normalized HCD collision energy set to 30%. Maximum injection time was set to 2.7 ms with AGC target set to “Custom” and normalized AGC target set to 500%. All data was acquired in profile mode using positive polarity.

MS raw data files were searched against UniProt human reviewed protein sequence entries (accessed April 2023) using DIA‐NN (v 1.8.1)^[^ ^154^ ^]^ in library free mode with default parameters. Based on recent comparisons with library‐based approaches, DIA‐NN in library‐free mode has been found to produce results that are comparable to or better than those of experimental library‐based searches while being freely available; hence, this mode was chosen for the analysis of all data.^[^ ^155^ ^]^ The output protein group matrix from DIA‐NN was used to perform downstream analysis using MetaboAnalyst 6.0.^[^ ^156^ ^]^

Targeted proteomics: The samples were analyzed by targeted liquid chromatography mass spectrometry (LC‐MS) using a rapid chromatographic separation and parallel reaction monitoring (PRM) on a Lumos Orbitrap Fusion. The LC separation used the dual trap single column configuration (DTSC),^[^ ^157^ ^]^ with 0.68 µL Exp2 Stem trapping column cartridges (#15‐04009, Optimize Technologies) filled with PLRP 10 µm reversed phase beads and a 150 × 0.3 mm Kinetex 2.6 µm Polar C18 LC column (#00F‐4759‐YO, Phenomenex). With DTSC parallelization, one sample is analyzed while the subsequent sample is loaded on the second column, an identical gradient is delivered through the second trapping column in the subsequent run, and the roles of the trapping columns are alternated for the entirety of the sample set. The analytical reversed phase gradient used 0.1% formic acid in water as mobile phase A and 0.1% formic acid in acetonitrile as mobile phase B was delivered at 15 µL/min as follows: start at 9% B, linear ramp to 30% B over 10.2 minutes, ramp to 42% B over 0.65 minutes, organic flush with 95% B for 0.5 min at 20 µL/min, return to 10% B and 15 µL/min in 0.05 min and 0.1 min hold (11.5 min method total with ≈0.5 min of instrument warm‐up; 12 min/sample). The columns were kept at 52 °C in the Ultimate 3000 column oven.

The eluting peptides were electro‐sprayed through a 5‐nozzle (20 µm inner diameter) M3 emitter (#E5N20MU01, Newomics) at a positive voltage of 4200 V and sheath gas set to 20 (arbitrary units). An MS1 scan was acquired during each acquisition cycle at a 60 000 (m/z = 200) resolution, 400 000 AGC, and 100 ms maximum injection time, followed by targeted fragmentation of 0.8 m/z wide isolation windows corresponding to the peptides of interest during their determined elution times with a 0.2 min buffer added on each side of the chromatographic peak. The PRM scans were acquired at 30 000 resolution (m/z = 200), with AGC set to 500 000, max injection time of 200 ms, and HCD collision energy set to 32%. The PRM runs were imported into Skyline^[^ ^158^ ^]^ and the cumulative fragment ion signal was used for relative quantitation of proteins between samples.

### Single Nucleus RNA Sequencing of Hippocampus

Nuclei isolation: Hippocampal nuclei were isolated using a modified version of a published protocol.^[^ ^159^ ^]^ All steps were performed on ice. Wide‐bore Rainin LTS pipette tips were used exclusively throughout, except where indicated. Frozen hippocampal tissues were thawed on ice, resuspended in cold homogenization buffer (5 mM MgCl2, 3 mM Mg(Ac)2, 10 mM Tris pH7.8, 0.017 mM phenylmethylsulfonyl (PMSF), 0.17 mM b‐mercaptoethanol, 320 mM sucrose, 0.1 mM EDTA, 0.1% NP40, RNAse and protease inhibitors), and transferred to a 2 ml glass dounce homogenizer. Tissues were manually lysed with 50 strokes each of the “A” and “B” pestles. Homogenate was filtered using 70 µm Flowmi pipette tip filters (#BAH136800070, Sigma Aldrich) and gently mixed 1:1 with a 50% Iodixanol solution (5 mM MgCl2, 3 mM Mg(Ac)2, 10 mM Tris pH7.8, 0.017 mM PMSF, 0.17 mM β‐mercaptoethanol, RNAse and protease inhibitors, and 50% Iodixanol). A centrifugation gradient was set up using 600 µL 40% Iodixanol solution (5 mM MgCl2, 3 mM Mg(Ac)2, 10 mM Tris pH7.8, 0.017 mM PMSF, 0.17 mM b‐mercaptoethanol, RNAse and protease inhibitors, 160 mM sucrose, 0.2 ug/ul OrangeG, and 40% Iodixanol), 600 µL 29% Iodixanol solution (5 mM MgCl2, 3 mM Mg(Ac)2, 10 mM Tris pH7.8, 0.017 mM PMSF, 0.17 mM β‐mercaptoethanol, RNAse and protease inhibitors, 160 mM Sucrose, and 29% Iodixanol) and 800 µL of the 50% Iodixanol + Sample mixture (25% Iodixanol final concentration). This was centrifuged at 3000 x g for 1 h with the breaks disengaged. Any debris on the top surface was removed, and 200 µL of the thin cloudy layer containing the nuclei was extracted with a regular bore pipette tip, mixed with 1.8 mL PBS + 1% BSA solution with a wide bore pipette tip, and centrifuged at 500 x g for 10 min with breaks engaged.  1.8 mL supernatant was removed, and the remaining 200 µL plus nuclei pellet was resuspended in 1.8 mL PBS + 1% BSA solution.  The nuclei suspension was counted via hemocytometer and checked for nucleus quality.  A portion of this suspension was then diluted to achieve the correct nuclei concentration for the 10x Chromium NextGEM 3′ protocol with a target of ≈4000 nuclei per sample.

Single nuclei library preparation and sequencing: The standard 10x protocol was used per the “Chromium NextGEM Single Cell 3′ Reagent Kits v3.1 User Guide, Rev D” (single index).  Briefly, nuclei were resuspended in the master mix and loaded together with partitioning oil and gel beads into the chip to generate the gel bead‐in‐emulsion (GEM).  The poly‐A RNA from the nucleus lysate contained in every single GEM was retrotranscripted to cDNA, which contains an Illumina R1 primer sequence, Unique Molecular Identifier (UMI), and the 10x Barcode.  The pooled, barcoded cDNA was then cleaned up with DynaBeads MyOne Silane (#37002D, Thermo Fisher Scientific), amplified by PCR, and the appropriately sized fragments were selected with SPRIselect reagent (#B23318, Beckman Coulter) for subsequent library construction. The amplified cDNA and sequencing libraries were quality checked on an Agilent 2100 BioAnalyzer (Agilent Technologies) using an Agilent High Sensitivity DNA Kit (#NC1738319, Thermo Fisher Scientific). Barcoded sequencing libraries were quantified by qPCR on a QuantStudio12k Flex (Thermo Fisher Scientific) system using the Collibri Library Quantification Kit (#A38524500, Thermo Fisher Scientific). The uniquely indexed libraries were pooled at equal ratio and sequenced on a NovaSeq 6000 (Illumina) as per the Single Cell 3′ v3.1 Reagent Kits User Guide, with a sequencing depth of ≈50 000 reads/cell. Raw sequencing data were demultiplexed and converted to FASTQ format using bcl2fastq v2.20.

Data analysis: Demultiplexed fastq files were run via CellRanger v7.1.0 using the “cellranger count” command with default options using the precompiled CellRanger mouse reference sequence “refdata‐gex‐mm10‐2020‐A” provided on the 10x Genomics website. CellRanger output “filtered_feature_bc_matrix files” (barcodes.tsv.gz, features.tsv.gz & matrix.mtx.gz) were loaded into the Seurat R package (v4.1.1, R v4.3.1, RStudio v2023.06.2, Build 561), keeping all genes that were expressed in more than one cell. All samples (5 young vehicle‐treated, 6 aging vehicle‐treated, and 5 aging iMP‐treated) were merged into a single Seurat object (11 8443 nuclei) and filtered by removing all cells with counts, genes, mitochondrial gene percentage, and ribosomal gene percentage outside of three standard deviations from the mean (11 4199 nuclei). The filtered Seurat object was normalized via the SCTransform() function using mitochondrial gene percentage as a regression variable. Principal component analysis (PCA) was run using 50 principal components (PCs). The number of “meaningful PCs” used in subsequent analyses was determined by comparing the actual contributed variance of each PC versus the hypothetical situation where each PC would contribute equally to the variance. 13 PCs contributed more variance than the calculated hypothetical equal‐variance level and were considered “meaningful”. Next, the nuclei were clustered using the Seurat functions RunUMAP(), FindNeighbors(), and FindClusters(). One sample from the aging vehicle‐treated group was observed to be clustering very differently from all the other samples, and this sample was removed as an outlier. The remaining 15 samples (5 per group) were reloaded from the Cellranger output, merged, and QC filtered as described above (105951 nuclei). The same processing steps for normalization and clustering were used (13 PCs). For FindClusters(), a resolution factor of 0.6 was used, resulting in the identification of 25 clusters. Clusters were identified by known cell type markers as listed below. Four clusters were removed due to the presence of marker genes from multiple cell types, indicating low‐quality nuclei, debris, or multiplets.

The remaining 105414 high‐quality nuclei were reprocessed as described above. The same number of PCs and resolution were determined optimal (13 PCs and a resolution of 0.6) and resulted in 23 clusters. For post‐clustering analysis, the RNA slot was processed with the Seurat functions NormalizeData(), FindVariableFeatures(), and ScaleData() using all genes for the “features” option of ScaleData(). Cluster markers were determined using the FindAllMarkers() function, and known marker genes were used to determine cell types. Clusters found to be of the same basic cell type were combined. 15 cell types were identified using the following marker genes: Gad1 & Gad2 for inhibitory neurons (INHN); Dlx1 & Col19a1 for basket cells (BSKT); Cnr1 & Calb2 for mossy cells (MOSS); Slc17a7 & Neurod6 for excitatory neurons (EXCN); Grm1 & Satb2 for pyramidal neurons (PYR); Calb1 & Prox1 for granule cells (GRN); Tmem119 & Itgam for microglia (MG); Aqp4, Gfap, & S100b for astrocytes (ASTR) and the addition of C3 for A1‐astrocytes (ASTR‐A1); Mbp & Mog for oligodendrocytes (OLIG); Olig1 & Sox10 for oligodendrocyte precursor cells (OPC); Pdgfra & Slc6a13 for vascular leptomeningeal cells (VLMC); Hemk1, Acad8, Folr1, & Kl for type‐a choroid plexus cells (CPCa) and loss of Hemk1 & Acad8 for type‐b choroid plexus cells (CPCb); and Kcnj8 & Rgs5 for pericytes (PERI).

Subsetting was performed for the mossy cells. After subsetting, the nuclei were reprocessed as above: SCTransform(), PCA, significant PC determination (14 PCs), UMAP, FindNeighbors(), and FindClusters() (0.2 resolution), followed by normalizing, centering, and scaling the RNA slot, resulting in 13 subclusters. Subcluster markers were determined via FindAllMarkers(). Differentially expressed genes in each subcluster were determined using the FindMarkers() function between the pairwise groups (Young Veh versus Aging Veh, Aging iMPs versus Aging Veh, Young Veh versus Aging iMPs). Gene Ontology (GO) Enrichment Analysis was performed using PANTHER v17. Other overrepresentation analyses were performed using Enrichr. ImageGP was used for the visualization of these analyses. Volcano plots were generated using the EnhancedVolcano R package (v1.18.0), and heatmaps were generated using the DoHeatmap() function.

### Determination of Transcriptomic Aging Clock Genes

Method and code were adapted from Buckley et al.^[^ ^43^ ^]^ The raw counts from the RNA slot of the filtered, clustered, and cell‐typed Seurat object were exported to a dataframe grouped by sample, type (young vehicle‐treated, aged vehicle‐treated, or aged iMP‐treated), and cell type. For each sample and cell type combination, pseudocells were generated by randomly selecting 15 cells at a time and aggregating gene expression across all 15 cells. Selection was performed without replacement unless no more cells remained in that group. 100 pseudocells were generated for each sample and cell type combination. Chronological age in months since birth was added to the metadata, and “leave one out” cross‐validation was performed using the young vehicle‐treated and aged vehicle‐treated pseudocells. For cross‐validation, one young vehicle‐treated and one aged vehicle‐treated animal were randomly paired five times without replacement (five pairs). The remaining eight animals not in each pair were designated as the training samples (five groups of eight), and each pair as the test samples. Each training group was used to generate a model of aging for each cell type using the glmnet R package (v4.1‐8) as described in Buckley et al.^[^ ^43^ ^]^ These models were used to predict the transcriptional ages of the held‐out test pair, and these predictions were compared to their chronological age using Pearson correlation. All but two cell types (CPCb and PERI) had an R value >0.8, indicating a strong positive correlation between the predicted transcriptional age and the chronological age of the samples for these cell types. For the prediction of the aging iMP‐treated samples, all ten vehicle‐treated animals (five young & five aging) were used to generate the main aging model for each cell type. These models were used to predict the transcriptional ages of each sample and cell type combination, and the predicted ages were plotted via the geom_tile() function of the R package ggplot2 (v3.4.4). The “variable importance” of all genes in each cell type aging model was determined using the vi_model() function of the vip R package (v0.4.1). Genes with non‐zero variable importance were extracted and analyzed as described for other sets of genes.

### Statistical Analysis

Significance testing for snRNA‐seq was calculated using the default Seurat method Wilcoxon Rank Sum test, and only adjusted *p* values < 0.05 were considered significant. Adjusted *p* values from Seurat were used in all snRNA‐seq figures, even when generated in Prism. All other data were analyzed using Prism software (version 10, GraphPad Software) and one‐way analysis of variance (ANOVA), except for data on 5xFAD mice, where young and aging animals were run at different times and using different batches of iMPs; thus, *t*‐tests were used to analyze effects of iMPs at each age. In the case of significant main effects, comparisons between groups were made using Tukey's multiple comparisons test correction. All data are represented as the mean ± standard error of the mean (SEM), and significance was set at a threshold of adjusted *p* values < 0.05. Data distribution was assumed to be normal, but this was not formally tested. Studies were performed blindly with multiple investigators confirming behavioral analyses and immunohistochemical quantification. The *n* for each experimental group is given in the figure legends. For all analyses, measurements were taken from distinct samples.

## Conflict of Interest

V.A.M., H.S.G., and C.N.S. have a patent around using induced mononuclear phagocytes in neurodegenerative diseases (US Patent Application no. 63/234,984). None of the authors has received income from this patent.

## Author Contributions

R.M.L. and J.I. contributed equally to this work.V.A.M., H.S.G., and C.N.S. contributed to the conceptualization of the study. Methodology was developed by V.A.M., R.M.L., L.J.D.H., J.I., S.K., S.B., G.L., and C.N.S. Validation of the results was carried out by V.A.M., R.M.L., L.J.D.H., E.V., M.A., J.I., and G.L. Formal analysis was performed by V.A.M., L.J.D.H., S.B., and S.J.P. The investigation was conducted by V.A.M., R.M.L., S.B., L.J.D.H., J.I., E.V., M.A., G.L., and S.K. C.N.S. provided the necessary resources for the study. Data curation was managed by V.A.M. and S.B. The original draft of the manuscript was written by V.A.M. Review and editing of the manuscript were performed by V.A.M., S.J.P., H.S.G., and C.N.S. Visualization of the data was created by V.A.M. and S.B. Supervision of the project was provided by V.A.M. and C.N.S. Project administration was handled by V.A.M. and L.J.D.H. Funding for the project was acquired by C.N.S.

## Supporting information



Supporting Information

## Data Availability

All snRNA‐seq data are deposited and available under GSE220548 (secure token to allow access to reviewers: alirqqwkvfcvjuh). The mass spectrometry proteomics data have been deposited to the ProteomeXchange Consortium via the PRIDE partner repository with the dataset identifier PXD052723 (secure token to allow access to reviewers: SqTnssPTiDeb). All processed proteomics data and R scripts are deposited on GitHub.com and publicly available under the following link: https://github.com/shaughnmb/2024_moser_et_al/.
