# Supplementary material for: Human iPSC‐Derived Mononuclear Phagocytes Improve Cognition and Neural Health across Multiple Mouse Models of Aging and Alzheimer's Disease
Source: Adv Sci (Weinh). 2025 Aug 24;12(41):e17848. doi: 10.1002/advs.202417848 (PMC12591165; doi:10.1002/advs.202417848)
Supplement: Supplementary file 1 — Supporting Information [file ADVS-12-e17848-s001.pdf]

Supporting Information

Human iPSC-Derived Mononuclear Phagocytes Improve Cognition and Neural Health Across Multiple Mouse Models of Aging and Alzheimer's disease

V. Alexandra Moser, Luz Jovita Dimas-Harms, Rachel M. Lipman[1], Jake Inzalaco[1], Shaughn Bell, Michelle Alcantara, Erikha Valenzuela, George Lawless, Simion Kreimer, Sarah J. Parker, Helen S. Goodridge, Clive N. Svendsen**

Supplemental Figure 1

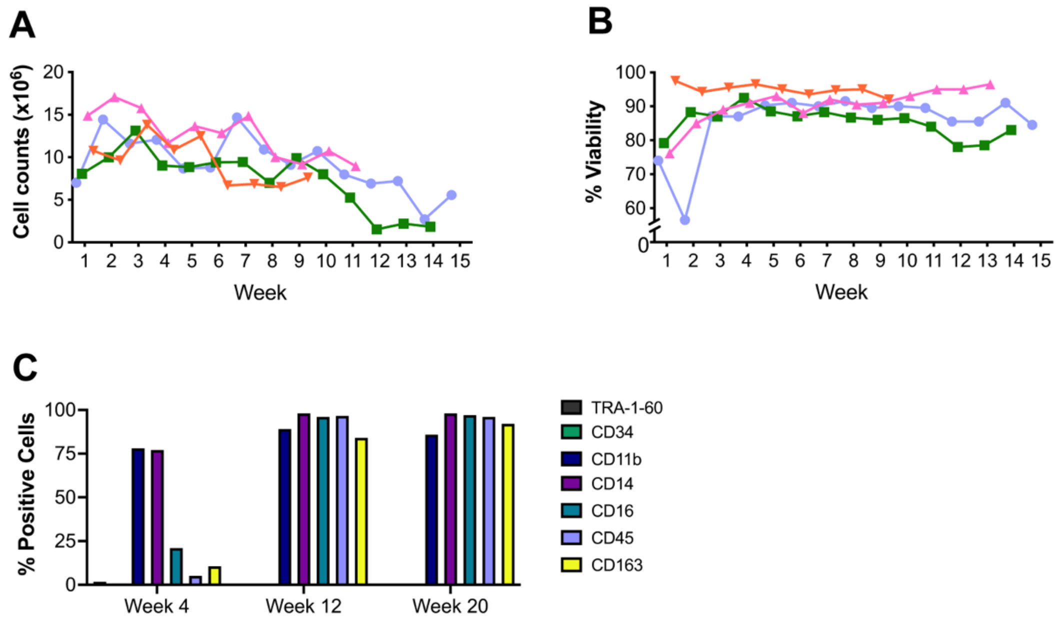

Figure S1. A) iMP cell counts over weeks in culture. Lines represent individual differentiations. B) iMP viability over time in culture. C) iMPs consistently expressed high levels of monocyte/macrophage cell markers over 20 weeks in culture.

Supplemental Figure 2

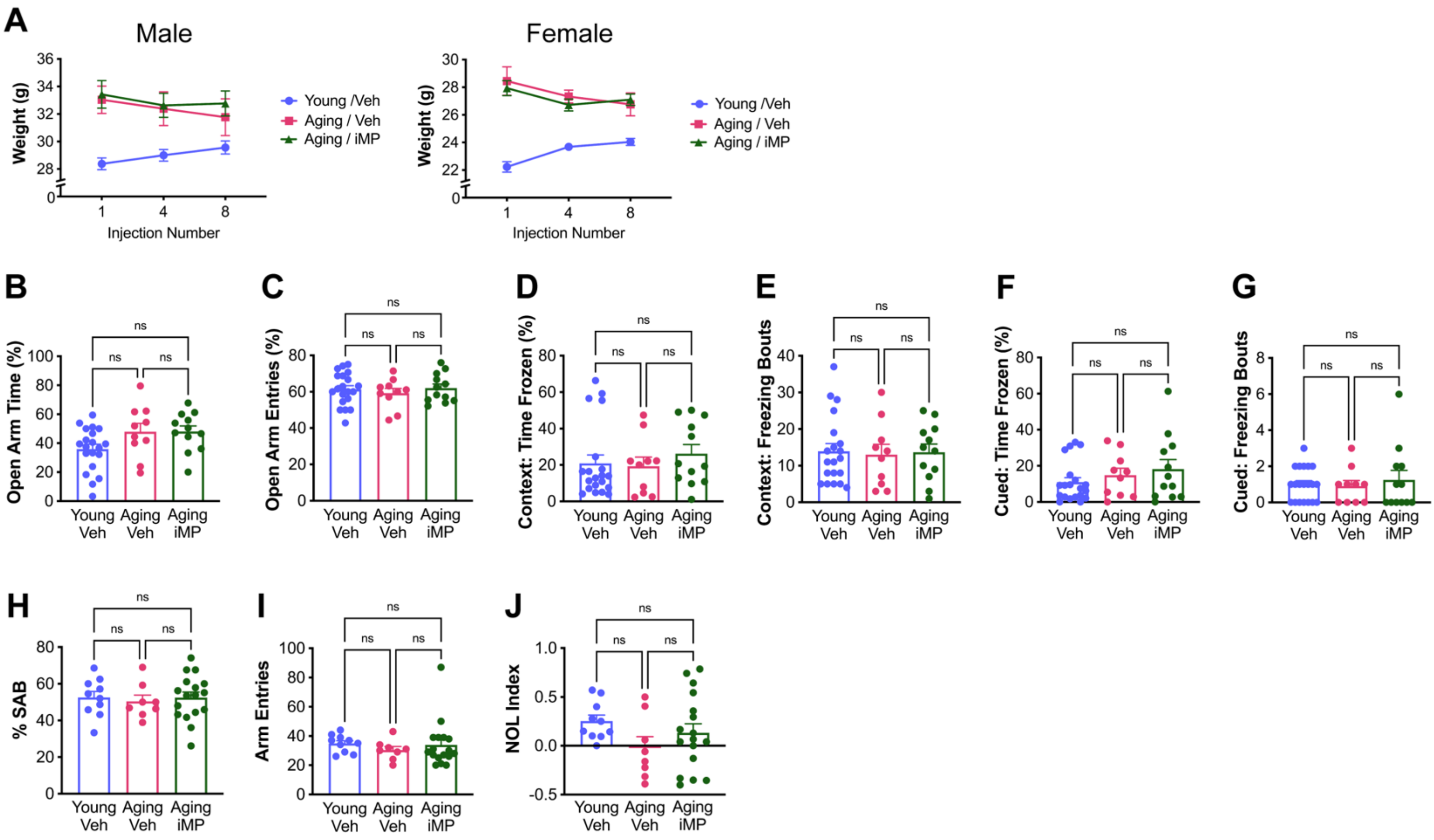

Figure S2. A) iMP treatment did not significantly affect body weights of male (left) or female (right) NSG mice. Behavioral analyses are shown in B-G for male NSG mice and in H-J for female NSG mice. B-C) Elevated plus maze. No group differences were found on either (B) percent time spent in the open arm or (C) percent of open arm entries. D-G) Contextual and cued fear conditioning. There were no significant effects of either age or treatment on measures of (D&F) percentage of time spent freezing or (E&G) number of freezing bouts. H-I) There were no significant effects of aging on spontaneous alternation behavior (SAB) or total arm entries in the Y maze in female NSG mice. J) Effects of aging and iMP treatment on novel object location performance did not reach statistical significance in female mice. Data are presented as mean (±SEM) values; *n*=10-20/group for male NSG mice, and *n*=8-17/group for female NSG mice. ns = not significant; one-way ANOVA with Tukey's multiple comparisons test correction.

Supplemental Figure 3

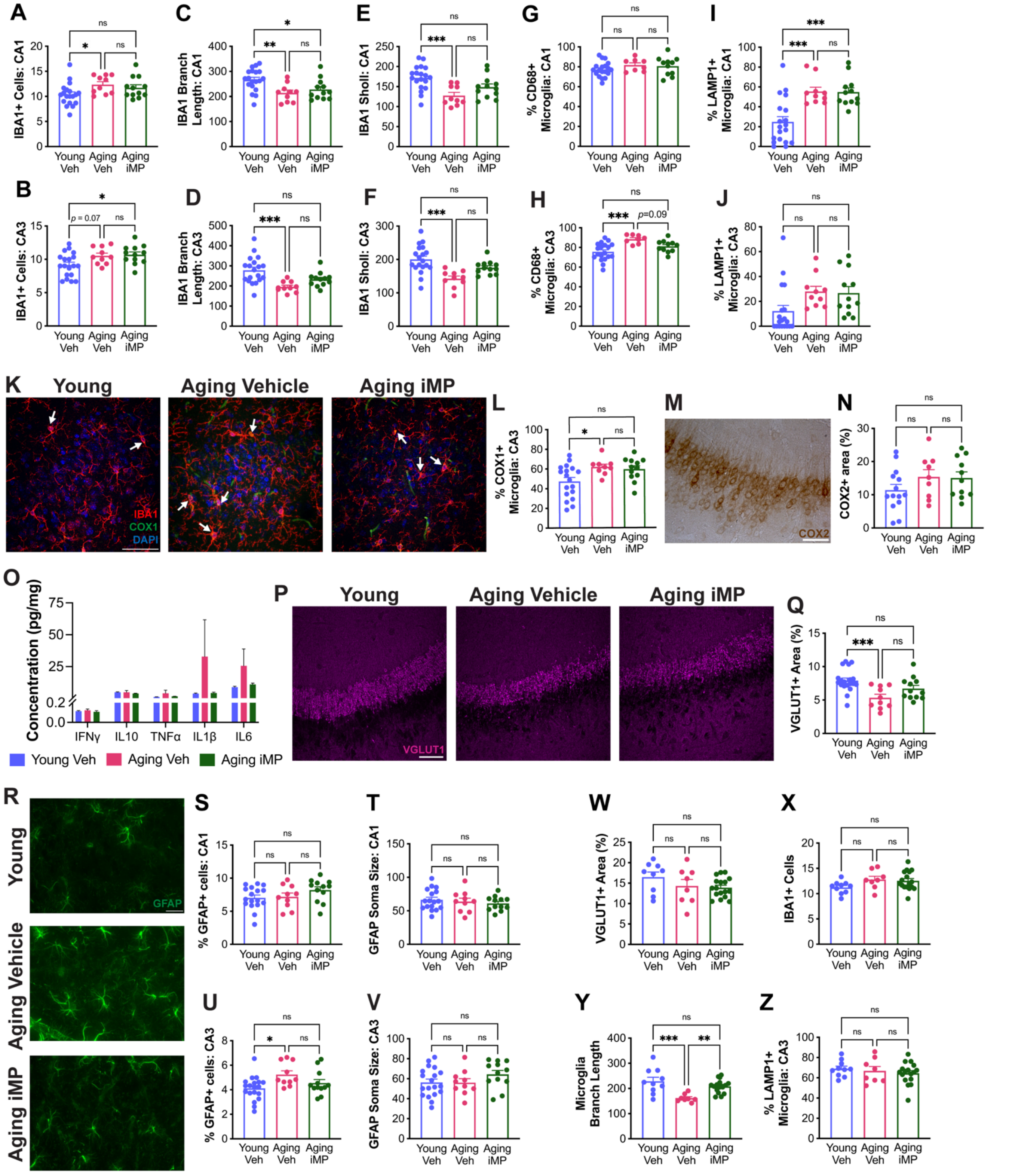

Figure S3. A-B) Quantification of microglial number in hippocampal CA1 and CA3. IBA1+ microglia were significantly increased in aging vehicle- but not iMP-treated mice in CA1 (A), and were increased in both aging groups in CA3, reaching significance specifically in iMP-treated mice (B). C-D) Microglial branch length was significantly reduced in both aging groups, irrespective of treatment in CA1 (C), but was only significantly reduced in aging vehicle-treated mice in CA3 (D). E-F) Sholl analysis revealed that branch complexity was significantly decreased in aging vehicle-treated mice but was partially rescued by iMP treatment in both CA1 (E) and CA3

(F). G-H) Microglial CD68 expression in CA1 (G) did not differ by age or treatment, while in CA3 (H) vehicle- but not iMP-treated aging mice had significantly more IBA1+ cells double-labelled for CD68 than young mice, and there was a trend towards reduced levels of CD68 in iMP- versus vehicle-treated aging mice. I-J) CA1 LAMP1 expression was significantly increased in both aging groups (I) but did not differ across groups in CA3 (J). K) Images showing staining for the microglial marker IBA1 (red) and COX1 (green); scale bar = 50μm. Arrows indicate double-labeled cells. L) The percent of microglia expressing COX-1 was significantly increased in vehicle-treated but not in iMP-treated aging mice. M) Image showing immunohistochemical staining for COX2 in hippocampal CA3. Scale bar = 50μm. N) COX2 levels did not vary across groups. O) Cytokine levels in cortical protein lysates did not significantly differ across groups. P) Images of immunohistochemical staining for the glutamatergic synapse marker VGLUT1 (purple) in hippocampal CA3. Scale bar = 50μm. Q) The percent area of VGLUT1 staining is significantly reduced in aging vehicle-treated but not in aging iMP-treated mice. R) Images of staining for the astrocyte marker GFAP in hippocampal CA3. Scale bar = 25μm. S-V) Quantification shows that while the number and size of astrocytes does not differ across groups in CA1 (S-T), in CA3, vehicle-treated but not iMP-treated aging mice have increased numbers of GFAP+ cells as compared to young mice (U), but GFAP soma size does not vary across groups (V). An average of 13 GFAP+ cells per mouse were analyzed for soma size; each dot shows average data from one mouse. W-Z) neural and microglial health outcomes in female NSG mice. W) The percent of VGLUT+ staining did not differ across groups in female NSG mice. X-Y) While IBA+ cell numbers did not differ across groups (X), microglial branch length was significantly decreased in aging vehicle-treated female NSG mice and significantly increased by iMP treatment (Y). Z) The percent of LAMP1+ microglia did not differ in female mice. Data are presented as mean (±SEM) values; *n*=10-19/group for male NSG mice (A-V); *n*=8-17/group for female NSG mice (W-Z). * $p < 0.05$, ** $p < 0.01$, *** $p < 0.001$, ns = not significant; one-way ANOVA with Tukey's multiple comparisons test correction.

Supplemental Figure 4

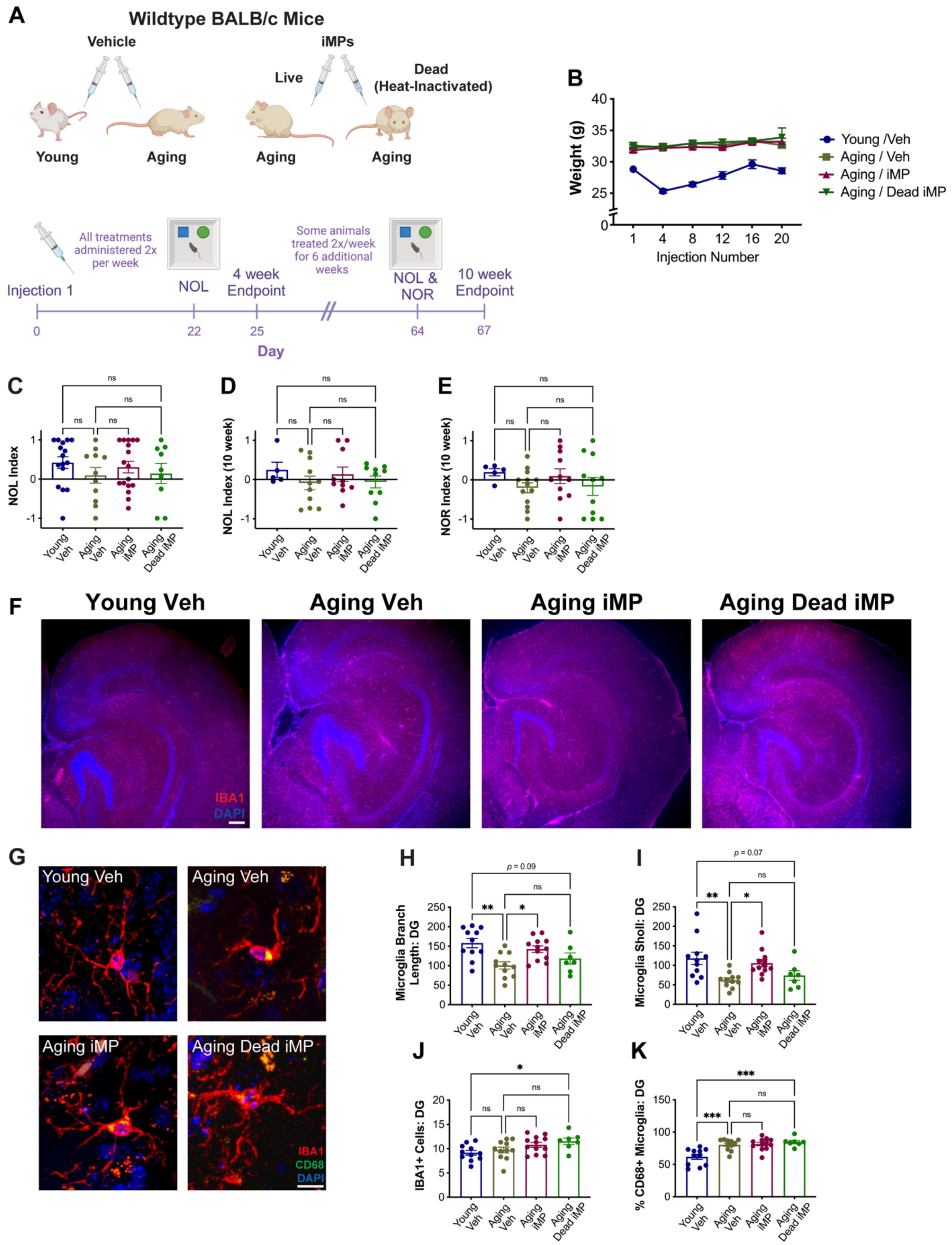

Figure S4. A) Schematic showing male BALB/c mice received vehicle (saline), live iMPs (500,000 cells/ injection), or dead, heat-inactivated iMPs (500,000 cells/injection) by tail vein injection twice per week. Some animals were euthanized after 4 weeks of treatment (8 injections) while others were treated for an additional 6 weeks (20 injections total). Behavior was evaluated at the indicated time points. B) iMP treatment did not significantly affect body weight across time. C-E) BALB/c mice were tested on novel object location (NOL) at 4 and 10 weeks and novel object recognition (NOR) after 10 weeks of treatment. Both tests showed a trend toward

reduced recognition in aging vehicle- and aging dead iMP-treated mice but failed to reach statistical significance. F) Representative images of IBA1 staining in hippocampus of BALB/c mice. Scale bar = 200μm. G) Images showing staining for the microglial marker IBA1 (red) and for lysosomal marker CD68 (green) in dentate gyrus (DG) of the hippocampus. Scale bar = 10μm. H-I) Quantification shows that microglial branch length (H) and complexity as assessed by Sholl analysis (I) were significantly decreased in aging vehicle-treated mice and restored after treatment with live, but not dead iMPs. J) IBA1+ cell numbers are increased specifically in aging dead-iMP treated mice. K) The percent of IBA1+ microglia expressing CD68 was increased across aging groups. Data are presented as mean (±SEM) values; n=9-17/ group for F, and n=7-12/group for H-K. * $p < 0.05$, ** $p < 0.01$, *** $p < 0.001$, ns = not significant; one-way ANOVA with Tukey's multiple comparisons test correction.

Supplemental Figure 5

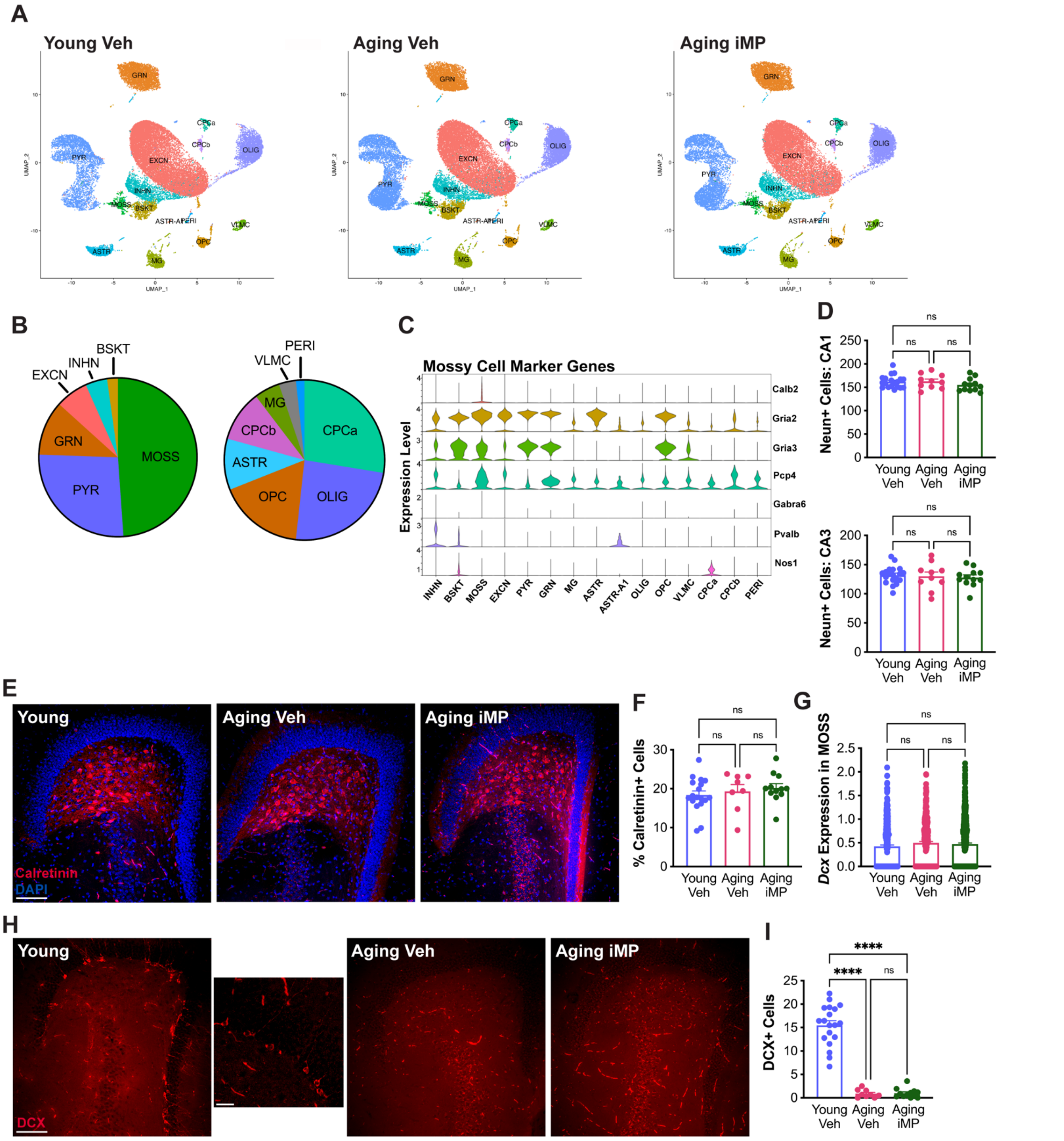

Figure S5. A) Clustering of hippocampal snRNA-seq samples by PCA and UMAP and split by group. B) Differentially expressed genes (DEGs) between young and aging vehicle-treated groups in neuronal cells (top) and in non-neuronal cells (bottom). C) Violin plot showing expression of key marker genes for mossy cells (Calb2, Gria2, Gria3, and Pcp4) and lack of expression of granule cell marker Gabra6 and inhibitory neuron markers Pvalb and Nos1. D) NeuN+ cell numbers are not affected by aging or treatment in either hippocampal CA1 or CA3. E) Images of staining for the mossy cell marker, calretinin, in hippocampal dentate gyrus. Scale bar = 100μm. F) The number of calretinin+ cells does not vary across groups. G) DCX gene expression, shown as

normalized counts, does not differ across groups. H) Images of staining for the newborn neuron marker, DCX. Scale bar = 100μm on left image and 25μm on magnified inset. I) The number of DCX+ cells in dentate gyrus is significantly decreased in both aging groups. Data are presented as mean (±SEM) values; *n*=10-19/group. **** $p < 0.0001$, ns = not significant; one-way ANOVA with Tukey's multiple comparisons test correction.

Supplemental Figure 6

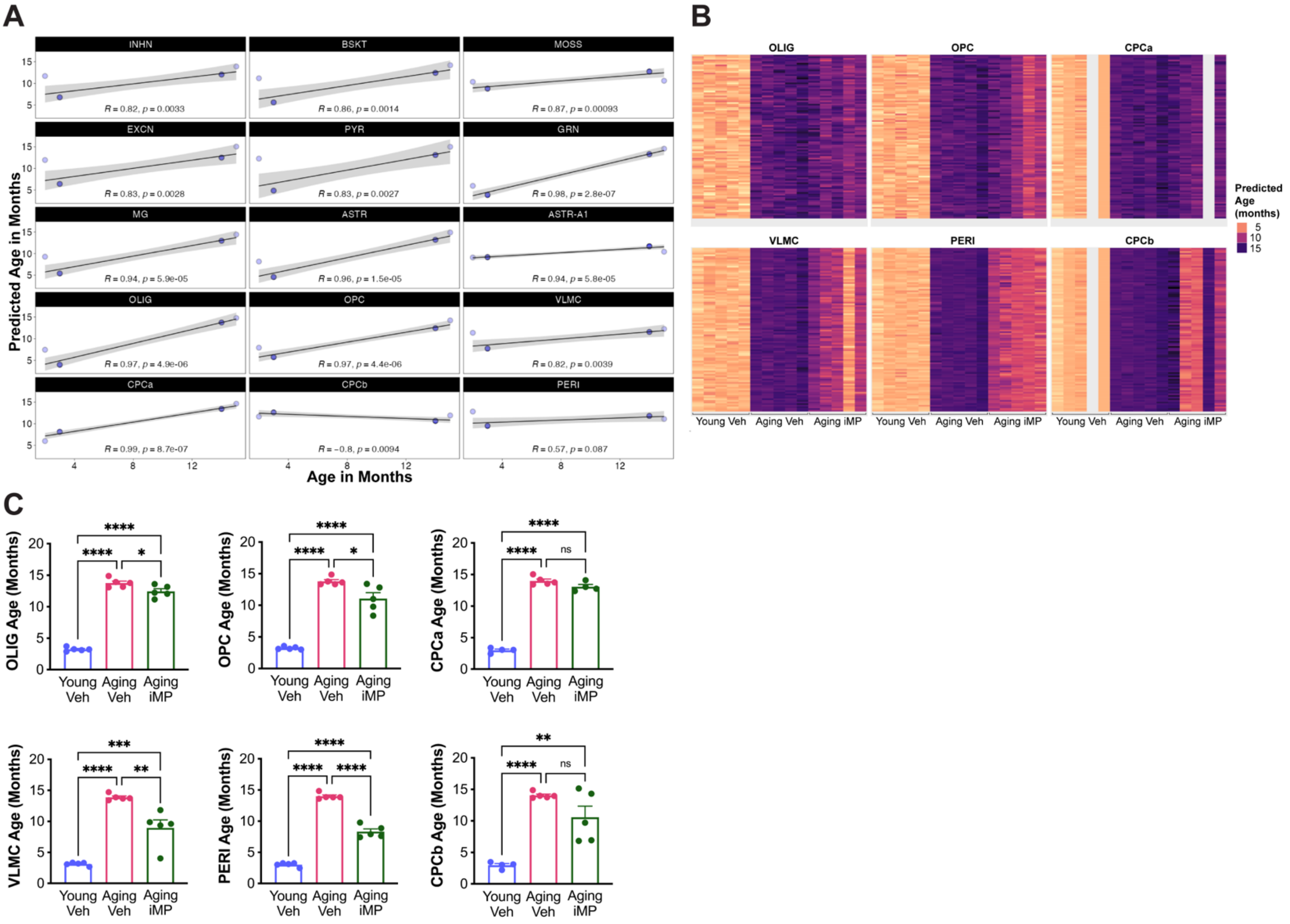

Figure S6. A) Cross-validation of the single-cell chronological clock method for the prediction of ages across cell types. One young and one aging vehicle animal were randomly selected for leave-one-out cross validation. A model was trained on the remaining samples and used to predict the held-out samples' ages. This was repeated five times so each sample was held out once. n=10 (5 young and 5 aging vehicle-treated). R = Pearson correlation coefficient. B) Heatmaps showing the predicted ages in months of six hippocampal cell types from each sample. C) iMP treatment significantly reduced the predicted age of several hippocampal cell types including oligodendrocytes (OLIG), oligodendrocyte precursor cells (OPC), vascular leptomeningeal cells (VLMCs), and pericytes (PERI), as compared to aging vehicle-treated cells. Data are presented as mean (±SEM) values; *n*=5/group. * $p < 0.05$, ** $p < 0.01$, *** $p < 0.001$, **** $p < 0.0001$, ns = not significant; one-way ANOVA with Tukey's multiple comparisons test correction.

Supplemental Figure 7

A

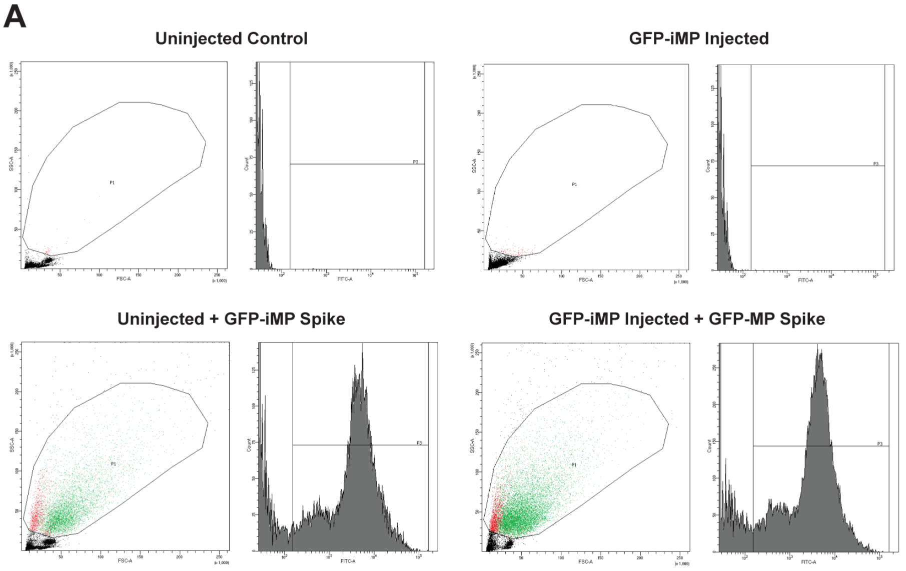

B

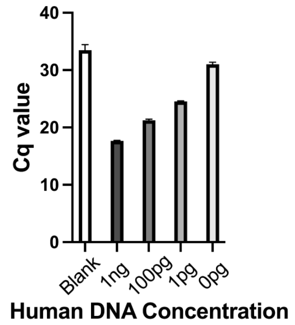

C

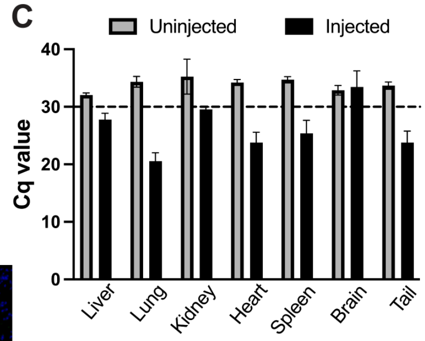

D

Condition	Liver	Lung	Kidney	Heart	Spleen
Aging Veh	-	-	-	-	-
Aging Veh	-	-	-	-	-
Aging Veh	-	-	-	-	-
Aging Veh	-	-	-	-	-
Aging Veh	-	-	-	-	-
Aging iMP	-	+	-	-	-
Aging iMP	-	+	+	-	-
Aging iMP	-	+	-	-	+
Aging iMP	-	-	-	-	-
Aging iMP	-	+	-	-	-
Aging iMP	-	-	-	+	+

E

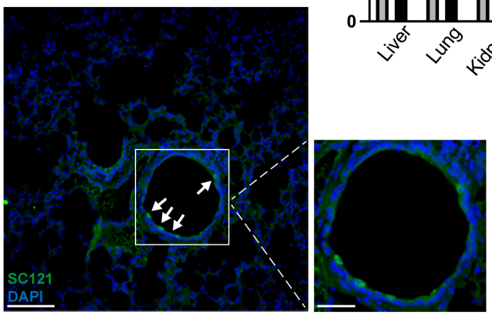

F

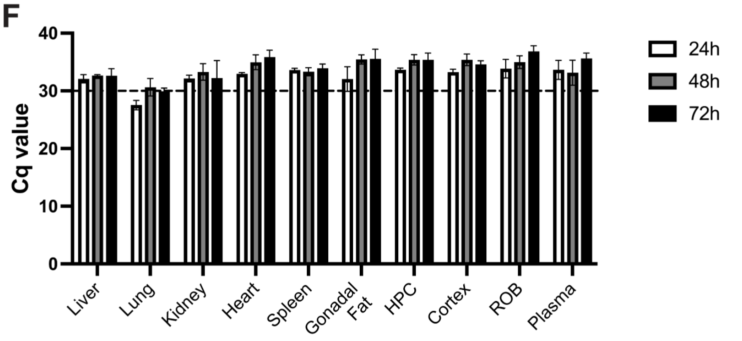

G

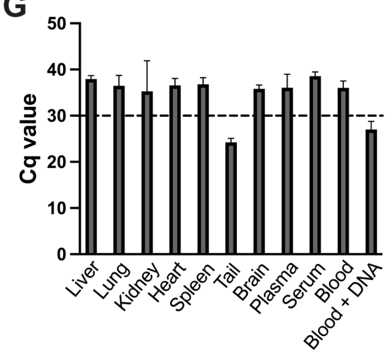

H

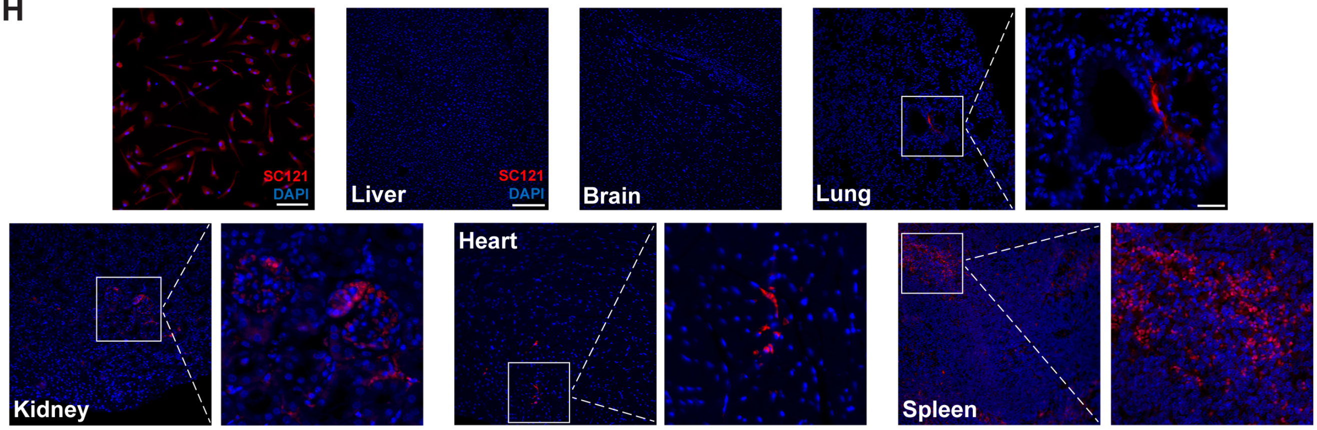

Figure S7. A) Flow cytometry analysis on blood from an uninjected control mouse (left panels) and from a mouse that received $12x10^6$ GFP-labeled iMPs via tail vein administration (right panels) 30 min prior to sacrifice. No GFP+ cells are detected in either animal (top panels) but are readily detectable when blood is spiked with GFP+ iMPs immediately after collection (bottom panels). B) Human DNA diluted to as little as 1pg in mouse DNA is detectable by PCR for the human specific Alu elements. C) PCR targeting human Alu repeats in tissues from uninjected mice (grey) show no detectable human DNA, whereas in mice injected with $12x10^6$ iMPs (black), human DNA is found in all tissues with the exception of the brain. Dashed lines show detection threshold; samples with Cq values below 30 are considered positive. D) Results of PCR targeting human Alu repeats in tissues from a subset of experimental animals that received vehicle or 500,000 iMPs every third day. E) Staining for the human-specific marker, SC121 (green), in lung tissue from an experimental animal treated with 500,000 iMPs every third day. Scale bar = 100μm on left image and 50μm on right image. F) PCR targeting human Alu repeats in tissues collected from mice 24, 48, or 72h post-treatment with a dose of $5x10^6$ iMPs shows that human DNA is detected in lung tissue for up to 72h. HPC = hippocampus; ROB = rest of brain. G) Genomic DNA was isolated from $12x10^6$ iMPs, administered via tail vein injection, and tissues were harvested 30 min later. Human Alu repeats were found in tail and in blood spiked with DNA, but not in any other tissue. H) Findings from human Alu repeat PCR were confirmed by SC121 staining (red) in cultured iMPs and in animals treated with a single dose of $3x10^6$ iMPs. Scale bar = 100μm for cultured iMP image, 200μm for lower magnification images, and 50μm for higher magnification insets.

Supplemental Figure 8

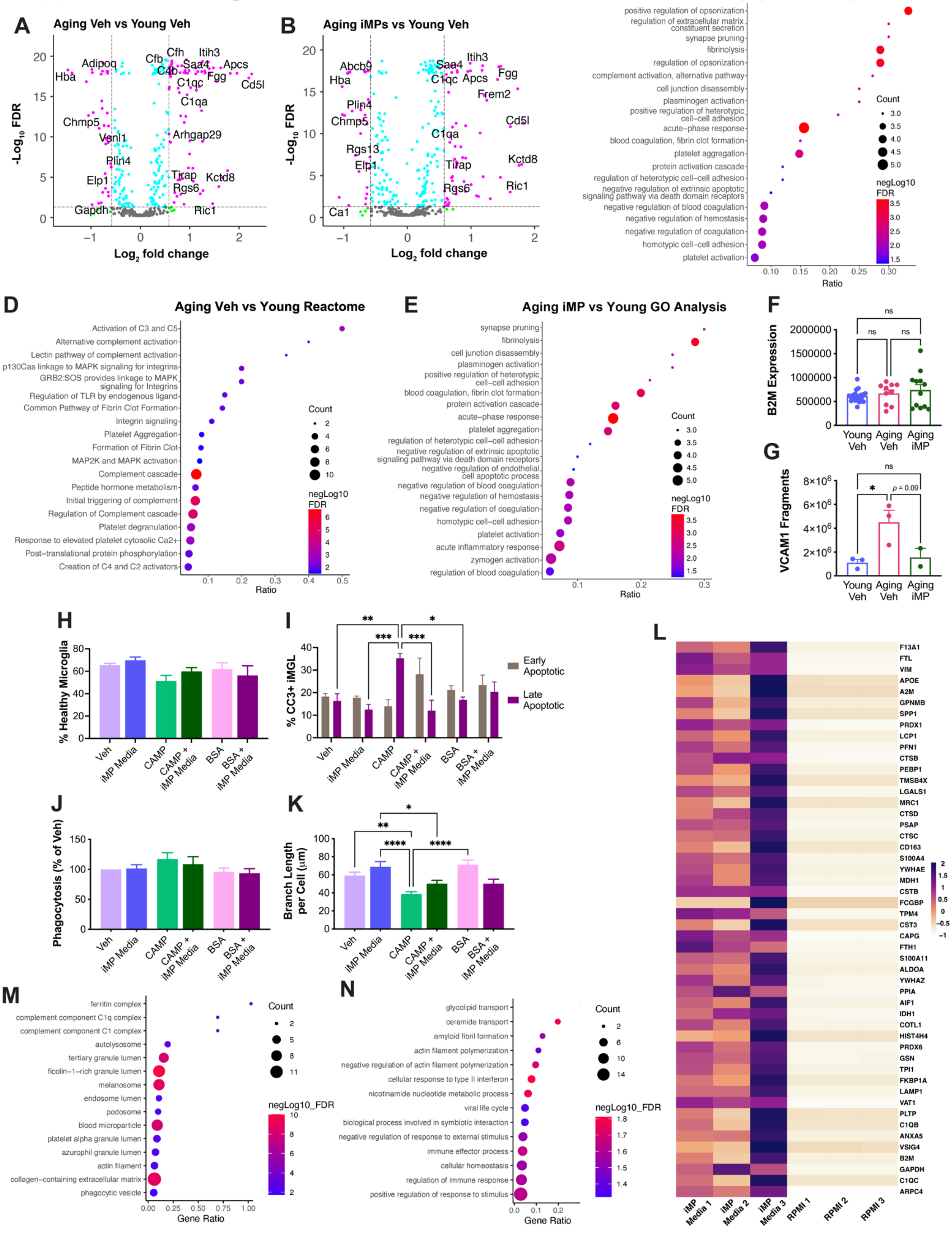

Figure S8. A) Proteins differentially expressed between aging vehicle-treated and young mice at FDR < 0.05 and at a fold change of 1.5 or greater are shown in a volcano plot. One protein haptoglobin (Hp) was increased >5-fold and is outside the range of the volcano plot. B) Proteins differentially expressed between iMP- and vehicle-treated aging mice. Hp was increased >4-fold and is outside the range of the volcano plot. C-D) The 75 proteins that were significantly increased in aging vehicle-treated as compared to young mice were analyzed by (C) biological process gene ontology (GO) analysis and (D) reactome pathway analysis. E) Biological process GO analysis on the 63 proteins that were significantly upregulated in aging iMP-treated as compared to young mice. For C-E, the size of dots reflects the number of proteins within a term that are differentially expressed between groups, while the color reflects the -10log FDR. F) Levels of B2M did not differ across groups. Data are presented as mean (±SEM) values; *n*=10-20/group. ns = not significant; pair-wise comparisons with imputed FDR shown. G) Levels of vascular adhesion molecule 1 (VCAM1), assayed by PRM, are significantly increased in aging, with a trend towards decreased expression after iMP treatment. Data are presented as mean (±SEM) values; *n*=2-3/group. * $p < 0.05$, ns = not significant; one-way ANOVA with Tukey's multiple comparisons test correction. H-I) iMGL treated with 20μM camptothecin (CAMP) or 40 ng/mL bovine serum albumin (BSA) either in RPMI media (Veh) or in iMP-conditioned media. CAMP significantly increased the percent of iMGL in late-stage apoptosis, while iMP-conditioned media reversed the effect of CAMP. J) iMGL phagocytosis did not differ across treatments. K) iMGL treated with CAMP had a significant reduction in branch length, which did not reach statistical significance in the presence of iMP-conditioned media. Data are presented as mean (±SEM) values; *n*=3-5 independent wells/group. * $p < 0.05$, ** $p < 0.01$, *** $p < 0.001$, **** $p < 0.0001$, ns = not significant; one-way ANOVA with Tukey's multiple comparisons test correction. L) Heatmap of the top 50 proteins found in conditioned iMP media. M-N) GO analyses for cellular component (M) and for biological processes (N), on the top 50 iMP conditioned media proteins.

Supplemental Figure 9

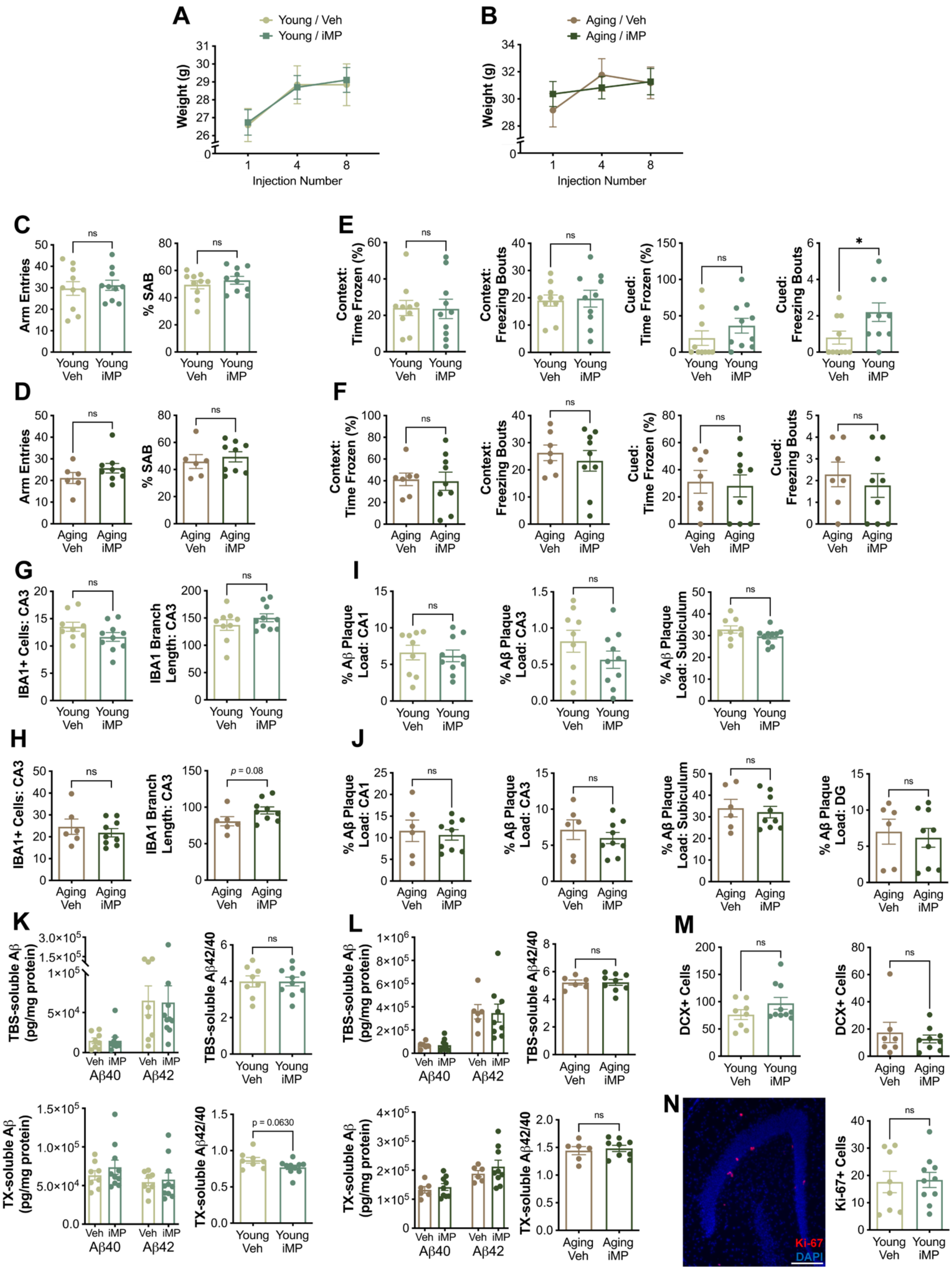

Figure S9. A-B) Body weight did not differ by treatment in (A) young or (B) aging 5xFAD mice. C-D) iMP treatment had no effect on spontaneous alternation behavior and total arm entries in the Y maze in either (C) young or (D) aging 5xFAD mice. E-F) Cued and contextual fear conditioning. There was no effect of iMP treatment on contextual fear conditioning in young or aging 5xFAD mice. However, iMP treatment significantly increased freezing bouts in cued fear conditioning specifically in young 5xFAD mice (E, right panel). G-H) The total number of IBA+ cells in hippocampal CA3 did not differ by iMP treatment in young or aging mice. There was no effect of treatment on CA3 microglial branch length in young mice (G), however, there was a non-significant trend towards increased branch length in aging mice (H, right panel). I-J) Amyloid-β (Aβ) plaque load did not differ between vehicle- or iMP-treated mice in either age across hippocampal subregions. K-L) TBS-soluble and detergent (Triton X)-soluble levels of Aβ40 and Aβ42 did not differ in either young or aging 5xFAD mice. However, there was a nonsignificant trend towards a reduction in the TX-soluble Aβ42/40 ratio, specifically in young 5xFAD mice (K, bottom right panel). M) Neurogenesis, as assessed by quantification of the newborn neuron marker, DCX, did not differ with iMP treatment in either young or aging 5xFAD mice. N) Staining and quantification of the cell proliferation marker, Ki67 (red). Scale bar = 100μm. There was no effect of iMP treatment on Ki-67+ cell number in young 5xFAD mice. Data are presented as mean (±SEM) values; *n*=6-10/group. * $p < 0.05$, ns = not significant; unpaired *t*-test.

Table S1 (Supporting Information). Plasma proteins differentially expressed in aging versus young mice and in iMP-treated versus aging mice. A literature search shows the relevance of these proteins in Alzheimer's disease, inflammation, aging, and cognition.

**Increased in aging vehicle vs. young vehicle**					
**Protein**	**Alzheimer's**	**Inflammation**	**Aging**	**Cognition**	**References**
Hp	X	X	X		(Schaer et al., 2014); (Philbert et al., 2021); (Krzyszczyk et al., 2020); (Ding and Kopchick, 2011)
Znrd1-as					
Cd5l	X	X			(Sanjurjo et al., 2018); (Fang et al., 2022)
Serpina7					
Frem2	X				
Kctd8				X	(Teng et al., 2019); (Muntean et al., 2022)
Srrm3					
Apcs	X	X			(Tennent et al., 1995);(Pepys et al., 2002); (Bodin et al., 2010); (Doni et al., 2021)
Efhc1					
Cables1			X		(Pu et al., 2017); (He et al., 2019)
Fgg	X				(Ashton et al., 2015)
Ric1					
Rnase4	X				(Preman et al., 2021)
Fga	X				(Bian et al., 2021); (Kim et al., 2022)
Itih3	X				(Meier et al., 2015)
Znf704			X		(Goodall et al., 2019)
Orm1	X	X			(Kim et al., 2022); (Lee et al., 2010)
Csn2					
Hpx	X				(Ashraf et al., 2020)
Egln1	X				(Liu et al., 2022)
Csmd1			X	X	(Stepanov et al., 2017)
Hspa5	X	X			(Hsu et al., 2008); (Xiao et al., 2015)
Itih4	X	X			(Shi et al., 2019); (Ma et al., 2021)
Abcc12					
Pigz	X				(Toyn et al., 2010)
Pla2g7	X	X	X		(van Oijen et al., 2006); (Heath et al., 2022); (Rosenson and Stafforini, 2012); (Spadaro et al., 2022)
Ank2	X			X	(Higham et al., 2019)
Vill					
Fgb	X				(Bian et al., 2021); (Kim et al., 2022)
Rgs6					

Tirap		X			(Rajpoot et al., 2021)
Arsb	X				(Niculescu et al., 2020)
Arhgap29					
Lyz2	X	X			(Keren-Shaul et al., 2017)
Saa4	X	X			(Buck et al., 2016); (Chen and Xia, 2020)
Ruvbl1	X				(Kikuchi et al., 2013)
C1qb	X	X	X		(Lau et al., 2020); (Dejanovic et al., 2022); (Stephan et al., 2013)
C1qa	X	X	X		(Lau et al., 2020); (Dejanovic et al., 2022); (Stephan et al., 2013)
Cp	X		X	X	(Semsei et al., 1993); (Diouf et al., 2020)
Arhgap30	X				(Wörheide et al., 2021)
Wdr53					
Cpb2	X	X			(Visser et al., 2022); (Ray, 2011)
Pla2g4e	X				(Pérez-González et al., 2020)
Dnah12					
C1qc	X	X	X		(Lau et al., 2020); (Dejanovic et al., 2022); (Stephan et al., 2013)
Phtf2					
Ambp	X	X			(Higginbotham et al., 2020)
Nlrp10	X	X			(Murphy et al., 2014)
Spata7					
Flg2	X	X			(Miller et al., 2018)
Exoc2	X				(Vélez et al., 2016)
Ddias					
Bicd1					
Dcst1					
Dhrs1					
Cfp		X			(Blatt et al., 2016)
Ptpn22		X			(Spalinger et al., 2021)
Fcn1		X			(Olah et al., 2020)
Otop3					
Serpina3n	X	X		X	(Zattoni et al., 2022); (Fissolo et al., 2021); (Wang et al., 2020)
Cfh	X	X			(Hye et al., 2006); (Lukiw and Alexandrov, 2012)
C4b	X	X			(Brouwers et al., 2012); (Goetzl et al., 2018)
Cfb		X			(Chen et al., 2010)
Kirrel1					
Pld1	X				(Cai et al., 2006)

Itih2					
Zfyve28					
Ldlrap1	X				(Kong et al., 2009)
Ntn5	X				(Wightman et al., 2020)
Colec11		X			(Bright et al., 2021)
H2-Q10	X	X			(Song et al., 2020); (Zhai et al., 2017)
Agt					
Med131					
Sbno2		X			(Syme et al., 2022)
Ceacam5		X			(Denmark et al., 2011)
**Decreased in aging vehicle vs. young vehicle**					
**Protein**	**Alzheimer's**	**Inflammation**	**Aging**	**Cognition**	**References**
Plin4					
Psd					
Mug2					
Ctnnd1					
Sorbs3			X		(Park et al., 2022)
Kif5a	X				(Wang et al., 2019)
Ncor2	X			X	(Shigemizu et al., 2022); (Zhou et al., 2019)
Pabir2					
Ccdc150					
Nphs2					
Itga9	X				(Shulman et al., 2014)
Lats1					
Vsnl1	X		X		(Lee et al., 2008); (Lin et al., 2015)
Gmnn					
Mis18bp1				X	(Tan et al., 2019)
Elp1					
Nek7		X			(Sun et al., 2020)
Gsn	X				(Peng et al., 2015)
Cd2ap	X				(Qing-Qing et al., 2018)
Lum					
Nt5dc1					
Abcb9	X	X			(Nazarian et al., 2019); (Szakacs and Abele, 2020)
Blvrb	X				(Haure-Mirande et al., 2017); (Mueller et al., 2010)

Mroh2a					
Rgs13		X			(Squires et al., 2018)
Gapdh	X		X		(Lazarev et al., 2021); (Vigelsø et al., 2015)
Wdr64					
Acss3					
Fbxw11	X	X			(Sun et al., 2021)
Adipoq	X	X			(Uddin et al., 2020)
Chmp5		X			(Greenblatt et al., 2015)
Ryr1					
Hbb-b1					
Hba					
**Increased in aging iMP vs. aging vehicle**					
**Protein**	**Alzheimer's**	**Inflammation**	**Aging**	**Cognition**	**References**
Adipoq	X	X			(Uddin et al., 2020)
Mtus1	X				(Chung et al., 2018)
Gapdh	X		X		(Lazarev et al., 2021); (Vigelsø et al., 2015)
**Decreased in aging iMP vs. aging vehicle**					
**Protein**	**Alzheimer's**	**Inflammation**	**Aging**	**Cognition**	**References**
Saa2	X	X	X	X	(Chung et al., 2018); (Guo et al., 2002); (Jensen and Whitehead, 1998); (Rosenthal and Franklin, 1975); (Xu et al., 2021)
Ppp1r21	X				(Boza-Serrano et al., 2018)
Apcs	X	X			(Tennent et al., 1995);(Pepys et al., 2002); (Bodin et al., 2010); (Doni et al., 2021)
Arhgap					

References

1. Schaer, D. J., Vinchi, F., Ingoglia, G., Tolosano, E. & Buehler, P. W. Haptoglobin, hemopexin, and related defense pathways-basic science, clinical perspectives, and drug development. *Front. Physiol.* **5**, 415 (2014).
2. Philbert, S. A., Xu, J., Unwin, R. D., Dowsey, A. W. & Cooper, G. J. S. Widespread severe cerebral elevations of haptoglobin and haemopexin in sporadic Alzheimer's disease: Evidence for a pervasive microvasculopathy. *Biochem. Biophys. Res. Commun.* **555**, 89–94 (2021).
3. Krzyszczyk, P. *et al.* Anti-inflammatory effects of haptoglobin on LPS-stimulated macrophages: Role of HMGB1 signaling and implications in chronic wound healing. *Wound Repair Regen.* **28**, 493–505 (2020).
4. Ding, J. & Kopchick, J. J. Plasma biomarkers of mouse aging. *Age (Dordr)* **33**, 291–307 (2011).
5. Sanjurjo, L. *et al.* CD5L Promotes M2 Macrophage Polarization through Autophagy-Mediated Upregulation of ID3. *Front. Immunol.* **9**, 480 (2018).
6. Fang, Y. *et al.* Association between inflammatory biomarkers and cognitive aging. *PLoS ONE* **17**, e0274350 (2022).
7. Teng, X. *et al.* KCTD: A new gene family involved in neurodevelopmental and neuropsychiatric disorders. *CNS Neurosci. Ther.* **25**, 887–902 (2019).
8. Muntean, B. S. *et al.* Members of the KCTD family are major regulators of cAMP signaling. *Proc Natl Acad Sci USA* **119**, (2022).
9. Tennent, G. A., Lovat, L. B. & Pepys, M. B. Serum amyloid P component prevents proteolysis of the amyloid fibrils of Alzheimer disease and systemic amyloidosis. *Proc Natl Acad Sci USA* **92**, 4299–4303 (1995).
10. Pepys, M. B. *et al.* Targeted pharmacological depletion of serum amyloid P component for treatment of human amyloidosis. *Nature* **417**, 254–259 (2002).
11. Bodin, K. *et al.* Antibodies to human serum amyloid P component eliminate visceral amyloid deposits. *Nature* **468**, 93–97 (2010).
12. Doni, A. *et al.* Serum amyloid P component is an essential element of resistance against Aspergillus fumigatus. *Nat. Commun.* **12**, 3739 (2021).
13. Pu, Z. *et al.* Cables1 Inhibits Proliferation and Induces Senescence by Angiotensin II via a p21-Dependent Pathway in Human Umbilical Vein Endothelial Cells. *J. Vasc. Res.* **54**, 13–21 (2017).
14. He, L. *et al.* CABLES1 deficiency impairs quiescence and stress responses of hematopoietic stem cells in intrinsic and extrinsic manners. *Stem Cell Reports* **13**, 274–290 (2019).
15. Ashton, N. J. *et al.* Blood protein predictors of brain amyloid for enrichment in clinical trials? *Alzheimers Dement (Amst)* **1**, 48–60 (2015).
16. Preman, P., Alfonso-Triguero, M., Alberdi, E., Verkhratsky, A. & Arranz, A. M. Astrocytes in alzheimer's disease: pathological significance and molecular pathways. *Cells* **10**, (2021).
17. Bian, Z. *et al.* Accelerated accumulation of fibrinogen peptide chains with Aβ deposition in Alzheimer's disease (AD) mice and human AD brains. *Brain Res.* **1767**, 147569 (2021).

18. Kim, Y. *et al.* Plasma protein biomarker model for screening Alzheimer disease using multiple reaction monitoring-mass spectrometry. *Sci. Rep.* **12**, 1282 (2022).
19. Meier, S. *et al.* Identification of Novel Tau Interactions with Endoplasmic Reticulum Proteins in Alzheimer's Disease Brain. *J Alzheimers Dis* **48**, 687–702 (2015).
20. Goodall, E. F. *et al.* Age-Associated mRNA and miRNA Expression Changes in the Blood-Brain Barrier. *Int. J. Mol. Sci.* **20**, (2019).
21. Lee, Y. S. *et al.* Adipocytokine orosomucoid integrates inflammatory and metabolic signals to preserve energy homeostasis by resolving immoderate inflammation. *J. Biol. Chem.* **285**, 22174–22185 (2010).
22. Ashraf, A. A., Dani, M. & So, P.-W. Low cerebrospinal fluid levels of hemopexin are associated with increased alzheimer's pathology, hippocampal hypometabolism, and cognitive decline. *Front. Mol. Biosci.* **7**, 590979 (2020).
23. Liu, H. *et al.* Exploring links between 2-oxoglutarate-dependent oxygenases and Alzheimer's disease. *Alzheimers Dement* (2022) doi:10.1002/alz.12733.
24. Stepanov, V., Marusin, A., Vagaitseva, K., Bocharova, A. & Makeeva, O. Genetic Variants in CSMD1 Gene Are Associated with Cognitive Performance in Normal Elderly Population. *Genet. Res. Int.* **2017**, 6293826 (2017).
25. Hsu, W. C. *et al.* Promoter polymorphisms modulating HSPA5 expression may increase susceptibility to Taiwanese Alzheimer's disease. *J. Neural Transm.* **115**, 1537–1543 (2008).
26. Xiao, F., Tan, J.-Z., Xu, X.-Y. & Wang, X.-F. Increased levels of HSPA5 in the serum of patients with inflammatory myopathies--preliminary findings. *Clin. Rheumatol.* **34**, 715–720 (2015).
27. Shi, X. *et al.* Acute Anti-Inflammatory Markers ITIH4 and AHSG in Mice Brain of a Novel Alzheimer's Disease Model. *J Alzheimers Dis* **68**, 1667–1675 (2019).
28. Ma, Y. *et al.* ITIH4, as an inflammation biomarker, mainly increases in bacterial bloodstream infection. *Cytokine* **138**, 155377 (2021).
29. Toyn, J. H. *et al.* Viable mouse gene ablations that robustly alter brain Aβ levels are rare. *BMC Neurosci.* **11**, 143 (2010).
30. van Oijen, M. *et al.* Lipoprotein-associated phospholipase A2 is associated with risk of dementia. *Ann. Neurol.* **59**, 139–144 (2006).
31. Heath, L. *et al.* Manifestations of Alzheimer's disease genetic risk in the blood are evident in a multiomic analysis in healthy adults aged 18 to 90. *Sci. Rep.* **12**, 6117 (2022).
32. Rosenson, R. S. & Stafforini, D. M. Modulation of oxidative stress, inflammation, and atherosclerosis by lipoprotein-associated phospholipase A2. *J. Lipid Res.* **53**, 1767–1782 (2012).
33. Spadaro, O. *et al.* Caloric restriction in humans reveals immunometabolic regulators of health span. *Science* **375**, 671–677 (2022).
34. Higham, J. P. *et al.* Alzheimer's disease associated genes ankyrin and tau cause shortened lifespan and memory loss in drosophila. *Front. Cell. Neurosci.* **13**, 260 (2019).
35. Rajpoot, S. *et al.* TIRAP in the mechanism of inflammation. *Front. Immunol.* **12**, 697588 (2021).
36. Niculescu, A. B. *et al.* Blood biomarkers for memory: toward early detection of risk for Alzheimer disease, pharmacogenomics, and repurposed drugs. *Mol. Psychiatry* **25**, 1651–1672 (2020).

37. Keren-Shaul, H. *et al.* A Unique Microglia Type Associated with Restricting Development of Alzheimer's Disease. *Cell* **169**, 1276-1290.e17 (2017).
38. Buck, M. *et al.* Structure and Expression of Different Serum Amyloid A (SAA) Variants and their Concentration-Dependent Functions During Host Insults. *CMC* **23**, 1725–1755 (2016).
39. Chen, M. & Xia, W. Proteomic Profiling of Plasma and Brain Tissue from Alzheimer's Disease Patients Reveals Candidate Network of Plasma Biomarkers. *J Alzheimers Dis* **76**, 349–368 (2020).
40. Kikuchi, M. *et al.* Identification of unstable network modules reveals disease modules associated with the progression of Alzheimer's disease. *PLoS ONE* **8**, e76162 (2013).
41. Lau, S.-F., Cao, H., Fu, A. K. Y. & Ip, N. Y. Single-nucleus transcriptome analysis reveals dysregulation of angiogenic endothelial cells and neuroprotective glia in Alzheimer's disease. *Proc Natl Acad Sci USA* **117**, 25800–25809 (2020).
42. Dejanovic, B. *et al.* Complement C1q-dependent excitatory and inhibitory synapse elimination by astrocytes and microglia in Alzheimer's disease mouse models. *Nat. Aging* (2022) doi:10.1038/s43587-022-00281-1.
43. Stephan, A. H. *et al.* A dramatic increase of C1q protein in the CNS during normal aging. *J. Neurosci.* **33**, 13460–13474 (2013).
44. Semsei, I., Jeney, F. & Fülöp, T. Effect of age on the activity of ceruloplasmin of human blood. *Arch. Gerontol. Geriatr.* **17**, 123–130 (1993).
45. Diouf, I., Bush, A. I., Ayton, S. & Alzheimer's disease Neuroimaging Initiative. Cerebrospinal fluid ceruloplasmin levels predict cognitive decline and brain atrophy in people with underlying β-amyloid pathology. *Neurobiol. Dis.* **139**, 104810 (2020).
46. Wörheide, M. A. *et al.* An integrated molecular atlas of alzheimer's disease. *medRxiv* (2021) doi:10.1101/2021.09.14.21263565.
47. Visser, P. J. *et al.* Cerebrospinal fluid tau levels are associated with abnormal neuronal plasticity markers in Alzheimer's disease. *Mol. Neurodegener.* **17**, 27 (2022).
48. Ray, K. CPB2 dampens inflammation in autoimmune arthritis. *Nat. Rev. Rheumatol.* **7**, 558 (2011).
49. Pérez-González, M. *et al.* PLA2G4E, a candidate gene for resilience in Alzheimer´s disease and a new target for dementia treatment. *Prog. Neurobiol.* **191**, 101818 (2020).
50. Higginbotham, L. *et al.* Integrated proteomics reveals brain-based cerebrospinal fluid biomarkers in asymptomatic and symptomatic Alzheimer's disease. *Sci. Adv.* **6**, (2020).
51. Murphy, N., Grehan, B. & Lynch, M. A. Glial uptake of amyloid beta induces NLRP3 inflammasome formation via cathepsin-dependent degradation of NLRP10. *Neuromolecular Med.* **16**, 205–215 (2014).
52. Miller, J. E. *et al.* Codon bias among synonymous rare variants is associated with Alzheimer's disease imaging biomarker. *Pac. Symp. Biocomput.* **23**, 365–376 (2018).
53. Vélez, J. I. *et al.* A mutation in DAOA modifies the age of onset in PSEN1 E280A alzheimer's disease. *Neural Plast.* **2016**, 9760314 (2016).
54. Blatt, A. Z., Pathan, S. & Ferreira, V. P. Properdin: a tightly regulated critical inflammatory modulator. *Immunol. Rev.* **274**, 172–190 (2016).

55. Spalinger, M. R. *et al.* Loss of PTPN22 Promotes Intestinal Inflammation by Compromising Granulocyte-mediated Antibacterial Defence. *J Crohns Colitis* **15**, 2118–2130 (2021).
56. Olah, M. *et al.* Single cell RNA sequencing of human microglia uncovers a subset associated with Alzheimer's disease. *Nat. Commun.* **11**, 6129 (2020).
57. Zattoni, M. *et al.* Serpin signatures in prion and alzheimer's diseases. *Mol. Neurobiol.* **59**, 3778–3799 (2022).
58. Fissolo, N. *et al.* CSF SERPINA3 levels are elevated in patients with progressive MS. *Neurol. Neuroimmunol. Neuroinflamm.* **8**, (2021).
59. Wang, Z.-M. *et al.* SerpinA3N deficiency deteriorates impairments of learning and memory in mice following hippocampal stab injury. *Cell Death Discov.* **6**, 88 (2020).
60. Hye, A. *et al.* Proteome-based plasma biomarkers for Alzheimer's disease. *Brain* **129**, 3042–3050 (2006).
61. Lukiw, W. J. & Alexandrov, P. N. Regulation of complement factor H (CFH) by multiple miRNAs in Alzheimer's disease (AD) brain. *Mol. Neurobiol.* **46**, 11–19 (2012).
62. Brouwers, N. *et al.* Alzheimer risk associated with a copy number variation in the complement receptor 1 increasing C3b/C4b binding sites. *Mol. Psychiatry* **17**, 223–233 (2012).
63. Goetzl, E. J., Schwartz, J. B., Abner, E. L., Jicha, G. A. & Kapogiannis, D. High complement levels in astrocyte-derived exosomes of Alzheimer disease. *Ann. Neurol.* **83**, 544–552 (2018).
64. Chen, M., Muckersie, E., Luo, C., Forrester, J. V. & Xu, H. Inhibition of the alternative pathway of complement activation reduces inflammation in experimental autoimmune uveoretinitis. *Eur. J. Immunol.* **40**, 2870–2881 (2010).
65. Cai, D. *et al.* Phospholipase D1 corrects impaired betaAPP trafficking and neurite outgrowth in familial Alzheimer's disease-linked presenilin-1 mutant neurons. *Proc Natl Acad Sci USA* **103**, 1936–1940 (2006).
66. Kong, W. *et al.* Independent component analysis of Alzheimer's DNA microarray gene expression data. *Mol. Neurodegener.* **4**, 5 (2009).
67. Wightman, D. P. *et al.* Largest GWAS (N=1,126,563) of alzheimer's disease implicates microglia and immune cells. *medRxiv* (2020) doi:10.1101/2020.11.20.20235275.
68. Bright, F. *et al.* Glycoprotein pathways altered in frontotemporal dementia with autoimmune disease. *Front. Immunol.* **12**, 736260 (2021).
69. Song, Z. *et al.* Comprehensive Proteomic Profiling of Urinary Exosomes and Identification of Potential Non-invasive Early Biomarkers of Alzheimer's Disease in 5XFAD Mouse Model. *Front. Genet.* **11**, 565479 (2020).
70. Zhai, J., Bo, Y., Lu, Y., Liu, C. & Zhang, L. Effects of Coenzyme Q10 on Markers of Inflammation: A Systematic Review and Meta-Analysis. *PLoS ONE* **12**, e0170172 (2017).
71. Syme, T. E. *et al.* Strawberry notch homolog 2 regulates the response to interleukin-6 in the central nervous system. *J. Neuroinflammation* **19**, 126 (2022).
72. Denmark, V., Xu, J., Dahan, S. & Mayer, L. CEACAM5 is upregulated with inflammation in Crohn's colitis in response to IL-22. *Inflammatory bowel diseases* **17**, S73–S74 (2011).
73. Park, S. J. *et al.* Vinexin contributes to autophagic decline in brain ageing across species. *Cell Death Differ.* **29**, 1055–1070 (2022).

74. Wang, Q., Tian, J., Chen, H., Du, H. & Guo, L. Amyloid beta-mediated KIF5A deficiency disrupts anterograde axonal mitochondrial movement. *Neurobiol. Dis.* **127**, 410–418 (2019).
75. Shigemizu, D. *et al.* Whole-genome sequencing reveals novel ethnicity-specific rare variants associated with Alzheimer's disease. *Mol. Psychiatry* **27**, 2554–2562 (2022).
76. Zhou, W. *et al.* Loss of function of NCOR1 and NCOR2 impairs memory through a novel GABAergic hypothalamus-CA3 projection. *Nat. Neurosci.* **22**, 205–217 (2019).
77. Shulman, J. M. *et al.* Functional screening in Drosophila identifies Alzheimer's disease susceptibility genes and implicates Tau-mediated mechanisms. *Hum. Mol. Genet.* **23**, 870–877 (2014).
78. Lee, J.-M. *et al.* The brain injury biomarker VLP-1 is increased in the cerebrospinal fluid of Alzheimer disease patients. *Clin. Chem.* **54**, 1617–1623 (2008).
79. Lin, C.-W. *et al.* VSNL1 Co-Expression Networks in Aging Include Calcium Signaling, Synaptic Plasticity, and Alzheimer's Disease Pathways. *Front. Psychiatry* **6**, 30 (2015).
80. Tan, P. K., Ananyev, E. & Hsieh, P.-J. Distinct Genetic Signatures of Cortical and Subcortical Regions Associated with Human Memory. *eNeuro* **6**, (2019).
81. Sun, Z., Gong, W., Zhang, Y. & Jia, Z. Physiological and pathological roles of mammalian NEK7. *Front. Physiol.* **11**, 606996 (2020).
82. Peng, M., Jia, J. & Qin, W. Plasma gelsolin and matrix metalloproteinase 3 as potential biomarkers for Alzheimer disease. *Neurosci. Lett.* **595**, 116–121 (2015).
83. Qing-Qing, T., Yu-Chao, C. & Zhi-Ying, W. The role of CD2AP in the Pathogenesis of Alzheimer's Disease. *Aging Dis.* (2018) doi:10.14336/AD.2018.1025.
84. Nazarian, A., Yashin, A. I. & Kulminski, A. M. Genome-wide analysis of genetic predisposition to Alzheimer's disease and related sex disparities. *Alzheimers Res Ther* **11**, 5 (2019).
85. Szakacs, G. & Abele, R. An inventory of lysosomal ABC transporters. *FEBS Lett.* **594**, 3965–3985 (2020).
86. Haure-Mirande, J.-V. *et al.* Deficiency of TYROBP, an adapter protein for TREM2 and CR3 receptors, is neuroprotective in a mouse model of early Alzheimer's pathology. *Acta Neuropathol.* **134**, 769–788 (2017).
87. Mueller, C. *et al.* The heme degradation pathway is a promising serum biomarker source for the early detection of Alzheimer's disease. *J Alzheimers Dis* **19**, 1081–1091 (2010).
88. Squires, K. E., Montañez-Miranda, C., Pandya, R. R., Torres, M. P. & Hepler, J. R. Genetic analysis of rare human variants of regulators of G protein signaling proteins and their role in human physiology and disease. *Pharmacol. Rev.* **70**, 446–474 (2018).
89. Lazarev, V. F. *et al.* Extracellular GAPDH Promotes Alzheimer Disease Progression by Enhancing Amyloid-β Aggregation and Cytotoxicity. *Aging Dis.* **12**, 1223–1237 (2021).
90. Vigelsø, A. *et al.* GAPDH and β-actin protein decreases with aging, making Stain-Free technology a superior loading control in Western blotting of human skeletal muscle. *J. Appl. Physiol.* **118**, 386–394 (2015).
91. Sun, J. *et al.* FBXW11 deletion alleviates Alzheimer's disease by reducing neuroinflammation and amyloid-β plaque formation via repression of ASK1 signaling. *Biochem. Biophys. Res. Commun.* **548**, 104–111 (2021).

92. Uddin, M. S. *et al.* Exploring the New Horizon of AdipoQ in Obesity-Related Alzheimer's Dementia. *Front. Physiol.* **11**, 567678 (2020).
93. Greenblatt, M. B. *et al.* CHMP5 controls bone turnover rates by dampening NF-κB activity in osteoclasts. *J. Exp. Med.* **212**, 1283–1301 (2015).
94. Chung, J. *et al.* Genome-wide association study of Alzheimer's disease endophenotypes at prediagnosis stages. *Alzheimers Dement* **14**, 623–633 (2018).
95. Guo, J., Yu, J., Grass, D., de Beer, F. C. & Kindy, M. S. Inflammation-Dependent Cerebral Deposition of Serum Amyloid A Protein in a Mouse Model of Amyloidosis. *J. Neurosci.* **22**, 5900–5909 (2002).
96. Jensen, L. E. & Whitehead, A. S. Regulation of serum amyloid A protein expression during the acute-phase response. *Biochem. J.* **334 (Pt 3)**, 489–503 (1998).
97. Rosenthal, C. J. & Franklin, E. C. Variation with age and disease of an amyloid A protein-related serum component. *J. Clin. Invest.* **55**, 746–753 (1975).
98. Xu, M., He, X.-Y. & Huang, P. The Relationship between Serum Amyloid A Level and Cognitive Dysfunction in Patients with Vascular Dementia: Preliminary Findings. *Biomed Res. Int.* **2021**, 6676144 (2021).
99. Boza-Serrano, A., Yang, Y., Paulus, A. & Deierborg, T. Innate immune alterations are elicited in microglial cells before plaque deposition in the Alzheimer's disease mouse model 5xFAD. *Sci. Rep.* **8**, 1550 (2018).

Table S2 (Supporting Information). Gene ontology (GO) and reactome analyses on differentially expressed proteins in plasma.

Up in Aging Veh versus Young Veh_Biological Process

Analysis Type:	PANTHER Overrepresentation Test (Released 20220712)
Annotation Version and Release Date:	GO Ontology database DOI: 10.5281/zenodo.6799722 Released 2022-07-01
Analyzed List:	Client Text Box Input (Mus musculus)
Reference List:	Mus musculus (all genes in database)
Test Type:	FISHER
Correction:	FDR

GO biological process complete	Mus musculus - REFLIST (21997)	Client Text Box Input (77)	Client Text Box Input (expected)	Client Text Box Input (over/under)	Client Text Box Input (fold Enrichment)	Client Text Box Input (raw P-value)	Client Text Box Input (FDR)
positive regulation of opsonization (GO:1903028)	12	4	0.04	+	95.23	2.41E-07	2.72E-04
synapse pruning (GO:0098883)	10	3	0.04	+	85.7	1.14E-05	5.44E-03
regulation of extracellular matrix constituent secretion (GO:0003330)	10	3	0.04	+	85.7	1.14E-05	5.28E-03
regulation of opsonization (GO:1903027)	14	4	0.05	+	81.62	4.03E-07	3.97E-04
fibrinolysis (GO:0042730)	14	4	0.05	+	81.62	4.03E-07	3.74E-04
complement activation, alternative pathway (GO:0006957)	11	3	0.04	+	77.91	1.44E-05	6.33E-03
plasminogen activation (GO:0031639)	12	3	0.04	+	71.42	1.80E-05	6.93E-03
cell junction disassembly (GO:0150146)	12	3	0.04	+	71.42	1.80E-05	6.76E-03
positive regulation of heterotypic cell-cell adhesion (GO:0034116)	14	3	0.05	+	61.22	2.68E-05	9.38E-03
acute-phase response (GO:0006953)	32	5	0.11	+	44.64	1.81E-07	2.38E-04
blood coagulation, fibrin clot formation (GO:0072378)	20	3	0.07	+	42.85	6.87E-05	1.93E-02
platelet aggregation (GO:0070527)	27	4	0.09	+	42.32	4.01E-06	2.63E-03
regulation of heterotypic cell-cell adhesion (GO:0034114)	25	3	0.09	+	34.28	1.26E-04	3.24E-02
protein activation cascade (GO:0072376)	25	3	0.09	+	34.28	1.26E-04	3.19E-02
negative regulation of extrinsic apoptotic signaling pathway via death domain receptors (GO:190204[illegible]	30	3	0.11	+	28.57	2.06E-04	4.65E-02
negative regulation of blood coagulation (GO:0030195)	45	4	0.16	+	25.39	2.57E-05	9.22E-03
negative regulation of hemostasis (GO:1900047)	46	4	0.16	+	24.84	2.79E-05	9.56E-03
homotypic cell-cell adhesion (GO:0034109)	47	4	0.16	+	24.31	3.02E-05	1.01E-02
negative regulation of coagulation (GO:0050819)	47	4	0.16	+	24.31	3.02E-05	9.92E-03
platelet activation (GO:0030168)	55	4	0.19	+	20.78	5.38E-05	1.63E-02
acute inflammatory response (GO:0002526)	70	5	0.25	+	20.41	6.46E-06	3.39E-03
zymogen activation (GO:0031638)	86	5	0.3	+	16.61	1.67E-05	7.10E-03
regulation of blood coagulation (GO:0030193)	71	4	0.25	+	16.09	1.38E-04	3.45E-02
negative regulation of wound healing (GO:0061045)	71	4	0.25	+	16.09	1.38E-04	3.40E-02
regulation of hemostasis (GO:1900046)	73	4	0.26	+	15.65	1.53E-04	3.65E-02
regulation of coagulation (GO:0050818)	75	4	0.26	+	15.24	1.69E-04	3.97E-02
mucopolysaccharide metabolic process (GO:1903510)	77	4	0.27	+	14.84	1.86E-04	4.24E-02
coagulation (GO:0050817)	118	5	0.41	+	12.1	7.10E-05	1.96E-02
blood coagulation (GO:0007596)	118	5	0.41	+	12.1	7.10E-05	1.93E-02
hemostasis (GO:0007599)	120	5	0.42	+	11.9	7.66E-05	2.05E-02
complement activation (GO:0006956)	231	9	0.81	+	11.13	1.55E-07	2.22E-04
negative regulation of peptidase activity (GO:0010466)	261	8	0.91	+	8.76	4.46E-06	2.81E-03
negative regulation of hydrolase activity (GO:0051346)	365	11	1.28	+	8.61	7.40E-08	1.30E-04
positive regulation of immune effector process (GO:0002699)	315	8	1.1	+	7.26	1.69E-05	7.00E-03
humoral immune response (GO:0006959)	436	11	1.53	+	7.21	4.17E-07	3.65E-04
negative regulation of proteolysis (GO:0045861)	360	9	1.26	+	7.14	5.48E-06	2.98E-03
inflammatory response (GO:0006954)	512	12	1.79	+	6.7	2.56E-07	2.69E-04
activation of immune response (GO:0002253)	431	10	1.51	+	6.63	3.04E-06	2.18E-03
regulation of immune effector process (GO:0002697)	455	10	1.59	+	6.28	4.85E-06	2.83E-03
negative regulation of catalytic activity (GO:0043086)	662	13	2.32	+	5.61	5.54E-07	4.59E-04
positive regulation of immune response (GO:0050778)	717	14	2.51	+	5.58	2.05E-07	2.49E-04
regulation of vesicle-mediated transport (GO:0060627)	621	12	2.17	+	5.52	1.87E-06	1.40E-03
regulation of peptidase activity (GO:0052547)	444	8	1.55	+	5.15	1.78E-04	4.12E-02
innate immune response (GO:0045087)	897	16	3.14	+	5.1	8.24E-08	1.30E-04
defense response (GO:0006952)	1554	27	5.44	+	4.96	1.29E-12	2.04E-08
adaptive immune response (GO:0002250)	525	9	1.84	+	4.9	9.93E-05	2.61E-02
defense response to other organism (GO:0098542)	1171	20	4.1	+	4.88	2.89E-09	9.12E-06
immune effector process (GO:0002252)	588	10	2.06	+	4.86	4.24E-05	1.31E-02
response to biotic stimulus (GO:0009607)	1554	24	5.44	+	4.41	3.63E-10	2.86E-06
response to other organism (GO:0051707)	1513	23	5.3	+	4.34	1.28E-09	6.70E-06
response to external biotic stimulus (GO:0043207)	1515	23	5.3	+	4.34	1.31E-09	5.16E-06
regulation of immune response (GO:0050776)	1066	16	3.73	+	4.29	8.08E-07	6.37E-04
regulation of hydrolase activity (GO:0051336)	986	14	3.45	+	4.06	8.18E-06	4.03E-03
response to bacterium (GO:0009617)	922	13	3.23	+	4.03	1.93E-05	7.08E-03
biological process involved in interspecies interaction between organisms (GO:0044419)	1646	23	5.76	+	3.99	6.26E-09	1.64E-05
positive regulation of transport (GO:0051050)	1076	15	3.77	+	3.98	4.60E-06	2.79E-03
positive regulation of immune system process (GO:0002684)	1123	15	3.93	+	3.82	7.65E-06	3.89E-03
negative regulation of protein metabolic process (GO:0051248)	1057	14	3.7	+	3.78	1.77E-05	7.15E-03
regulation of response to external stimulus (GO:0032101)	993	13	3.48	+	3.74	4.14E-05	1.30E-02
negative regulation of molecular function (GO:0044092)	1031	13	3.61	+	3.6	6.05E-05	1.80E-02
immune response (GO:0006955)	1668	19	5.84	+	3.25	3.79E-06	2.59E-03
response to external stimulus (GO:0009605)	2488	28	8.71	+	3.21	9.54E-09	1.88E-05
regulation of immune system process (GO:0002682)	1703	19	5.96	+	3.19	5.11E-06	2.87E-03
response to stress (GO:0006950)	3388	33	11.86	+	2.78	8.77E-09	1.97E-05
regulation of catalytic activity (GO:0050790)	1810	17	6.34	+	2.68	1.51E-04	3.67E-02
immune system process (GO:0002376)	2589	23	9.06	+	2.54	1.77E-05	6.98E-03
positive regulation of response to stimulus (GO:0048584)	2409	21	8.43	+	2.49	6.10E-05	1.78E-02
regulation of response to stimulus (GO:0048583)	4140	31	14.49	+	2.14	1.29E-05	5.81E-03
response to stimulus (GO:0050896)	8804	49	30.82	+	1.59	3.50E-05	1.13E-02
biological regulation (GO:0065007)	12948	62	45.32	+	1.37	6.70E-05	1.92E-02

Up in Aging Veh versus Young Veh_Molecular Function

Analysis Type:	PANTHER Overrepresentation Test (Released 20220712)							
Annotation Version and Release Date:	GO Ontology database DOI: 10.5281/zenodo.6799722 Released 2022-07-01							
Analyzed List:	Client Text Box Input (Mus musculus)							
Reference List:	Mus musculus (all genes in database)							
Test Type:	FISHER							
Correction:	FDR							
GO molecular function complete	Mus musculus - REFLIST (21997)	Client Text Box Input (77)	Client Text Box Input (expected)	Client Text Box Input (over/under)	Client Text Box Input (fold Enrichment)	Client Text Box Input (raw P-value)	Client Text Box Input (FDR)	
serine-type endopeptidase inhibitor activity (GO:0004867)	130	7	0.46	+	15.38	5.09E-07	2.46E-03	
endopeptidase inhibitor activity (GO:0004866)	216	8	0.76	+	10.58	1.14E-06	2.77E-03	
peptidase inhibitor activity (GO:0030414)	224	8	0.78	+	10.2	1.49E-06	2.40E-03	
endopeptidase regulator activity (GO:0061135)	232	8	0.81	+	9.85	1.92E-06	2.32E-03	
peptidase regulator activity (GO:0061134)	266	8	0.93	+	8.59	5.11E-06	4.94E-03	

Up in Aging Veh versus Young Veh_Cellular Component

Analysis Type:	PANTHER Overrepresentation Test (Released 20220712)						
Annotation Version and Release Date:	GO Ontology database DOI: 10.5281/zenodo.6799722 Released 2022-07-01						
Analyzed List:	Client Text Box Input (Mus musculus)						
Reference List:	Mus musculus (all genes in database)						
Test Type:	FISHER						
Correction:	FDR						
GO cellular component complete	Mus musculus - REFLIST (21997)	Client Text Box Input (77)	Client Text Box Input (expected)	Client Text Box Input (over/under)	Client Text Box Input (fold Enrichment)	Client Text Box Input (raw P-value)	Client Text Box Input (FDR)
complement component C1q complex (GO:0062167)	3	3	0.01	+	> 100	8.10E-07	4.13E-04
classical-complement-pathway C3/C5 convertase complex (GO:0005601)	3	2	0.01	+	> 100	1.19E-04	2.03E-02
fibrinogen complex (GO:0005577)	6	3	0.02	+	> 100	3.38E-06	1.15E-03
complement component C1 complex (GO:0005602)	7	3	0.02	+	> 100	4.81E-06	1.40E-03
blood microparticle (GO:0072562)	10	4	0.04	+	> 100	1.33E-07	9.07E-05
platelet alpha granule (GO:0031091)	21	3	0.07	+	40.81	7.84E-05	1.45E-02
collagen trimer (GO:0005581)	81	5	0.28	+	17.63	1.26E-05	3.23E-03
collagen-containing extracellular matrix (GO:0062023)	396	10	1.39	+	7.21	1.45E-06	5.92E-04
extracellular matrix (GO:0031012)	526	10	1.84	+	5.43	1.67E-05	3.79E-03
external encapsulating structure (GO:0030312)	528	10	1.85	+	5.41	1.73E-05	3.52E-03
extracellular space (GO:0005615)	2135	29	7.47	+	3.88	5.66E-11	5.77E-08
extracellular region (GO:0005576)	2875	34	10.06	+	3.38	2.44E-11	4.98E-08

Up in Aging Veh versus Young Veh_Reactome

Analysis Type:	PANTHER Overrepresentation Test (Released 20220712)
Annotation Version and Release Date:	Reactome version 65 Released 2021-10-01
Analyzed List:	Client Text Box Input (Mus musculus)
Reference List:	Mus musculus (all genes in database)
Test Type:	FISHER
Correction:	FDR

Reactome pathways	Mus musculus - REFLIST (21997)	Client Text Box Input (77)	Client Text Box Input (expected)	Client Text Box Input (over/under)	Client Text Box Input (fold Enrichment)	Client Text Box Input (raw P-value)	Client Text Box Input (FDR)
Activation of C3 and C5 (R-MMU-174577)	6	3	0.02	+	> 100	3.38E-06	9.57E-04
Alternative complement activation (R-MMU-173736)	5	2	0.02	+	> 100	2.49E-04	2.12E-02
Lectin pathway of complement activation (R-MMU-166662)	6	2	0.02	+	95.23	3.32E-04	2.45E-02
GRB2:SOS provides linkage to MAPK signaling for Integrins (R-MMU-354194)	15	3	0.05	+	57.14	3.21E-05	6.06E-03
p130Cas linkage to MAPK signaling for integrins (R-MMU-372708)	15	3	0.05	+	57.14	3.21E-05	5.45E-03
Regulation of TLR by endogenous ligand (R-MMU-5686938)	20	3	0.07	+	42.85	6.87E-05	9.74E-03
Common Pathway of Fibrin Clot Formation (R-MMU-140875)	21	3	0.07	+	40.81	7.84E-05	1.02E-02
Integrin signaling (R-MMU-354192)	27	3	0.09	+	31.74	1.55E-04	1.46E-02
Platelet Aggregation (Plug Formation) (R-MMU-76009)	37	3	0.13	+	23.16	3.67E-04	2.60E-02
Formation of Fibrin Clot (Clotting Cascade) (R-MMU-140877)	38	3	0.13	+	22.55	3.95E-04	2.69E-02
MAP2K and MAPK activation (R-MMU-5674135)	40	3	0.14	+	21.43	4.56E-04	2.98E-02
Complement cascade (R-MMU-166658)	154	10	0.54	+	18.55	2.78E-10	2.36E-07
Peptide hormone metabolism (R-MMU-2980736)	64	4	0.22	+	17.85	9.41E-05	1.00E-02
Initial triggering of complement (R-MMU-166663)	129	8	0.45	+	17.72	2.63E-08	1.12E-05
Regulation of Complement cascade (R-MMU-977606)	145	8	0.51	+	15.76	6.22E-08	2.12E-05
Platelet degranulation (R-MMU-114608)	122	6	0.43	+	14.05	5.72E-06	1.39E-03
Response to elevated platelet cytosolic Ca2+ (R-MMU-76005)	127	6	0.44	+	13.5	7.13E-06	1.52E-03
Post-translational protein phosphorylation (R-MMU-8957275)	116	5	0.41	+	12.31	6.57E-05	1.01E-02
Creation of C4 and C2 activators (R-MMU-166786)	122	5	0.43	+	11.71	8.26E-05	1.00E-02
Regulation of Insulin-like Growth Factor (IGF) transport and uptake by Insulin-like Growth Factor Binding Proteins (IGFBPs) (R-MMU-381426)	122	5	0.43	+	11.71	8.26E-05	9.36E-03
Platelet activation, signaling and aggregation (R-MMU-76002)	251	6	0.88	+	6.83	2.79E-04	2.16E-02
Innate Immune System (R-MMU-168249)	1047	21	3.66	+	5.73	5.92E-11	1.01E-07
Neutrophil degranulation (R-MMU-6798695)	526	9	1.84	+	4.89	1.01E-04	1.01E-02
Immune System (R-MMU-168256)	1720	23	6.02	+	3.82	1.42E-08	8.04E-06
Metabolism of proteins (R-MMU-392499)	1639	16	5.74	+	2.79	1.59E-04	1.42E-02

Down in Aging Veh versus Young Veh_Cellular Component

Analysis Type:	PANTHER Overrepresentation Test (Released 20220712)							
Annotation Version and Release Date:	GO Ontology database DOI: 10.5281/zenodo.6799722 Released 2022-07-01							
Analyzed List:	Client Text Box Input (Mus musculus)							
Reference List:	Mus musculus (all genes in database)							
Test Type:	FISHER							
Correction:	FDR							
GO cellular component complete	Mus musculus - REFLIST (21997)	Client Text Box Input (34)	Client Text Box Input (expected)	Client Text Box Input (over/under)	Client Text Box Input (fold Enrichment)	Client Text Box Input (raw P-value)	Client Text Box Input (FDR)
postsynaptic density, intracellular component (GO:0099092)	32	3	0.05	+	60.65	2.12E-05	4.33E-02
postsynaptic specialization, intracellular component (GO:0099091)	38	3	0.06	+	51.08	3.44E-05	3.51E-02

Up in Aging iMP versus Young Veh_Biological Process

Analysis Type:	PANTHER Overrepresentation Test (Released 20220712)
Annotation Version and Release Date:	GO Ontology database DOI: 10.5281/zenodo.6799722 Released 2022-07-01
Analyzed List:	Client Text Box Input (Mus musculus)
Reference List:	Mus musculus (all genes in database)
Test Type:	FISHER
Correction:	FDR

GO biological process complete	Mus musculu	Client Text Bc	Client Text Bc	Client Text Bc		Client Text Bc	Client Text Bc	Client Text Box Input (FDR)
synapse pruning (GO:0098883)	10	3	0.03	+		> 100	6.52E-06	4.67E-03
fibrinolysis (GO:0042730)	14	4	0.04	+		98.2	1.91E-07	3.01E-04
plasminogen activation (GO:0031639)	12	3	0.03	+		85.93	1.03E-05	6.79E-03
cell junction disassembly (GO:0150146)	12	3	0.03	+		85.93	1.03E-05	6.52E-03
positive regulation of heterotypic cell-cell adhesion (GO:0034116)	14	3	0.04	+		73.65	1.54E-05	8.08E-03
blood coagulation, fibrin clot formation (GO:0072378)	20	4	0.06	+		68.74	6.55E-07	7.94E-04
protein activation cascade (GO:0072376)	25	4	0.07	+		54.99	1.45E-06	1.52E-03
acute-phase response (GO:0006953)	32	5	0.09	+		53.7	7.11E-08	1.60E-04
platelet aggregation (GO:0070527)	27	4	0.08	+		50.92	1.91E-06	1.77E-03
regulation of heterotypic cell-cell adhesion (GO:0034114)	25	3	0.07	+		41.24	7.24E-05	3.00E-02
negative regulation of extrinsic apoptotic signaling pathway via death domain receptors (GO:1902042)	30	3	0.09	+		34.37	1.19E-04	4.28E-02
negative regulation of endothelial cell apoptotic process (GO:2000352)	32	3	0.09	+		32.22	1.43E-04	4.78E-02
negative regulation of blood coagulation (GO:0030195)	45	4	0.13	+		30.55	1.24E-05	7.50E-03
negative regulation of hemostasis (GO:1900047)	46	4	0.13	+		29.89	1.34E-05	7.83E-03
homotypic cell-cell adhesion (GO:0034109)	47	4	0.14	+		29.25	1.45E-05	8.18E-03
negative regulation of coagulation (GO:0050819)	47	4	0.14	+		29.25	1.45E-05	7.90E-03
platelet activation (GO:0030168)	55	4	0.16	+		25	2.60E-05	1.32E-02
acute inflammatory response (GO:0002526)	70	5	0.2	+		24.55	2.59E-06	2.15E-03
zymogen activation (GO:0031638)	86	5	0.25	+		19.98	6.73E-06	4.61E-03
regulation of blood coagulation (GO:0030193)	71	4	0.21	+		19.36	6.71E-05	3.02E-02
negative regulation of wound healing (GO:0061045)	71	4	0.21	+		19.36	6.71E-05	2.94E-02
regulation of hemostasis (GO:1900046)	73	4	0.21	+		18.83	7.44E-05	2.93E-02
regulation of coagulation (GO:0050818)	75	4	0.22	+		18.33	8.22E-05	3.16E-02
negative regulation of response to wounding (GO:1903035)	89	4	0.26	+		15.45	1.55E-04	4.98E-02
complement activation (GO:0006956)	231	6	0.67	+		8.93	6.43E-05	2.98E-02
inflammatory response (GO:0006954)	512	11	1.49	+		7.38	2.86E-07	3.76E-04
negative regulation of hydrolase activity (GO:0051346)	365	7	1.06	+		6.59	9.96E-05	3.74E-02
humoral immune response (GO:0006959)	436	8	1.27	+		6.31	4.17E-05	2.05E-02
regulation of immune effector process (GO:0002697)	455	8	1.32	+		6.04	5.60E-05	2.68E-02
positive regulation of immune response (GO:0050778)	717	12	2.09	+		5.75	1.05E-06	1.18E-03
adaptive immune response (GO:0002250)	525	8	1.53	+		5.24	1.49E-04	4.88E-02
defense response (GO:0006952)	1554	23	4.52	+		5.09	3.20E-11	5.04E-07
innate immune response (GO:0045087)	897	13	2.61	+		4.98	1.71E-06	1.69E-03
defense response to other organism (GO:0098542)	1171	16	3.41	+		4.7	1.88E-07	3.30E-04
regulation of immune response (GO:0050776)	1066	14	3.1	+		4.51	2.01E-06	1.76E-03
response to biotic stimulus (GO:0009607)	1554	20	4.52	+		4.42	9.71E-09	7.65E-05
response to other organism (GO:0051707)	1513	19	4.4	+		4.32	3.74E-08	1.97E-04
response to external biotic stimulus (GO:0043207)	1515	19	4.41	+		4.31	3.82E-08	1.50E-04
positive regulation of immune system process (GO:0002684)	1123	14	3.27	+		4.28	3.67E-06	2.89E-03
response to bacterium (GO:0009617)	922	11	2.68	+		4.1	6.95E-05	2.96E-02
biological process involved in interspecies interaction between organisms (GO:0044419)	1646	19	4.79	+		3.97	1.39E-07	2.75E-04
regulation of immune system process (GO:0002682)	1703	19	4.95	+		3.83	2.36E-07	3.38E-04
regulation of response to external stimulus (GO:0032101)	993	11	2.89	+		3.81	1.33E-04	4.57E-02
response to external stimulus (GO:0009605)	2488	24	7.24	+		3.32	5.30E-08	1.67E-04
immune response (GO:0006955)	1668	15	4.85	+		3.09	7.25E-05	2.93E-02
response to stress (GO:0006950)	3388	28	9.86	+		2.84	6.63E-08	1.74E-04
positive regulation of response to stimulus (GO:0048584)	2409	18	7.01	+		2.57	1.29E-04	4.53E-02
immune system process (GO:0002376)	2589	19	7.53	+		2.52	1.00E-04	3.68E-02
regulation of response to stimulus (GO:0048583)	4140	28	12.05	+		2.32	5.84E-06	4.39E-03

Up in Aging iMP versus Young Veh_Cellular Component

Analysis Type:	PANTHER Overrepresentation Test (Released 20220712)							
Annotation Version and Release Date:	GO Ontology database DOI: 10.5281/zenodo.6799722 Released 2022-07-01							
Analyzed List:	Client Text Box Input (Mus musculus)							
Reference List:	Mus musculus (all genes in database)							
Test Type:	FISHER							
Correction:	FDR							
GO cellular component complete	Mus musculus - REFLIST (21997)	Client Text Box Input (64)	Client Text Box Input (expected)	Client Text Box Input (over/under)	Client Text Box Input (fold Enrichment)	Client Text Box Input (raw P-value)	Client Text Box Input (FDR)
complement component C1q complex (GO:0062167)	3	3	0.01	+	> 100	4.63E-07	2.36E-04
fibrinogen complex (GO:0005577)	6	3	0.02	+	> 100	1.93E-06	7.88E-04
complement component C1 complex (GO:0005602)	7	3	0.02	+	> 100	2.75E-06	8.03E-04
blood microparticle (GO:0072562)	10	4	0.03	+	> 100	6.31E-08	4.29E-05
platelet alpha granule (GO:0031091)	21	3	0.06	+	49.1	4.51E-05	6.57E-03
high-density lipoprotein particle (GO:0034364)	37	4	0.11	+	37.16	6.02E-06	1.23E-03
lipoprotein particle (GO:1990777)	46	4	0.13	+	29.89	1.34E-05	2.49E-03
plasma lipoprotein particle (GO:0034358)	46	4	0.13	+	29.89	1.34E-05	2.28E-03
protein-lipid complex (GO:0032994)	49	4	0.14	+	28.06	1.69E-05	2.66E-03
collagen trimer (GO:0005581)	81	4	0.24	+	16.97	1.09E-04	1.49E-02
collagen-containing extracellular matrix (GO:0062023)	396	9	1.15	+	7.81	2.45E-06	8.32E-04
extracellular matrix (GO:0031012)	526	10	1.53	+	6.53	3.08E-06	7.84E-04
external encapsulating structure (GO:0030312)	528	10	1.54	+	6.51	3.18E-06	7.21E-04
extracellular space (GO:0005615)	2135	26	6.21	+	4.19	7.48E-11	7.63E-08
extracellular region (GO:0005576)	2875	30	8.36	+	3.59	5.62E-11	1.15E-07

Up in Aging iMP versus Young Veh_Reactome

Analysis Type:	PANTHER Overrepresentation Test (Released 20220712)
Annotation Version and Release Date:	Reactome version 65 Released 2021-10-01
Analyzed List:	Client Text Box Input (Mus musculus)
Reference List:	Mus musculus (all genes in database)
Test Type:	FISHER
Correction:	FDR

Reactome pathways	Mus musculus - REFLIST (21997)	Client Text Box Input (64)	Client Text Box Input (expected)	Client Text Box Input (over/under)	Client Text Box Input (fold Enrichment)	Client Text Box Input (raw P-value)	Client Text Box Input (FDR)
GRB2:SOS provides linkage to MAPK signaling for Integrins (R-MMU-354194)	15	3	0.04	+	68.74	1.84E-05	3.48E-03
p130Cas linkage to MAPK signaling for integrins (R-MMU-372708)	15	3	0.04	+	68.74	1.84E-05	3.13E-03
Regulation of TLR by endogenous ligand (R-MMU-5686938)	20	3	0.06	+	51.56	3.96E-05	6.11E-03
Common Pathway of Fibrin Clot Formation (R-MMU-140875)	21	3	0.06	+	49.1	4.51E-05	5.90E-03
Integrin signaling (R-MMU-354192)	27	3	0.08	+	38.19	8.94E-05	1.09E-02
Platelet Aggregation (Plug Formation) (R-MMU-76009)	37	4	0.11	+	37.16	6.02E-06	2.05E-03
Formation of Fibrin Clot (Clotting Cascade) (R-MMU-140877)	38	4	0.11	+	36.18	6.64E-06	1.61E-03
MAP2K and MAPK activation (R-MMU-5674135)	40	4	0.12	+	34.37	8.01E-06	1.70E-03
Platelet degranulation (R-MMU-114608)	122	7	0.35	+	19.72	9.34E-08	1.59E-04
Response to elevated platelet cytosolic Ca2+ (R-MMU-76005)	127	7	0.37	+	18.94	1.21E-07	1.03E-04
Initial triggering of complement (R-MMU-166663)	129	5	0.38	+	13.32	4.39E-05	6.22E-03
Creation of C4 and C2 activators (R-MMU-166786)	122	4	0.35	+	11.27	4.95E-04	4.95E-02
Complement cascade (R-MMU-166658)	154	5	0.45	+	11.16	9.88E-05	1.12E-02
Platelet activation, signaling and aggregation (R-MMU-76002)	251	8	0.73	+	10.95	8.13E-07	3.45E-04
Neutrophil degranulation (R-MMU-6798695)	526	8	1.53	+	5.23	1.50E-04	1.60E-02
Innate Immune System (R-MMU-168249)	1047	15	3.05	+	4.92	2.71E-07	1.54E-04
Immune System (R-MMU-168256)	1720	17	5	+	3.4	6.16E-06	1.75E-03

Down in Aging iMP versus Young Veh_Reactome

Analysis Type:	PANTHER Overrepresentation Test (Released 20220712)
Annotation Version and Release Date:	Reactome version 65 Released 2021-10-01
Analyzed List:	Client Text Box Input (Mus musculus)
Reference List:	Mus musculus (all genes in database)
Test Type:	FISHER
Correction:	FDR

Reactome pathways	Mus musculus - REFLIST (21997)	Client Text Box Input (32)	Client Text Box Input (expected)	Client Text Box Input (over/under)	Client Text Box Input (fold Enrichment)	Client Text Box Input (raw P-value)	Client Text Box Input (FDR)
Erythrocytes take up oxygen and release carbon dioxide (R-MMU-1247673)	8	3	0.01	+	> 100	4.56E-07	7.75E-04
Erythrocytes take up carbon dioxide and release oxygen (R-MMU-1237044)	12	3	0.02	+	> 100	1.25E-06	1.06E-03
O2/CO2 exchange in erythrocytes (R-MMU-1480926)	12	3	0.02	+	> 100	1.25E-06	7.09E-04

Table S3 (Supporting Information). The top 50 proteins in iMP conditioned media. A literature search shows the relevance of these proteins in Alzheimer's disease, inflammation, aging, and cognition.

Protein	Alzheimer's	Inflammation	Aging	Cognition	References
F13A1	X	X			(Gerardino et al., 2006; Friedman et al., 2018)
FTL	X	X	X	X	(Nishida et al., 2014; Kenkhuis et al., 2021; Vulinović et al., 2022; Dai et al., 2023)
VIM	X	X	X		(Cogné et al., 2020; Zhang et al., 2025)
APOE	X	X	X	X	(Kloske and Wilcock, 2020; Torres et al., 2020; Raulin et al., 2022; Zhao et al., 2022)
A2M	X	X	X	X	(Varma et al., 2017; Qiu et al., 2022; Boregowda et al., 2023; Tomihari et al., 2023)
GPNMB	X	X	X	X	(Hüttenrauch et al., 2018; Suda et al., 2022; Liu et al., 2025; Qi et al., 2025)
SPP1	X	X	X	X	(Silvin et al., 2022; De Schepper et al., 2023; Lopes et al., 2024)
PRDX1	X	X	X	X	(Cimini et al., 2013; Chen et al., 2023; Ou et al., 2024; Park et al., 2024)
LCP1	X	X	X		(Carling et al., 2024; Wang et al., 2024; Xie et al., 2024)
PFN1		X	X		(Lu et al., 2020; Rim et al., 2025)
CTSB	X	X	X	X	(Hook et al., 2023; Siddiqui et al., 2024)
PEBP1	X	X	X	X	(Feldmann et al., 2008; Pyo et al., 2018; Kim et al., 2019; Wojdała et al., 2022)
TMSB4X	X	X		X	(Wang et al., 2021; Tang et al., 2024; Tsartsalis et al., 2024)
LGALS1	X	X	X	X	(Starossom et al., 2012; Shen et al., 2021; Kiss et al., 2023; Lu et al., 2023)
MRC1	X	X	X		(Zöller et al., 2018)(Colton et al., 2006)
CTSD	X	X	X	X	(Payton et al., 2006; Kim et al., 2007; Di Domenico et al., 2016; Guo et al., 2018)
PSAP	X	X	X	X	(Mendsaikhan et al., 2019; Sun et al., 2024; Yang et al., 2024)
CTSC	X	X			(Liu et al., 2019, 2024)
CD163	X	X	X	X	(Han et al., 2022; Chen et al., 2024)
S100A4	X	X	X	X	(Swindell et al., 2013; Horvath et al., 2016; Lampinen et al., 2022)

YWHAE	X	X	X	X	(Wang et al., 2017; Denommé-Pichon et al., 2023; Tao et al., 2024)
MDH1	X	X	X		(Guo et al., 2020; Jia et al., 2023)
CSTB	X	X		X	(Pollari et al., 2023; Wu et al., 2023; Gumusgoz et al., 2024)
FCGBP		X			(Gómez-Garre et al., 2022)
TPM4			X		(Suchowerska et al., 2017)
CST3	X		X	X	(Hua et al., 2012; Maniwa et al., 2020)
CAPG	X				(Askenazi et al., 2023)
FTH1	X		X		(Bao et al., 2021)
S100A11	X	X			(Xia et al., 2018)
ALDOA	X		X		(Li et al., 2021)
YWHAZ	X			X	(Oh et al., 2025)
PPIA	X				(Blair et al., 2015)
AIF1	X	X	X	X	(Roy et al., 2020)
IDH1		X			(Amankulor et al., 2017)
COTL1	X	X			(Rangaraju et al., 2018)
HIST4H4	X			X	(Plagg et al., 2015)
PRDX6			X		(Lubec et al., 2019; Pacifici et al., 2020)
GSN	X	X	X	X	(Gao et al., 2017; Piktel et al., 2018; Jiang et al., 2023)
TPI1			X		(Ralser et al., 2008)
FKBP1A			X		(Hassani et al., 2022)
LAMP1	X	X	X	X	(Rovira et al., 2022; Meca-Laguna et al., 2025)
VAT1			X		(Bi et al., 2025)
PLTP	X		X		(Tong et al., 2015)
C1QB	X		X	X	(Dubey et al., 2025; Ishikawa et al., 2025)
ANXA5		X	X		(Klement et al., 2012; Jia et al., 2024)
VSIG4	X	X	X	X	(Cai et al., 2024; Huang et al., 2025; Yang et al., 2025)
B2M	X	X	X	X	(Zhong et al., 2020; Zhao et al., 2023; Ke et al., 2025)
GAPDH	X	X	X	X	(Butterfield et al., 2010; Lazarev et al., 2021; Schmalhausen et al., 2024)
C1QC	X	X	X	X	(Dejanovic et al., 2022; Wen et al., 2024; Xu et al., 2025)

ARPC4	X				(Pelucchi et al., 2020)

References

Amankulor NM, Kim Y, Arora S, Kargl J, Szulzewsky F, Hanke M, Margineantu DH, Rao A, Bolouri H, Delrow J, Hockenbery D, Houghton AM, Holland EC (2017) Mutant IDH1 regulates the tumor-associated immune system in gliomas. Genes Dev 31:774–786.

Askenazi M, Kavanagh T, Pires G, Ueberheide B, Wisniewski T, Drummond E (2023) Compilation of reported protein changes in the brain in Alzheimer's disease. Nat Commun 14:4466.

Bao W-D, Pang P, Zhou X-T, Hu F, Xiong W, Chen K, Wang J, Wang F, Xie D, Hu Y-Z, Han Z-T, Zhang H-H, Wang W-X, Nelson PT, Chen J-G, Lu Y, Man H-Y, Liu D, Zhu L-Q (2021) Loss of ferroportin induces memory impairment by promoting ferroptosis in Alzheimer's disease. Cell Death Differ 28:1548–1562.

Bi K, Chen Y, Hu Y, Li S, Li W, Yu Z, Yu L (2025) Comprehensive bioinformatics analysis reveals novel potential biomarkers associated with aging and mitochondria in osteoporosis. Sci Rep 15:934.

Blair LJ, Baker JD, Sabbagh JJ, Dickey CA (2015) The emerging role of peptidyl-prolyl isomerase chaperones in tau oligomerization, amyloid processing, and Alzheimer's disease. J Neurochem 133:1–13.

Boregowda SV, Haga CL, Supper VM, Booker CN, Phinney DG (2023) Novel role for alpha-2-macroglobulin (A2M) as a disease modifying protein in senile osteoporosis. Front Cell Dev Biol 11:1294438.

Butterfield DA, Hardas SS, Lange MLB (2010) Oxidatively modified glyceraldehyde-3-phosphate dehydrogenase (GAPDH) and Alzheimer's disease: many pathways to neurodegeneration. J Alzheimers Dis 20:369–393.

Cai P, Wang J, Xu J, Zhang M, Yin X, He S, Zhuang J (2024) V-set and immunoglobulin domain containing 4 inhibits oxidative stress, mitochondrial dysfunction, and inflammation to attenuate Parkinson's disease progression by activating the JAK2/STAT3 pathway. J Neuroimmunol 391:578345.

Carling GK et al. (2024) Alzheimer's disease-linked risk alleles elevate microglial cGAS-associated senescence and neurodegeneration in a tauopathy model. Neuron 112:3877-3896.e8.

Chen Q, Thompson J, Hu Y, Lesnefsky EJ (2023) Endoplasmic reticulum stress and alterations of peroxiredoxins in aged hearts. Mech Ageing Dev 215:111859.

Chen Y, Lu P, Wu S, Yang J, Liu W, Alzheimer's Disease Neuroimaging Initiative, Zhang Z, Xu Q (2024) CD163-Mediated Small-Vessel Injury in Alzheimer's Disease: An Exploration from Neuroimaging to Transcriptomics. Int J Mol Sci 25.

Cimini A, Gentile R, Angelucci F, Benedetti E, Pitari G, Giordano A, Ippoliti R (2013) Neuroprotective effects of PrxI over-expression in an in vitro human Alzheimer's disease model. J Cell Biochem 114:708–715.

Cogné B, Bouameur J-E, Hayot G, Latypova X, Pattabiraman S, Caillaud A, Si-Tayeb K, Besnard T, Küry S, Chariau C, Gaignerie A, David L, Bordure P, Kaganovich D, Bézieau S, Golzio C, Magin TM, Isidor B (2020) A dominant vimentin variant causes a rare syndrome with premature aging. Eur J Hum Genet 28:1218–1230.

Colton CA, Mott RT, Sharpe H, Xu Q, Van Nostrand WE, Vitek MP (2006) Expression profiles for macrophage alternative activation genes in AD and in mouse models of AD. J Neuroinflammation 3:27.

Dai DL, Li M, Lee EB (2023) Human Alzheimer's disease reactive astrocytes exhibit a loss of homeostastic gene expression. Acta Neuropathol Commun 11:127.

Dejanovic B et al. (2022) Complement C1q-dependent excitatory and inhibitory synapse elimination by astrocytes and microglia in Alzheimer's disease mouse models. Nat Aging 2:837–850.

De Schepper S, Ge JZ, Crowley G, Ferreira LSS, Garceau D, Toomey CE, Sokolova D, Rueda-Carrasco J, Shin S-H, Kim J-S, Childs T, Lashley T, Burden JJ, Sasner M, Sala Frigerio C, Jung S, Hong S (2023) Perivascular cells induce microglial phagocytic states and synaptic engulfment via SPP1 in mouse models of Alzheimer's disease. Nat Neurosci 26:406–415.

Denommé-Pichon A-S et al. (2023) YWHAE loss of function causes a rare neurodevelopmental disease with brain abnormalities in human and mouse. Genet Med 25:100835.

Di Domenico F, Tramutola A, Perluigi M (2016) Cathepsin D as a therapeutic target in Alzheimer's disease. Expert Opin Ther Targets 20:1393–1395.

Dubey SK, Dubey R, Jung K, Hernandez AG, Kleinman ME (2025) Deciphering age-related transcriptomic changes in the mouse retinal pigment epithelium. Aging (Albany NY) 17:657–684.

Feldmann RE, Maurer MH, Hunzinger C, Lewicka S, Buergers HF, Kalenka A, Hinkelbein J, Broemme JO, Seidler GH, Martin E, Plaschke K (2008) Reduction in rat phosphatidylethanolamine binding protein-1 (PEBP1) after chronic corticosterone treatment may be paralleled by cognitive impairment: a first study. Stress 11:134–147.

Friedman BA, Srinivasan K, Ayalon G, Meilandt WJ, Lin H, Huntley MA, Cao Y, Lee S-H, Haddick PCG, Ngu H, Modrusan Z, Larson JL, Kaminker JS, van der Brug MP, Hansen DV (2018) Diverse brain myeloid expression profiles reveal distinct microglial activation states and aspects of alzheimer's disease not evident in mouse models. Cell Rep 22:832–847.

Gao J, Qin Z, Guan X, Guo J, Wang H, Liu S (2017) Overexpression of GSN could decrease inflammation and apoptosis in EAE and may enhance vitamin D therapy on EAE/MS. Sci Rep 7:604.

Gerardino L, Papaleo P, Flex A, Gaetani E, Fioroni G, Pola P, Pola R (2006) Coagulation factor XIII Val34Leu gene polymorphism and Alzheimer's disease. Neurol Res 28:807–809.

Gómez-Garre P, Periñán MT, Jesús S, Bacalini MG, Garagnani P, Mollenhauer B, Pirazzini C, Provini F, Trenkwalder C, Franceschi C, Mir P, PROPAG-AGEING consortium (2022) Transcriptomic analysis reveals an association of FCGBP with Parkinson's disease. npj Parkinsons Disease 8:157.

Gumusgoz E, Kasiri S, Verma M, Wu J, Villarreal Acha D, Marriam U, Fyffe-Maricich S, Lin A, Chen X, Gray SJ, Minassian BA (2024) CSTB gene replacement improves neuroinflammation, neurodegeneration and ataxia in murine type 1 progressive myoclonus epilepsy. Gene Ther 31:234–241.

Guo B, Chen Y, Qi P, Ye Y, Lv Z, Zhang X, Xu K, Yu Chen, Pengzhi Qi, Yingying Ye, Zhenming Lv, Xiumei Zhang, Kaida Xu (2018) Preliminary Study of the Potential Mechanism of CTSD in the Aging Process of Sepiella japonica: Fundamental Function Analysis. BioRxiv.

Guo X, Park JE, Gallart-Palau X, Sze SK (2020) Oxidative damage to the TCA cycle enzyme MDH1 dysregulates bioenergetic enzymatic activity in the aged murine brain. J Proteome Res 19:1706–1717.

Han X, Liu Y-J, Liu B-W, Ma Z-L, Xia T-J, Gu X-P (2022) TREM2 and CD163 Ameliorate Microglia-Mediated Inflammatory Environment in the Aging Brain. J Mol Neurosci 72:1075–1084.

Hassani B, Goshtasbi G, Nooraddini S, Firouzabadi N (2022) Pharmacological approaches to decelerate aging: A promising path. Oxid Med Cell Longev 2022:4201533.

Hook G, Kindy M, Hook V (2023) Cathepsin B Deficiency Improves Memory Deficits and Reduces Amyloid-β in hAβPP Mouse Models Representing the Major Sporadic Alzheimer's Disease Condition. J Alzheimers Dis 93:33–46.

Horvath I, Jia X, Johansson P, Wang C, Moskalenko R, Steinau A, Forsgren L, Wågberg T, Svensson J, Zetterberg H, Morozova-Roche LA (2016) Pro-inflammatory S100A9 Protein as a Robust Biomarker Differentiating Early Stages of Cognitive Impairment in Alzheimer's Disease. ACS Chem Neurosci 7:34–39.

Hua Y, Zhao H, Lu X, Kong Y, Jin H (2012) Meta-analysis of the cystatin C(CST3) gene G73A polymorphism and susceptibility to Alzheimer's disease. Int J Neurosci 122:431–438.

Huang X, Jannu AJ, Song Z, Jury-Garfe N, Lasagna-Reeves CA, Alzheimer's Disease Neuroimaging Initiative, Johnson TS, Huang K, Zhang J (2025) Predicting Alzheimer's disease subtypes and understanding their molecular characteristics in living patients with transcriptomic trajectory profiling. Alzheimers Dement 21:e14241.

Hüttenrauch M, Ogorek I, Klafki H, Otto M, Stadelmann C, Weggen S, Wiltfang J, Wirths O (2018) Glycoprotein NMB: a novel Alzheimer's disease associated marker expressed in a subset of activated microglia. Acta Neuropathol Commun 6:108.

Ishikawa H, Hoshino T, Hamanaka G, Mandeville ET, Guo S, Kimura S, Fukuda N, Li W, Shindo A, Sakadzic S, Harrington ME, Lo EH, Arai K (2025) Effects of aging on diurnal transcriptome change in the mouse corpus callosum. iScience 28:111556.

Jiang Y et al. (2023) GSN gene frameshift mutations in Alzheimer's disease. J Neurol Neurosurg Psychiatr 94:436–447.

Jia D, Wang F, Yu H (2023) Systemic alterations of tricarboxylic acid cycle enzymes in Alzheimer's disease. Front Neurosci 17:1206688.

Jia Z, Kang B, Dong Y, Fan M, Li W, Zhang W (2024) Annexin A5 Derived from Cell-free Fat Extract Attenuates Osteoarthritis via Macrophage Regulation. Int J Biol Sci 20:2994–3007.

Ke Y, Chen P, Wu C, Wang Q, Zeng K, Liang M (2025) β2-microglobulin and cognitive decline: unraveling the mediating role of the Dunedin Pace of Aging methylation. Front Aging Neurosci 17:1505185.

Kenkhuis B, Somarakis A, de Haan L, Dzyubachyk O, IJsselsteijn ME, de Miranda NFCC, Lelieveldt BPF, Dijkstra J, van Roon-Mom WMC, Höllt T, van der Weerd L (2021) Iron loading is a prominent feature of activated microglia in Alzheimer's disease patients. Acta Neuropathol Commun 9:27.

Kim S, Ock J, Kim AK, Lee HW, Cho J-Y, Kim DR, Park J-Y, Suk K (2007) Neurotoxicity of microglial cathepsin D revealed by secretome analysis. J Neurochem 103:2640–2650.

Kim W, Cho SB, Jung HY, Yoo DY, Oh JK, Choi G-M, Cho T-G, Kim DW, Hwang IK, Choi SY, Moon SM (2019) Phosphatidylethanolamine-Binding Protein 1 Ameliorates Ischemia-Induced Inflammation and Neuronal Damage in the Rabbit Spinal Cord. Cells 8.

Kiss T, Mir Y, Stefancsik G, Ganbat G, Askarova A, Monostori E, Dulka K, Szebeni GJ, Nyúl-Tóth Á, Csiszár A, Legradi A (2023) Galectin-1 as a marker for microglia activation in the aging brain. Brain Res 1818:148517.

Klement K, Melle C, Murzik U, Diekmann S, Norgauer J, Hemmerich P (2012) Accumulation of annexin A5 at the nuclear envelope is a biomarker of cellular aging. Mech Ageing Dev 133:508–522.

Kloske CM, Wilcock DM (2020) The important interface between apolipoprotein E and neuroinflammation in alzheimer's disease. Front Immunol 11:754.

Lampinen R et al. (2022) Neuron-astrocyte transmitophagy is altered in Alzheimer's disease. Neurobiol Dis 170:105753.

Lazarev VF, Tsolaki M, Mikhaylova ER, Benken KA, Shevtsov MA, Nikotina AD, Lechpammer M, Mitkevich VA, Makarov AA, Moskalev AA, Kozin SA, Margulis BA, Guzhova IV, Nudler E (2021) Extracellular GAPDH Promotes Alzheimer Disease Progression by Enhancing Amyloid-β Aggregation and Cytotoxicity. Aging Dis 12:1223–1237.

Liu M et al. (2025) GPNMB and ATP6V1A interact to mediate microglia phagocytosis of multiple types of pathological particles. Cell Rep 44:115343.

Liu Q, Zhang Y, Liu S, Liu Y, Yang X, Liu G, Shimizu T, Ikenaka K, Fan K, Ma J (2019) Cathepsin C promotes microglia M1 polarization and aggravates neuroinflammation via activation of Ca2+-dependent PKC/p38MAPK/NF-κB pathway. J Neuroinflammation 16:10.

Liu X-H, Liu X-T, Wu Y, Li S-A, Ren K-D, Cheng M, Huang B, Yang Y, Liu P-P (2024) Broadening horizons: exploring the cathepsin family as therapeutic targets for alzheimer's disease. Aging Dis 16:1987–2007.

Li M, Geng R, Li C, Meng F, Zhao H, Liu J, Dai J, Wang X (2021) Dysregulated gene-associated biomarkers for Alzheimer's disease and aging. Transl Neurosci 12:83–95.

Lopes K de P, Yu L, Shen X, Qiu Y, Tasaki S, Iatrou A, Beeri MS, Seyfried NT, Menon V, Wang Y, Schneider JA, Cantor H, Bennett DA (2024) Associations of cortical SPP1 and ITGAX with cognition and common neuropathologies in older adults. Alzheimers Dement 20:525–537.

Lu E, Wang Q, Li S, Chen C, Wu W, Xu YXZ, Zhou P, Tu W, Lou X, Rao G, Yang G, Jiang S, Zhou K (2020) Profilin 1 knockdown prevents ischemic brain damage by promoting M2 microglial polarization associated with the RhoA/ROCK pathway. J Neurosci Res 98:1198–1212.

Lu Y, Saibro-Girardi C, Fitz NF, McGuire MR, Ostach MA, Mamun-Or-Rashid ANM, Lefterov I, Koldamova R (2023) Multi-transcriptomics reveals brain cellular responses to peripheral infection in Alzheimer's disease model mice. Cell Rep 42:112785.

Lubec J, Smidak R, Malikovic J, Feyissa DD, Korz V, Höger H, Lubec G (2019) Dentate gyrus peroxiredoxin 6 levels discriminate aged unimpaired from impaired rats in a spatial memory task. Front Aging Neurosci 11:198.

Maniwa K, Yano S, Sheikh AM, Onoda K, Mitaki S, Isomura M, Mishima S, Yamaguchi S, Nabika T, Nagai A (2020) Association between cystatin C gene polymorphism and the prevalence of white matter lesion in elderly healthy subjects. Sci Rep 10:4688.

Meca-Laguna G, Qiu M, Hou Y, Barkovskaya A, Shankar A, Dixit B, Rae MJ, Boominathan A, Sharma A (2025) Cell-Surface LAMP1 is a Senescence Marker in Aging and Idiopathic Pulmonary Fibrosis. Aging Cell:e70141.

Mendsaikhan A, Tooyama I, Bellier J-P, Serrano GE, Sue LI, Lue L-F, Beach TG, Walker DG (2019) Characterization of lysosomal proteins Progranulin and Prosaposin and their interactions in Alzheimer’s disease and aged brains: increased levels correlate with neuropathology. Acta Neuropathol Commun 7:215.

Nishida K, Garringer HJ, Futamura N, Funakawa I, Jinnai K, Vidal R, Takao M (2014) A novel ferritin light chain mutation in neuroferritinopathy with an atypical presentation. J Neurol Sci 342:173–177.

Oh HS-H et al. (2025) A cerebrospinal fluid synaptic protein biomarker for prediction of cognitive resilience versus decline in Alzheimer’s disease. Nat Med 31:1592–1603.

Ou Y, Shen C, Chen Z, Liu T, Peng Y, Zong D, Ouyang R (2024) TDP43/HDAC6/Prdx1 signaling pathway participated in the cognitive impairment of obstructive sleep apnea via regulating inflammation and oxidative stress. Int Immunopharmacol 127:111350.

Pacifici F, Della-Morte D, Piermarini F, Arriga R, Scioli MG, Capuani B, Pastore D, Coppola A, Rea S, Donadel G, Andreadi A, Abete P, Sconocchia G, Bellia A, Orlandi A, Lauro D (2020) Prdx6 plays a main role in the crosstalk between aging and metabolic sarcopenia. Antioxidants (Basel) 9.

Park J, Won J, Yang E, Seo J, Cho J, Seong JB, Yeo H-G, Kim K, Kim YG, Kim M, Jeon C-Y, Lim KS, Lee D-S, Lee Y (2024) Peroxiredoxin 1 inhibits streptozotocin-induced Alzheimer’s disease-like pathology in hippocampal neuronal cells via the blocking of Ca2+/Calpain/Cdk5-mediated mitochondrial fragmentation. Sci Rep 14:15642.

Payton A, van den Boogerd E, Davidson Y, Gibbons L, Ollier W, Rabbitt P, Worthington J, Horan M, Pendleton N (2006) Influence and interactions of cathepsin D, HLA-DRB1 and APOE on cognitive abilities in an older non-demented population. Genes Brain Behav 5 Suppl 1:23–31.

Pelucchi S, Stringhi R, Marcello E (2020) Dendritic spines in alzheimer’s disease: how the actin cytoskeleton contributes to synaptic failure. Int J Mol Sci 21.

Piktel E, Levental I, Durnaś B, Janmey PA, Bucki R (2018) Plasma gelsolin: indicator of inflammation and its potential as a diagnostic tool and therapeutic target. Int J Mol Sci 19.

Plagg B, Ehrlich D, Kniewallner KM, Marksteiner J, Humpel C (2015) Increased acetylation of histone H4 at lysine 12 (H4K12) in monocytes of transgenic alzheimer’s mice and in human patients. Curr Alzheimer Res 12:752–760.

Pollari E, Tegelberg S, Björklund H, Kälviäinen R, Lehesjoki A-E, Haapalinna A (2023) In depth behavioral phenotyping unravels complex motor disturbances in Cstb-/- mouse, a model for progressive myoclonus epilepsy type 1. Front Behav Neurosci 17:1325051.

Pyo J-H, Jeon H-J, Park J-S, Lee J-S, Chung H-Y, Yoo M-A (2018) Drosophila PEBP1 inhibits intestinal stem cell aging via suppression of ERK pathway. Oncotarget 9:17980–17993.

Qiu G, Cao L, Chen Y-J (2022) Novel heterozygous mutation in alpha-2-macroglobulin (A2M) suppressing the binding of amyloid-β (Aβ). Front Neurol 13:1090900.

Qi X, She Z, Shi X, Peng H, Wang L, Ma J, Li X, Liang K, Fan Y, Sun Y, Zhang M, Gu Q, Chen S, Zheng J, Li X (2025) Increased plasma GPNMB levels in patients with parkinson's disease and cognitive impairment. Sci Rep 15:20684.

Ralser M, Nebel A, Kleindorp R, Krobitsch S, Lehrach H, Schreiber S, Reinhardt R, Timmermann B (2008) Sequencing and genotypic analysis of the triosephosphate isomerase (TPI1) locus in a large sample of long-lived Germans. BMC Genet 9:38.

Rangaraju S, Dammer EB, Raza SA, Gao T, Xiao H, Betarbet R, Duong DM, Webster JA, Hales CM, Lah JJ, Levey AI, Seyfried NT (2018) Quantitative proteomics of acutely-isolated mouse microglia identifies novel immune Alzheimer's disease-related proteins. Mol Neurodegener 13:34.

Raulin A-C, Doss SV, Trottier ZA, Ikezu TC, Bu G, Liu C-C (2022) ApoE in Alzheimer's disease: pathophysiology and therapeutic strategies. Mol Neurodegener 17:72.

Rim C, Sung S, Kim H-J, Kim SH, Nahm M, Kwon M-S (2025) Nuclear Profilin-1 for DNA Damage Repair Is Involved in Phagocytic Impairment of Senescent Microglia. Glia 73:1707–1726.

Rovira M, Sereda R, Pladevall-Morera D, Ramponi V, Marin I, Maus M, Madrigal-Matute J, Díaz A, García F, Muñoz J, Cuervo AM, Serrano M (2022) The lysosomal proteome of senescent cells contributes to the senescence secretome. Aging Cell 21:e13707.

Roy ER, Wang B, Wan Y-W, Chiu G, Cole A, Yin Z, Propson NE, Xu Y, Jankowsky JL, Liu Z, Lee VM-Y, Trojanowski JQ, Ginsberg SD, Butovsky O, Zheng H, Cao W (2020) Type I interferon response drives neuroinflammation and synapse loss in Alzheimer disease. J Clin Invest 130:1912–1930.

Schmalhausen EV, Medvedeva MV, Muronetz VI (2024) Glyceraldehyde-3-phosphate dehydrogenase is involved in the pathogenesis of Alzheimer's disease. Arch Biochem Biophys 758:110065.

Shen Z, Xu H, Song W, Hu C, Guo M, Li J, Li J (2021) Galectin-1 ameliorates perioperative neurocognitive disorders in aged mice. CNS Neurosci Ther 27:842–856.

Siddiqui AA, Merquiol E, Bruck-Haimson R, Hirbawi J, Boocholez H, Cohen I, Yan Y, Dong MQ, Blum G, Cohen E (2024) Cathepsin B promotes Aβ proteotoxicity by modulating aging regulating mechanisms. Nat Commun 15:8564.

Silvin A et al. (2022) Dual ontogeny of disease-associated microglia and disease inflammatory macrophages in aging and neurodegeneration. Immunity 55:1448-1465.e6.

Starossom SC, Mascanfroni ID, Imitola J, Cao L, Raddassi K, Hernandez SF, Bassil R, Croci DO, Cerliani JP, Delacour D, Wang Y, Elyaman W, Khoury SJ, Rabinovich GA (2012) Galectin-1 deactivates classically activated microglia and protects from inflammation-induced neurodegeneration. Immunity 37:249–263.

Suchowerska AK, Fok S, Stefen H, Gunning PW, Hardeman EC, Power J, Fath T (2017) Developmental Profiling of Tropomyosin Expression in Mouse Brain Reveals Tpm4.2 as the Major Post-synaptic Tropomyosin in the Mature Brain. Front Cell Neurosci 11:421.

Suda M, Shimizu I, Katsuumi G, Hsiao CL, Yoshida Y, Matsumoto N, Yoshida Y, Katayama A, Wada J, Seki M, Suzuki Y, Okuda S, Ozaki K, Nakanishi-Matsui M, Minamino T (2022) Glycoprotein nonmetastatic melanoma protein B regulates lysosomal integrity and lifespan of senescent cells. Sci Rep 12:6522.

Sun Y, Islam S, Michikawa M, Zou K (2024) Presenilin: A Multi-Functional Molecule in the Pathogenesis of Alzheimer's Disease and Other Neurodegenerative Diseases. Int J Mol Sci 25.

Swindell WR, Johnston A, Xing X, Little A, Robichaud P, Voorhees JJ, Fisher G, Gudjonsson JE (2013) Robust shifts in S100a9 expression with aging: a novel mechanism for chronic inflammation. Sci Rep 3:1215.

Tang L, Jin Y, Wang J, Lu X, Xu M, Xiang M (2024) TMSB4X is a regulator of inflammation-associated ferroptosis, and promotes the proliferation, migration and invasion of hepatocellular carcinoma cells. Discov Oncol 15:671.

Tao Q-Q, Cai X, Xue Y-Y, Ge W, Yue L, Li X-Y, Lin R-R, Peng G-P, Jiang W, Li S, Zheng K-M, Jiang B, Jia J-P, Guo T, Wu Z-Y (2024) Alzheimer's disease early diagnostic and staging biomarkers revealed by large-scale cerebrospinal fluid and serum proteomic profiling. Innovation (Camb) 5:100544.

Tomihari A, Kiyota M, Matsuura A, Itakura E (2023) Alpha 2-macroglobulin acts as a clearance factor in the lysosomal degradation of extracellular misfolded proteins. Sci Rep 13:4680.

Tong Y, Sun Y, Tian X, Zhou T, Wang H, Zhang T, Zhan R, Zhao L, Kuerban B, Li Z, Wang Q, Jin Y, Fan D, Guo X, Han H, Qin S, Chui D (2015) Phospholipid transfer protein (PLTP) deficiency accelerates memory dysfunction through altering amyloid precursor protein (APP) processing in a mouse model of Alzheimer's disease. Hum Mol Genet 24:5388–5403.

Torres ERS, Weber Boutros S, Meshul CK, Raber J (2020) ApoE isoform-specific differences in behavior and cognition associated with subchronic MPTP exposure. Learn Mem 27:372–379.

Tsartsalis S et al. (2024) A single nuclear transcriptomic characterisation of mechanisms responsible for impaired angiogenesis and blood-brain barrier function in Alzheimer's disease. Nat Commun 15:2243.

Varma VR, Varma S, An Y, Hohman TJ, Seddighi S, Casanova R, Beri A, Dammer EB, Seyfried NT, Pletnikova O, Moghekar A, Wilson MR, Lah JJ, O'Brien RJ, Levey AI, Troncoso JC, Albert MS, Thambisetty M (2017) Alpha-2 macroglobulin in Alzheimer's disease: a marker of neuronal injury through the RCAN1 pathway. Mol Psychiatry 22:13–23.

Vulinović MP, Turčić P, Micek V, Ljubojević M (2022) Light and heavy ferritin chain expression in the liver and kidneys of Wistar rats: aging, sex differences, and impact of gonadectomy. Arh Hig Rada Toksikol 73:48–61.

Wang J, Li Q, Kong Y, Zhou F, Li J, Li W, Wang K, Wu T, Guan Y, Xie J, Wen T (2017) Biosystems Study of the Molecular Networks Underlying Hippocampal Aging Progression and Anti-aging Treatment in Mice. Front Aging Neurosci 9:393.

Wang M, Feng L-R, Li Z-L, Ma K-G, Chang K-W, Chen X-L, Yang P-B, Ji S-F, Ma Y-B, Han H, Ruganzua JB, Yang W-N, Qian Y-H (2021) Thymosin β4 reverses phenotypic polarization of glial cells and cognitive impairment via negative regulation of NF-κB signaling axis in APP/PS1 mice. J Neuroinflammation 18:146.

Wang Y, Yin Q, Yang D, Jin H, Yao Y, Song J, Liu C, Nie Y, Yin H, Wang W, Xu B, Xue L, Ji X, Chen X, Zhao H (2024) LCP1 knockdown in monocyte-derived macrophages: mitigating ischemic brain injury and shaping immune cell signaling and metabolism. Theranostics 14:159–175.

Wen L, Bi D, Shen Y (2024) Complement-mediated synapse loss in Alzheimer's disease: mechanisms and involvement of risk factors. Trends Neurosci 47:135–149.

Wojdała AL, Chiasserini D, Bellomo G, Paciotti S, Gaetani L, Paoletti FP, Parnetti L (2022) Phosphatidylethanolamine binding protein 1 (PEBP1) in alzheimer's disease: ELISA development and clinical validation. J Alzheimers Dis 88:1459–1468.

Wu Y, Mumford P, Noy S, Cleverley K, Mrzyglod A, Luo D, van Dalen F, Verdoes M, Fisher EMC, Wiseman FK (2023) Cathepsin B abundance, activity and microglial localisation in Alzheimer's disease-Down syndrome and early onset Alzheimer's disease; the role of elevated cystatin B. Acta Neuropathol Commun 11:132.

Xia Q, Li X, Zhou H, Zheng L, Shi J (2018) S100A11 protects against neuronal cell apoptosis induced by cerebral ischemia via inhibiting the nuclear translocation of annexin A1. Cell Death Dis 9:657.

Xie W, Cui Y, Yue L, Zhang T, Huang C, Yu X, Ma D, Liu D, Cheng R, Zhao X, Li X (2024) m6A Reader HNRNPC Facilitates Adipogenesis by Regulating Cytoskeletal Remodeling through Enhanced Lcp1 mRNA Stability. Aging Dis 16:1080–1098.

Xu Y, Zhang H, Jiao X, Zhang Y, Yin G, Wang C, Du Z, Liang M, Gao X, Gu Z, Jiang Y, Du B, Bi X (2025) Dysregulations of C1QA, C1QB, C1QC and C5AR1 as candidate biomarkers of vascular dementia. npj Aging 11:42.

Yang B, Teymur A, Tang C, Wu T (2025) V-set and immunoglobulin domain containing 4 as a potential predictor of Alzheimer’s disease and advanced aging. J Alzheimers Dis 105:453–463.

Yang C, Sun Z-P, Jiang J, Cai X-L, Wang Y, Wang H, Che C, Tu E, Pan A-H, Zhang Y, Wang X-P, Cui M-Z, Xu X-M, Yan X-X, Zhang Q-L (2024) Increased expression of the proapoptotic presenilin associated protein is involved in neuronal tangle formation in human brain. Sci Rep 14:25274.

Zhang L, Wang J, Yan Y, Xiang L, Zhai X, Cai L, Sun Z, Pi M, Xiong Q, Zhou H, Gui Y, Wang X, Shu X, Xia Y (2025) Vimentin Fragmentation and Its Role in Amyloid-Beta Plaque Deposition in Alzheimer’s Disease. Int J Mol Sci 26.

Zhao H et al. (2022) Destabilizing heterochromatin by APOE mediates senescence. Nat Aging 2:303–316.

Zhao Y, Zheng Q, Hong Y, Gao Y, Hu J, Lang M, Zhang H, Zhou Y, Luo H, Zhang X, Sun H, Yan X-X, Huang TY, Wang Y-J, Xu H, Liu C, Wang X (2023) β2-Microglobulin coaggregates with Aβ and contributes to amyloid pathology and cognitive deficits in Alzheimer’s disease model mice. Nat Neurosci 26:1170–1184.

Zhong Q, Zou Y, Liu H, Chen T, Zheng F, Huang Y, Chen C, Zhang Z (2020) Toll-like receptor 4 deficiency ameliorates β2-microglobulin induced age-related cognition decline due to neuroinflammation in mice. Mol Brain 13:20.

Zöller T, Attaai A, Potru PS, Ruß T, Spittau B (2018) Aged mouse cortical microglia display an activation profile suggesting immunotolerogenic functions. Int J Mol Sci 19.
